# Antennal transcriptomes of three tortricid moths reveal putative conserved chemosensory receptors for social and habitat olfactory cues

**DOI:** 10.1038/srep41829

**Published:** 2017-02-02

**Authors:** Francisco Gonzalez, Peter Witzgall, William B. Walker

**Affiliations:** 1 Chemical Ecology Unit, Department of Plant Protection Biology, Swedish University of Agricultural Sciences, Alnarp, Sweden

## Abstract

Insects use chemical signals to find mates, food and oviposition sites. The main chemoreceptor gene families comprise odorant receptors (ORs), ionotropic receptors (IRs) and gustatory receptors (GRs). Understanding the evolution of these receptors as well as their function will assist in advancing our knowledge of how chemical stimuli are perceived and may consequently lead to the development of new insect management strategies. Tortricid moths are important pests in horticulture, forestry and agriculture around the globe. Here, we characterize chemoreceptors from the three main gene families of three economically important tortricids, based on male antennal transcriptomes using an RNA-Seq approach. We identified 49 ORs, 11 GRs and 23 IRs in the green budworm moth, *Hedya nubiferana;* 49 ORs, 12 GRs and 19 IRs in the beech moth, *Cydia fagiglandana*; and 48 ORs, 11 GRs and 19 IRs in the pea moth, *Cydia nigricana*. Transcript abundance estimation, phylogenetic relationships and molecular evolution rate comparisons with deorphanized receptors of *Cydia pomonella* allow us to hypothesize conserved functions and therefore candidate receptors for pheromones and kairomones.

Chemoreception in insects is mediated by receptor proteins specialized in detecting odorants and tastants that convey information about sexual partners, food location, oviposition sites and the presence of threats such as predators and toxins produced by microorganisms^1^,^2^,^3^. Chemosensory proteins mediate the detection of external stimuli, providing input for downstream processing and behavioral responses through specialized circuits in higher brain centers^4^,^5^,^6^,^7^.

Several molecular actors such as odorant binding proteins (OBPs) and sensory neuron membrane proteins (SNMPs) contribute to the detection of chemical signals^8^,^9^. However, chemoreceptor proteins have been widely accepted as the key determinants in insect olfaction and gustation. Volatile compounds are detected by odorant receptors (ORs) embedded in the membranes of dendrites of neurons inside of sensilla, hair-like structures on the antenna. ORs are tuned to a diverse range of odorants from various sources, including host cues, microbial volatiles and conspecific pheromones^10^. In addition, an ancient and complementary conserved set of olfactory receptors, known as ionotropic receptors (IRs), is tuned mainly to acids and amines^11^,^12^. Gustatory receptors (GRs) are proteins present in membranes of neurons inside of organs such as the proboscis, the ovipositor, tarsi and even in the antennae, and are mostly tuned to non-volatile chemicals^13^.

Insect olfactory receptor repertoires are tuned to chemicals with crucial ecological relevance^3^. This implies that the ecological niches of insects are reflected by their chemoreceptors. Functional studies with *Drosophila melanogaster* and *Anopheles gambiae* have supported this theory; their ORs are mainly tuned to the most abundant chemicals from the hosts of each species: esters and aldehydes in the case of the fruit fly and aromatics in the case of the malaria mosquito^14^. Consequently, species-specific shifts are revealed in the tuning, responsiveness and sensitivity of insect chemoreceptors. A classic example is observed in moth pheromone receptors, PRs which mediate sexual communication. Closely related taxa often use the same basic set of pheromone components, but differences in the specificity and sensitivity of their PRs towards major and minor components enable them to recognize conspecific individuals^15^.

*In silico* studies of putative insect chemoreceptors coupled with their functional characterization facilitates the prediction of behavioral responses of insects towards semiochemicals, opening new possibilites for pest control^16^. Therefore, numerous studies have focused on predicting chemoreceptors from several agricultural pests and disease vectors^17^.

Tortricid moths comprise many economically important species worldwide^18^. Their chemoreceptor-associated genes accordingly are the subject of current studies^19^,^20^,^21^,^22^,^23^. Functional characterization of putative pheromone receptors derived from an antennal transcriptome of codling moth, *Cydia pomonella* L.^20^, has led to the discovery of an OR tuned to pear ester, a kairomone of importance for *C. pomonella* larvae, as well as male and female moths^24^,^25^,^26^. Interestingly, this OR belongs to the so-called PR-clade and its activation by pear ester synergizes the activity of the main pheromone component codlemone, both at antennal lobe and behavioral level^25^,^27^. Furthermore, a deeper transcriptomic study of codling moth has shown the presence of several sex-biased and larval enriched receptors, providing a rich substrate for future studies of receptor function and behavioral importance^28^.

The green budworm moth, *Hedya nubiferana* H. (*dimidioalba* R.) (Tortricidae, Olethreutini) feeds on a variety of deciduous trees and shrubs and causes damage on apple and other Rosacean species. Its pheromone consists of a blend of (*E,E*)-8,10-dodecadienyl acetate (E8,E10–12Ac, codlemone acetate) and the minor acetates (*Z*)- and (*E*)-8-dodecenyl acetate (Z8–12Ac, E8–12Ac)^29^,^30^. Some species of the tortricid tribe Grapholitini, such as beech moth, *Cydia fagiglandana* Z. and pea moth, *Cydia nigricana* F., also use codlemone acetate as their pheromone. In these species, different isomers of codlemone acetate act as synergists or antagonists^31^. Furthermore, green budworm moth is attracted to codlemone and pear ester, two compounds that codling moth also utilizes^32^. The chemical ecology of tortricid moths has been studied thoroughly, especially for the development of monitoring and control methods^33^,^34^,^35^. This is a fertile ground for transcriptomic analyses aimed towards functional characterization of chemoreceptors and their phylogenetic divergence. Comparison of orthologous receptors between closely related species has emerged as a good model for yielding insights on the evolution of pheromone receptors as well as unveiling functions of host-sensing receptors^36^,^37^,^38^. Surprisingly, there are relatively few reports studying the evolution of ORs between taxonomically related species, feeding on different host plants^37^,^39^.

Here we use transcriptomic, molecular and bioinformatic approaches to report chemoreceptors of the three main gene families for the tortricid species *H. nubiferana, C. fagiglandana* and *C. nigricana*, and predict putative functions based on comparisons with orthologous receptors of *C. pomonella.*

## Results

### Sequencing and transcriptome assembly

Through use of an Illumina HiSeq2500 approach, three RNA-seq libraries were generated, from male antennal samples of three tortricid moths. For *H. nubiferana*, transcriptomic assembly produced 107,543 contig gene clusters, while for *C. fagiglandana* and *C. nigricana* 100,279 and 137,083 contig gene clusters were produced respectively (Table 1).

Analysis of the most highly expressed genes in the antennae of each species showed an enriched number of transcripts related to mitochondrial functions (29–54%), and odorant binding proteins (15–36%). Transcripts for other chemosensory, ribosomal and hormonal related proteins were highly abundant in all species (Supplementary Figure S1).

### Identification of chemosensory genes and expression

#### Odorant receptors

**Figure 1:**
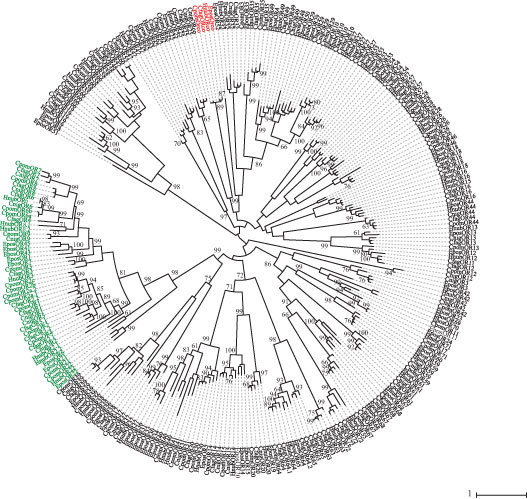
Maximum likelihood tree of candidate ORs of *H. nubiferana (Hnub), C. fagiglandana* (Cfag) and *C. nigricana* (Cnig), along with sequences of *C. pomonella* (Cpom) and *E. postvittana* (Epos). Putative receptors of the Orco clade are colored red; putative receptors of the moth pheromone receptor (PR) clade are colored green. Node support was assessed with 600 bootstrap replicates and values greater than 60% are shown.

Qualitative analysis of the male antennal transcriptomes revealed 49 ORs in *H. nubiferana* (35 full-length ORFs), 49 ORs in *C. fagiglandana* (36 full-length ORFs) and 48 in *C. nigricana* (38 full-length ORFs). These receptors cluster within 11 different clades, according to phylogenetic analysis (Fig. 1; Supplementary Data S1). The three species share at least 34 putatively homologous receptors, including PRs, general ORs and the insect odorant co-receptor (Orco).

A total of 23 predicted sequences cluster within the lepidopteran pheromone receptor clade. Both *Cydia* species share a close ortholog of CpomOR1, while orthologs of CpomOR2, CpomOR6 and CpomOR8 are found in all three species. OR6 is particularly interesting since the amino acid sequence identity between these orthologous receptors ranges between 90–94% (even for HnubOR6, for which the sequence is incomplete), while sequence identity of OR2 and OR8 range between 42.07–61.27 and 45.52–93, respectively.

**Figure 2:**
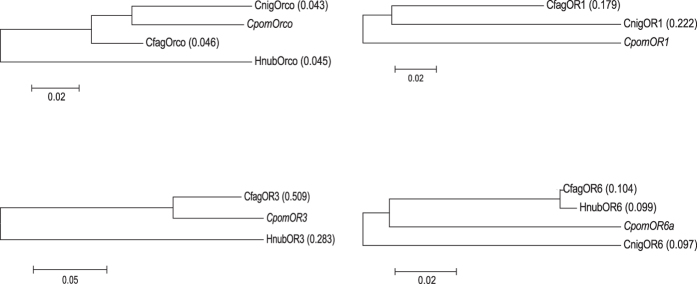
Maximum likelihood trees of selected *C. pomonella* ORs orthologs. dN/dS values of each ortholog in comparison to the corresponding *C. pomonella* OR are shown between parentheses. Comparison between CpomOR1 and HnubOR2.1 is excluded since the proportion of nucleotides that are different is greater than 75% and hence distance cannot be computed for pairwise analysis.

Molecular evolution rate analyses between selected OR homologs in comparison to Cpom ORs provide evidence of purifying selection for Orco, OR1, OR3 and OR6 (dN/dS <1; Fig. 2). Further statistical tests (Z-Test of selection) of this hypothesis supported these results for Orco (*p* < 0.0001; *Z* = 4.734), OR1 (*p* < 0.0001; *Z* = 4.817), OR3 (*p *<* *0.0001; *Z *=* *4.849), and OR6 (*p *<* *0.0001; *Z *=* *9.058). Alternatively, positive selection was not favoured when presented as alternative hypothesis to the null hypothesis of neutrality for the homologs of Orco (*p *=* *1.000; *Z *=* *−6.139), OR1 (*p *=* *1.000; *Z *=* *−4.187), OR3 (*p *=* *1.000; *Z *=* *−6.414), and OR6 (*p *=* *1.000; *Z *=* *−8.526). Furthermore, no evidence of positive selection was found when the homologs were analyzed at the codon level (Supplementary Table S1).

**Figure 3:**
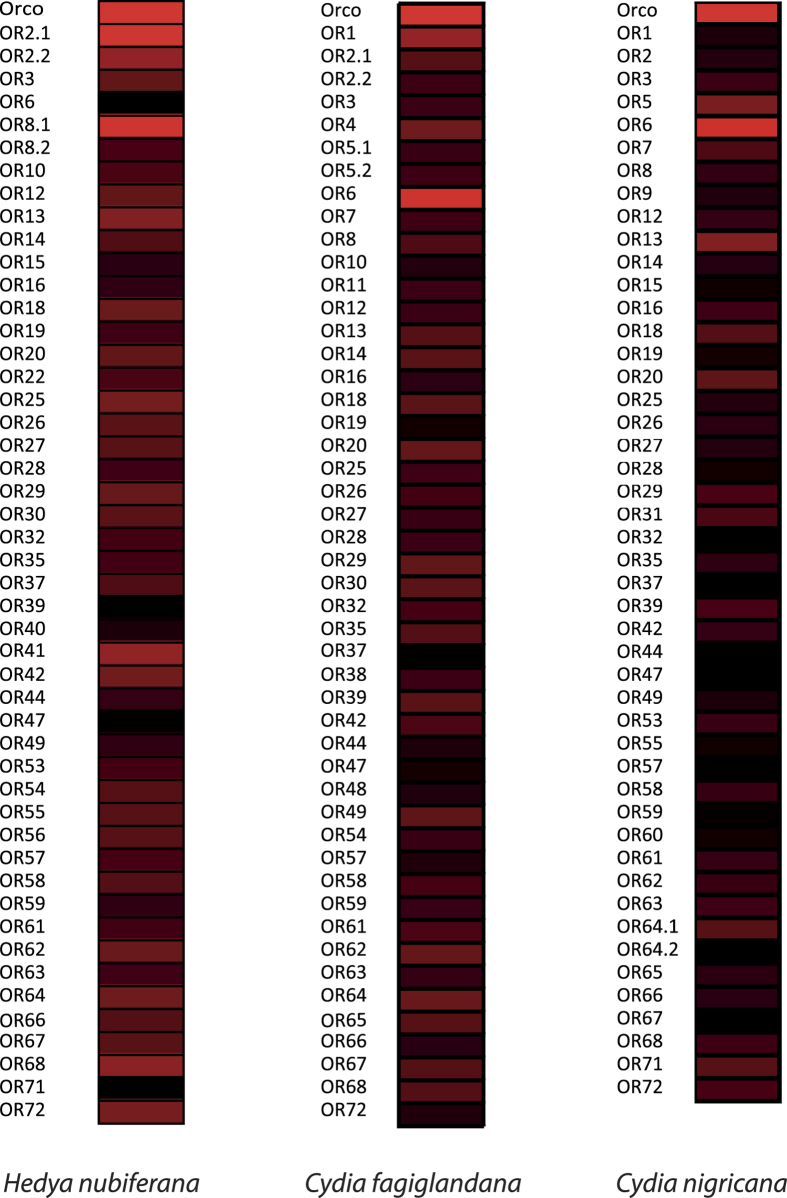
Heat-plot of relative expression values of ORs of *H. nubiferana, C. fagiglandana* and *C. nigricana*. Estimation of abundance values determined by read mapping. Black indicates low/no expression, dark colors indicate low/moderate expression, and bright colors indicate moderate/high expression. Color plots represent binary log of FPKM plus one for each gene (Raw data in Supplementary Table S2).

Quantitative analysis of OR expression shows that for *H. nubiferana*, transcript abundance levels range between 0.58 to 441.88 FPKM, while for *C. fagiglandana* expression levels range between 1.51 to 1768.41 FPKM, and between 16.93 to 985.06 FPKM for *C. nigricana* (Supplementary Table S2). Apart from Orco, the most highly expressed OR for *C. fagiglandana and C. nigricana* is OR6, while the most highly expressed of *H. nubiferana* is OR2.1, followed by OR8.1 (Fig. 3). Some putative non-PRs also show relatively high levels of expression, including HnubOR41, CfagOR64, CnigOR13 and CnigOR20.

**Figure 4:**
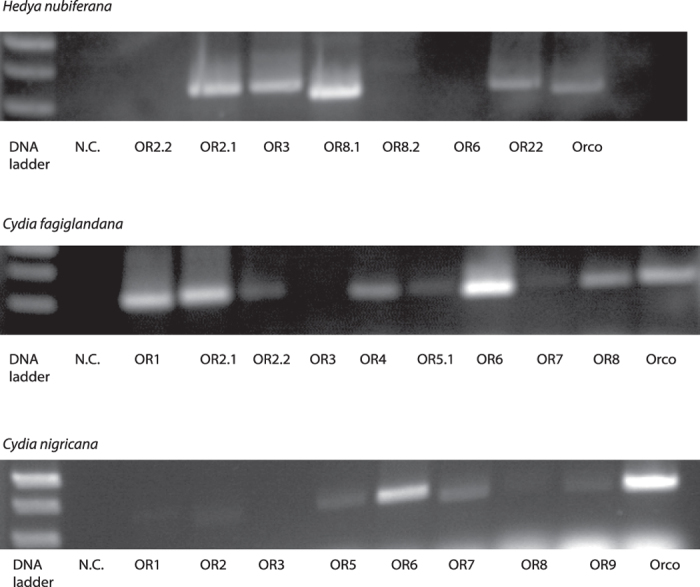
Qualitative visualization of gene expression using antennal cDNA from RNA extracted from male antennae of *H. nubiferana, C. fagiglandana* and *C. nigricana*.

Visualization of RT-PCR products derived from samples of antennal RNA provide further evidence for expression of HnubOR8.1, CfagOR6 and CnigOR6 (Fig. 4).

#### Gustatory receptors

**Figure 5:**
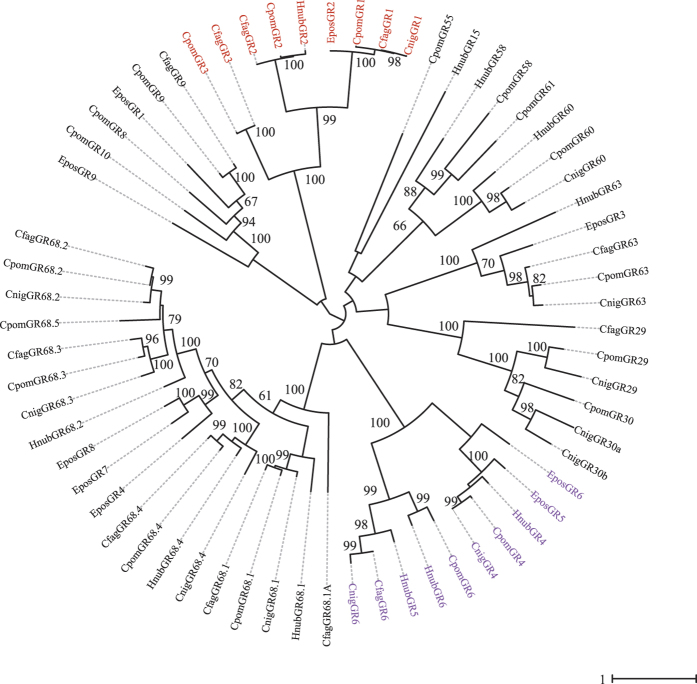
Maximum likelihood tree of candidate GRs of *H. nubiferana* (Hnub), *C. fagiglandana* (Cfag) and *C. nigricana* (Cnig), along with *C. pomonella* (Cpom) and *E. postvittana* (Epos). Putative carbon dioxide receptors are highlighted in red; putative sugar receptors are highlighted in purple; the rest are considered putative bitter receptors. Node support was assessed with 600 bootstrap replicates and values greater than 60% are shown.

A total of 34 sequences were determined to be putative GRs from the species studied, 11 for *H. nubiferana,* 12 for *C. fagiglandana* and 11 for *C. nigricana* (Supplementary Data S1). Only two GR transcripts of the green budworm moth contained complete ORFs based on the presence of start and stop codons, while for the beech moth the number of complete GRs was ten and two in the case of the pea moth. In each species, GRs were identified that cluster in the main subclades of receptors putatively tuned to CO_2_, sugars and bitter substances (Fig. 5).

**Figure 6:**
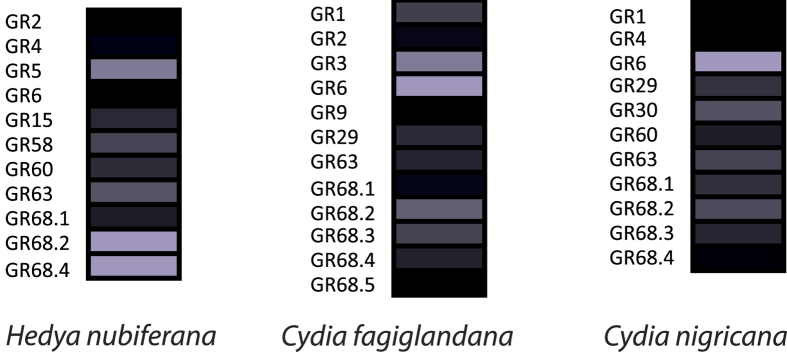
Heat-plot of relative expression values of GRs of *H. nubiferana, C. fagiglandana* and *C. nigricana*. Estimation of abundance values determined by read mapping. Black indicates low/no expression, dark colors indicate low/moderate expression, and bright colors indicate moderate/high expression. Color plots represent binary log of FPKM plus one for each gene (Raw data in Supplementary Table S2).

Quantitative analysis of GR transcript expression shows lower levels than observed for ORs. Different ranges of expression were observed across species. For *H. nubiferana*, transcript abundance levels range between 0.51 to 9.65 FPKM, while for *C. fagiglanda*, between 0.63 to 20.58, and 0.74 to 44.53 for *C. nigricana*. The identity of the most highly expressed GR per species also varied, with GR68.2 being the highest for *Hedya* and GR6 the highest for the *Cydia* species (Fig. 6; Supplementary Table 2).

#### Ionotropic receptors

**Figure 7:**
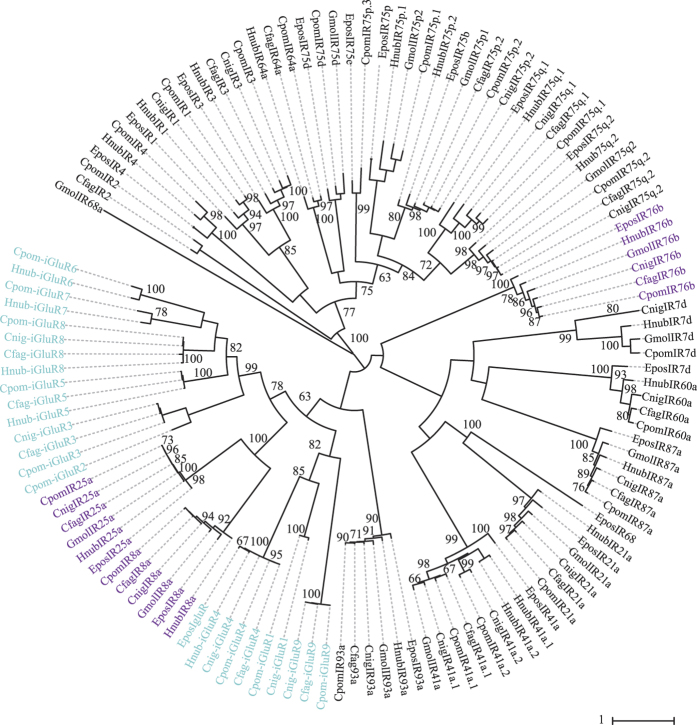
Maximum likelihood tree of candidate IRs/iGluRs of *H. nubiferana* (Hnub), *C. fagiglandana* (Cfag) and *C. nigricana* (Cnig), along with *C. pomonella* (Cpom), *E. postvittana* (Epos) and *G. molesta* (Gmol). Putative ionotropic glutamate receptors (iGluRs) are colored light blue; putative IR co-receptors are colored purple, the rest are considered antennal IRs. Node support was assessed with 600 bootstrap replicates and values greater than 60% are shown.

A total of 61 IRs were identified across the three species of tortricids (Supplementary Data S1). For *H. nubiferana,* 13 out of 23 contain complete ORFs. For *C. fagiglandana,* 13 of 19 present complete ORFs. In the case of *C. nigricana*, the number of complete ORFs is seven out of 19 predicted receptors. All three species contain orthologs for the putative IR co-receptors IR8a, IR25a and IR76b (Fig. 7).

**Figure 8:**
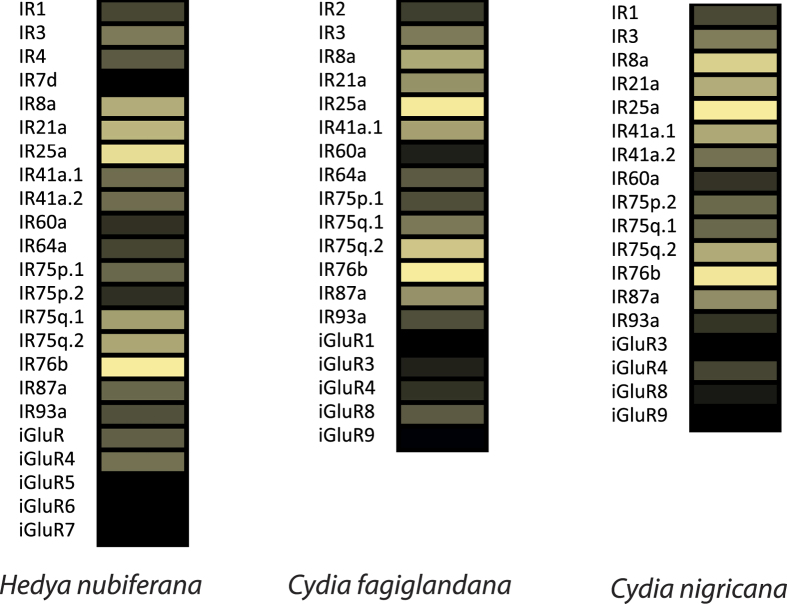
Heat-plot of relative expression values of IRs of *H. nubiferana, C. fagiglandana* and *C. nigricana*. Estimation of abundance values determined by read mapping. Black indicates low/no expression, dark colors indicate low/moderate expression, and bright colors indicate moderate/high expression. Color plots represent binary log of FPKM plus one for each gene (Raw data in Supplementary Table S2).

Transcript abundance analysis showed that, similar to GRs, IR abundance levels were lower than those observed for ORs. For the green budworm moth, abundances ranged between 0.62 to 302.15, while for the beech moth the range was between 0.55 and 259.42 FPKM, and between 1.39 to 160.68 FPKM for the pea moth. In the three species the most highly abundant IRs corresponded to the putative IR co-receptors, followed by IR21a (Fig. 8; Supplementary Table S2).

## Discussion

Prediction of chemosensory receptors through RNA-Seq has become a powerful and reliable methodology to unveil the genes involved in insect olfaction and gustation^28^,^39^,^40^,^41^. We have found a total of 83, 80 and 78 putative chemoreceptors for the green budworm moth *Hedya nubiferana*, the beech moth *Cydia fagiglandana* and the pea moth *C. nigricana*, respectively. This is the first report of the three main chemoreception gene families of these moths^31^,^42^,^43^,^44^.

Since the three species studied were field-collected with pheromone-baited traps, we analysed males only. A comparison of male and female antenna, along with larval tissues, will yield a more complete picture of the chemosensory gene sets of these species, as in a recent study of codling moth^28^. However, this does not undermine the validity of our approach of using only male antennae, especially considering that our assembled transcriptomes are qualitatively and quantitatively consistent with what has been observed in other species^20^,^28^,^45^.

In the three species studied, transcripts associated with mitochondrial and odorant binding proteins were the most abundant (Supplementary Figure S1), similar to what has been observed in the closest reported tortricid, codling moth, *C. pomonella*^28^.

We found 48 to 49 ORs in the species studied here, which compares to antennal transcriptomes of other tortricids, *Grapholita molesta* B., *Planotortrix octo* D., *P. excessana* W., *Ctenopseustis obliquana* W., *C. herana* F. & R.^21^,^22^,^23^. It is likely that more receptors are yet to be found, since other tortricids such as *Epiphyas postvitanna* and *C. pomonella* contain larger repertoires^28^,^40^.

Both phylogenetic and quantitative analysis confirmed that all three species possess receptors that cluster in the main pheromone receptor clade (Figs 1–4). We found six putative PRs in *Hedya*, ten in *C. fagiglandana* and seven in *C. nigricana*. Despite the fact that the PR-clade of lepidopterans is highly divergent^46^, it has been suggested that orthologs in closely related species might have a conserved ability to detect similar or the same pheromone components^23^.

The three species of our study use codlemone acetate as the main pheromone component, and the most highly expressed PR, which is expected to be tuned to the main pheromone^47^, is OR6 for the *Cydia* species. We have found that the corresponding ortholog in *C. pomonella*, CpomOR6, is tuned to codlemone acetate^48^. Considering the overall similarity of the OR6 homologs in the *Cydia* species evaluated (>90%) and their relatively high expression values (Fig. 3), we therefore hypothesize that this is a conserved receptor. Whether HnubOR6 is also tuned to codlemone acetate or towards another pheromone component is yet to be determined. Although its sequence is similar to the CpomOR6 splice variants (87.7–90.6 sequence identity), its expression level does not support its role in detecting the main pheromone component. However, our predicted HnubOR6 does not contain its complete ORF, which decreases its detectability and therefore its proper abundance estimation.

Apart from the OR6 homologs, we also predict a conserved function of the OR1 homologs. CpomOR1 is the most highly expressed PR on male antenna of codling moth and hence predicted to be dedicated to codlemone detection^28^. In the case of the beech moth, we predict CfagOR1 as a putative receptor for codlemone for two reasons: first, because CfagOR1 is closely related to CpomOR1 (79.9% amino acid identity, clustering with a bootstrap support of 85%; Fig. 1), and second because codlemone acts as a synergist for the main pheromone component of this species^43^,^49^. In the case of the green budworm moth, based on our phylogenetic analysis, we hypothesize a conserved ability to detect codlemone, through its receptor, HnubOR2.1, and to detect other codlemone isomers such as *Z,E*-codlemone with HnubOR2.2^49^. Finally, for the pea moth, the receptors CnigOR1, 2 and 5 cluster together with CpomOR1, hence we hypothesize may detect other compounds such as codlemone and codlemone aldehyde, (*E,E*)-8,10-dodecadienal, since these compounds, although not required for attraction or synergism, attract males to field traps^50^,^51^.

CpomOR3 has been shown to be tuned to pear ester, which is a codling moth kairomone, encoding host plant finding^26^. HnubOR3 as well as CfagOR3 are close orthologs of CpomOR3 (64.1 and 84.2% amino acid identity, clustering with bootstrap support >99%). Since these two species are attracted to pear ester^32^,^52^, HnubOR3 and CfagOR3 may accordingly also be tuned to pear ester or closely related chemicals. That OR3 is conserved in *H. nubiferana* (Tortricidae, Olethreutini), *C. fagiglandana* and *C. pomonella* (Tortricidae, Grapholitini), which are all associated with deciduous trees, but is lacking in *C. nigricana* (Tortricidae, Grapholitini), feeding on seeds of leguminous plants^18^, seemingly corroborates a role in host finding. Pear ester has, however, only been found in apple and pear^24^, which are hosts of *C. pomonella* and *H. nubiferana*, and its ecological significance in *C. fagiglandana* remains thus unclear.

In brief, our results point towards a putative conserved function of the receptors OR1 and OR6 for the species *H. nubiferana, C. fagiglandana, C. nigricana* and *C. pomonella*, for the detection of codlemone and codlemone acetate respectively. In addition, a conserved function of the OR3 homologs in *H. nubiferana, C. fagiglandana* and *C. pomonella* as a pear ester receptor is predicted. Molecular evolution estimates support these roles since purifying selection rather than positive selection appears to be the pattern of evolution of these receptors (Fig. 2). Considering Orco as a reference, it is clear that lower numbers of non-synonymous substitutions over synonymous substitutions are correlated to a conserved function, since Orco is the most highly conserved OR and its common function in insects as co-receptor is well established^53^. Therefore, our results support a conserved function among the analyzed orthologs of Cpom PRs. Furthermore, taking into account the difficulty of finding evidence of positive selection by analyzing the entire length of the gene, we also performed a codon-by-codon analysis for each one of these selected orthologous receptors and found yet again no sign of positive selection (Supplementary Table S1), supporting the hypothesis of conserved functions.

Other putative PRs may be tuned to compounds with similar functional groups similar to the ones detected by the codling moth homologs they cluster with, especially considering the high bootstrap support values and high degree of similarity between receptors within these clades. In the case of *Hedya*, the other main pheromone components (*Z*)- and (*E*)-8-dodecenyl acetate are likely detected with the PRs HnubOR8.1 and HnubOR8.2. Abundance estimation of HnubOR8.1 suggests its importance in detection of at least one of the three main pheromone components of *Hedya*. Furthermore, Porcel *et al*.^42^ were able to achieve partial mating disruption of this species with dispensers containing (*Z*)-8-tetradecenyl acetate, which further indicates the importance of acetate-dedicated PRs in *Hedya.* For the beech moth, other putative PRs such as CfagOR2.1 and 2, OR4, OR5.1 and 2, based on the phylogenetic closeness to CpomOR1 are perhaps able to detect codlemone-related alcohols^49^. Likewise other PRs closely related to CfagOR6 (OR7 and OR8) are hypothesized to be tuned to acetates, such as codlemone acetate isomers or minor acetates such as (*Z)-8-*dodecenyl acetate, which have shown behavioral and electrophysiological activity, respectively^43^,^49^. Regarding the pea moth PRs, CnigOR7, 8 and 9 may possibly detect geometric isomers of codlemone acetate, *E, Z, Z,E* and *Z,Z,* which are strong inhibitors of male attraction, playing a role in reproductive isolation^31^,^50^,^51^. Strikingly high percentages of sequence similarity between codling moth PRs and homologs of the three species studied, in addition to the high bootstrap support in our phylogenetic analysis indicate putative conserved functions as acetate receptors for PRs phylogenetically close to CpomOR6. PRs detecting acetates are expected in tortricids, since attraction of males of these species is tightly controlled by species-specific ratios of acetates, rather than alcohols^31^,^49^. However, due to the lack of functional studies in corresponding codling moth homologs, we did not perform molecular evolution rate analyses of these PRs and suggest to functionally characterize them before further hypothesize about their function.

To what degree PRs of different species are specific and sensitive towards common pheromone components still needs to be investigated since it is known that this subclade of receptors is normally under relaxed constraint allowing them to mutate and acquire new species-specific functions. This has been shown in tortricid species from New Zealand. While *C. obliquana* possess a PR (OR7) dedicated to the detection of its pheromone (*Z)*-8-tetradecenyl acetate, the orthologous receptor in its sibling species *C. herana* is able to detect the same compound in addition to the compound (*Z)*-7-tetradecenyl acetate, although these compounds are not part of its pheromone blend^22^. Similar results have been observed in heliothine moths, in which orthologous PRs of closely related species have shown overlapping responses towards common pheromone components in some cases and completely different specificity in others^37^.

Generally it is considered that non-pheromonal ORs are far more conserved than PRs. Accordingly, 34 cases of close homology between the studied species were observed (Fig. 2). So far, functional characterization of non-pheromonal ORs in codling moth has yielded ligands for only CpomOR19^54^. All three species in our study present a receptor orthologous to CpomOR19 and its amino acid sequence identity percentages range between 64.10% to 86.02%. Considering that conserved responsiveness was observed between *C. pomonella* and *Spodoptera littoralis* B. OR19 homologs, even when their sequence identity was 58%, we predict that the orthologs in these tortricids are probably tuned to indanones or structurally-related compounds. Although the ecological relevance of those ligands is still unknown, this case exemplifies the potential of functional characterization as guidance for future studies with closely related species.

Regarding gustation, we provide the first GR repertoires of these three species (Figs 5 and 6). Receptors from the three species were spread among putative clades tuned to CO_2_ (GR1, 2 and 3), sugars (GR4, 5 and 6) and bitter compounds (GR29, 30, 55, 58, 60, 68). The fact that we only included males and/or low expression might explain why we only found one single putative CO_2_ GR in *H. nubiferana* and *C. nigricana*, since for most moths at least three GRs are expected^55^. Only one single receptor was found for the “fructose” clade, CfagGR9. The relatively low number of GRs found in each species is expected since this family of receptors is much more abundant in other tissues such as tarsi and ovipositor. It is likely that by examining larvae and females, a larger repertoire of GRs will be identified, as in codling moth^28^. Moth species, such as *Bombyx mori* L. and *Plutella xylostella* L., contain on average 69 GRs^55^,^56^. However, the total number of GRs is seemingly related to the behavioral ecology of each species. A full GR repertoire of *Helicoverpa armigera* H., comprising 197 receptors has been reported^57^. Xu *et al*.^57^, showed that this taxa presents an expansion of the bitter receptor family (180 GRs), which has putatively contributed to the habitat adaptation and wide distribution of this polyphagous species. Our study does not allow us to draw conclusions based only on antennal GRs. However, it appears that both *Cydia* species share more orthologous receptors and higher expression of GR6 than in *Hedya,* which is probably related to phylogenetic closeness, rather than to their ecology. Further genomic approaches to unveil the full GR repertoires of these species might reveal differences related to their host range.

In the case of the IR gene family (Figs 7 and 8), the number of predicted antennal IRs range between 19 and 23, similar to what has been observed in *C. pomonella* and *Manduca sexta* L.^28^,^58^. However, it is likely that the total number of IRs per species might be higher. A genomic study in *Heliconius* butterflies has shown up to 31 IRs, demonstrating that lepidopterans might have larger sets of IR repertoires than expected^59^. Abundance estimation has shown that, as observed in codling moth, in the three species studied the most highly expressed transcripts correspond to the putative IR co-receptors IR8a, IR25a and IR76b^12^, in addition to IR21a, which along with IR25a, seems to be associated to the detection of small changes of temperature in *D. melanogaster*^60^,^61^. Another IR conserved between species is IR41a.1, homologous to IR41a of *Drosophila,* which along with IR64a and IR76b are reportedly involved in amine sensing^62^,^63^. Recently, it has been demonstrated that IRs 25a, 93a and 40a of *Drosophila melanogaster* are required for humidity sensing^64^. In the studied tortricids we identified IR25a and IR93a homologs, but none for IR40a. However, it is difficult to speculate about putative functions of predicted IRs since most work on this family or receptors has been done in the fruit fly and an efficient *in vivo* system for heterologous expression of lepidopteran IRs is not yet at hand.

We have carried out a comprehensive analysis of antennal transcriptomes of males from three phylogenetically related species of the family Tortricidae. For the first time, we provide gene sets for putative chemosensory proteins, including pheromone associated proteins allowing us to hypothesize conserved functions based on what we have observed in the functional characterization of receptors of *Cydia pomonella.* Our results indicate that closely related species might conserve sets of PRs, allowing them to detect not only their main pheromone components but also similar pheromones, enabling species recognition and avoidance of interspecific mating attempts. In addition, homologous and divergent ORs, IRs and GRs provide insights into the ecology and phylogenetic development of these species. These results are also starting points for de-orphanization of receptors. This know-how will greatly facilitate the further development of insect control with semiochemicals.

## Methods

### Insect collection and RNA extraction

Male adults of green budworm moth (*H. nubiferana*), beech moth *(C. fagiglandana*) and pea moth *(C. nigricana*) were field collected in traps containing synthetic pheromone blends^31^. Traps with collected adults were taken to the laboratory and kept alive until antennal dissection.

Total RNA was extracted from the antenna of 100–150 individuals per species following Trizol-based extraction protocol and spin column purification with the RNeasy Mini Kit (Qiagen, Venlo, The Netherlands), as previously described^28^. Total RNA was quantified with a Nanodrop 1000 spectrophotometer (Thermo Fisher Scientific, Waltham, MA, USA).

### RNA Sequencing

Total RNA extracted from each species was sent to the National Genomics Infrastructure sequencing facility (Uppsala, Sweden). RNA libraries for sequencing were prepared using TruSeq Stranded mRNA Sample prep kit with 96 dual indexes (Illumina, CA, USA) according with manufacturer’s recommended instructions with the following changes: the protocols were automated by using an Agilent NGS workstation (Agilent. CA. USA) with purification steps^65^,^66^. Samples were clustered using cBot and sequenced on a HiSeq2500 (HiSeq Control Software 2.2.8/RTA 1.18.61) with a 2 × 126 setup in RapidHighOutput mode. Bcl to Fastq conversion was performed using bcl2Fastq v1.8.3 from the CASAVA software. Quality scale was Sanger/phred33/Illumina 1.8 + .

For each species two fq files were produced, one containing all left-pair reads and another containing all right-pair reads. All sequence read files were deposited in our private project account on the UPPMAX server (Uppsala, Sweden).

### Bioinformatic pipelines, phylogenetic analyses and gene annotation

Our methodology followed the protocols described in Walker *et al*.^16^. Briefly, previously produced.fq files were subjected to quality control and the reads in which the sequencing adapter information was present were removed. To do this, the software Trimmomatic version 0.32^67^, checked and discarded from the 3′ terminal nucleotide and moving in the 5′direction, each base having a PHRED score lower than 20 until a base with a PHRED score greater or equal to 20 was encountered. From each species, processed reads were then assembled, *de novo*, into one transcriptome using the software Trinity version r2014717^68^. The software cd-hit-est, version 4.5.4–2011–03–07^69^, was used to identify and remove redundant sequences that share 98% or greater identity with other sequences. The assembled transcriptomes were then queried with the most updated ORs, GRs and IRs sequences of *Cydia pomonella*^28^. Blast version 2.2.29 + was used to perform a blastn query and a minimum e-score threshold of 1e-05 was required to be considered as a hit. Top blast hit transcript clusters were extracted from the transcriptome file with an in-house command line script. Relevant transcript sequences were translated into protein sequences with the ExPASY web Translate tool^70^. Translated sequences with open reading fragments (ORFs) shorter than 50% of the average length (ORs and GRs = 214 amino acids, IR = 337 amino acids) were excluded from analysis. Sequences were aligned to chemoreceptors from *C. pomonella*^28^ and all new putative ORs, GRs and IRs from these species were named according to the closest homolog in *C. pomonella*.

To compare the phylogenetic relationships of predicted chemoreceptors, different published chemoreceptor sequences from different species were used. For ORs and GRs, olfactory and gustatory repertoires from *E. postvittana*^40^ and *C. pomonella*^28^ were compared to the predicted from *H. nubiferana, C. fagiglandana* and *C. nigricana*. For IRs, reported candidate IRs from *Grapholita molesta* were also included for comparison^21^. For each gene family, all amino acid sequences were aligned using MAFFT online version 7.220^71^, with the FFT-NS-i iterative refinement method, with JTT200 scoring matrix, and default parameters. Aligned sequences were used to calculate the best fitting model for comparison in MEGA6 software^72^. Then, a Maximum Likelihood Tree was constructed using the JTT + F + G model with bootstrap support inferred from 600 replicates.

Additionally, to estimate the balance of negative and positive selection between selected homologs of codling moth PRs (OR1, OR3 and OR6) and Orco, the dN/dS ratio was calculated. To do this, analyses were conducted using the Pamilo-Bianchi-Li method^73^. Tests of positive and negative selection were carried out for the overall average among homologs and for the pairwise comparisons with the corresponding OR of *Cydia pomonella*. Furthermore, to detect specific sites under putative positive selection a codon-by-codon analysis using the HyPhy software Package^74^ was carried out using the Tamura-Nei^75^ model and default parameters in MEGA6^72^.

### Quantitative analyses of gene expression

To estimate the expression of detected transcripts in each species, the RSEM software package, version 1.2.12^76^, including Bowtie version 0.12.6^77^ and Samtools version 0.1.19^78^ was used, allowing measurement of transcript abundance estimates as fragments per kilobase of transcript per million mapped reads (FPKM). To classify the identity of the most highly expressed transcripts, the top 50 most expressed sequences were manually extracted from the assembled transcriptome of each species and a blastn query was performed for each one. To represent abundance estimation of each predicted receptor, heatmap plots were produced as described in Walker *et al*.^16^.

### cDNA and expression via RT-PCR

To qualitatively corroborate the differences in transcript abundance estimated with RSEM for putative PRs of each species, cDNA was synthesized from the same samples of RNA (at 250 ng/μl) sent for sequencing. To produce cDNA, 3.5 μg of antennal RNA of each species was used as input, using the RevertAid H Minus First Strand cDNA synthesis kit (Life Technologies, CA, USA) according to manufacturers recommendations. RT-PCR assays were performed with 1 μl of cDNA, 0.25 μl Dream Taq Polymerase (Life Technologies, CA, USA), 2.5 μl of Dream Taq Buffer, 0.5 μl of 10 mM dNTPs, 18.75 μl of deionized water and 1 μl of each of 10 mM gene specific forward and reverse primers (Supplementary Data 5). As negative control, no cDNA but 1 μl of deionized water was added instead. For all putative PRs of each species, PCRs were programmed as follows: Initialization 94 °C 2 min; Amplification 31 cycles of 94 °C 30 s, 55 °C 30 s, 72 °C; Final Extension 72 °C 10 min. PCR products were visualized on a 1.5% agarose gel after 1 hour of standard electrophoresis at 100 V and 15 min of staining with standard application Gel Red (Biotium Inc, CA, USA). For each gene, technical replicates were performed in duplicates to verify consistency of amplification.

## Additional Information

**Accession codes**: Transcriptome raw reads sequence data are available through the NCBI Sequence Read Archive (Accession Numbers: SRX1741573, SRX1957902 and SRX1940816). Chemosensory Receptor sequences identified are available through NCBI: *Hedya nubiferana* ORs (KY283585-KY283633), GRs (KY283551-KY283561) and IRs (KY283562-KY283584); *Cydia fagiglandana* ORs (KY283634-KY283682), GRs (KY283683-KY283694) and IRs (KY283695-KY283713); and *C. nigricana* ORs (KY283714-KY283761), GRs (KY283762-KY283772) and IRs (KY355445-KY325463). All coding protein sequences are available as part of the Supporting Materials.

**How to cite this article:** Gonzalez, F. *et al*. Antennal transcriptomes of three tortricid moths reveal putative conserved chemosensory receptors for social and habitat olfactory cues. *Sci. Rep.* **7**, 41829; doi: 10.1038/srep41829 (2017).

**Publisher's note:** Springer Nature remains neutral with regard to jurisdictional claims in published maps and institutional affiliations.

## Supplementary Material

Supplementary Figure and Data

Supplementary Dataset 1

Supplementary Dataset 2

## Figures and Tables

**Table 1:** Transcriptomes assembly summary.

Species	Paired reads	Unpaired reads	Contig gene clusters	Transcript sequences	Average contig length (nt)	N50 length (nt)
*Hedya nubiferana*	70,320,761	3,565,915	107,543	137,391	650.61	987
*Cydia fagiglandana*	77,619,812	2,590,143	100, 279	135,453	611.89	860
*Cydia nigricana*	87,531,484	3,520,979	137,083	151,491	540.18	697
