# Supplementary material for: Antennal transcriptomes of three tortricid moths reveal putative conserved chemosensory receptors for social and habitat olfactory cues
Source: Sci Rep. 2017 Feb 2;7:41829. doi: 10.1038/srep41829 (PMC5288797; doi:10.1038/srep41829)
Supplement: Supplementary Figure and Data [file srep41829-s1.docx]

Antennal transcriptomes of three tortricid moths reveal putative conserved chemosensory receptors for social and habitat olfactory cues

Francisco Gonzalez, Peter Witzgall and William B. Walker

SUPPLEMENTARY FIGURE

**Supplementary Figure S1.** Putative function of 50 most highly expressed proteins in male antennae of three tortricid moths. Numbers depict percentage share of summed FPKM values for representative genes of each functional category in the most abundant genes relative to the summed FPKM of all of the fifty most abundant genes. Functional categories informed by Uniprot database.

**
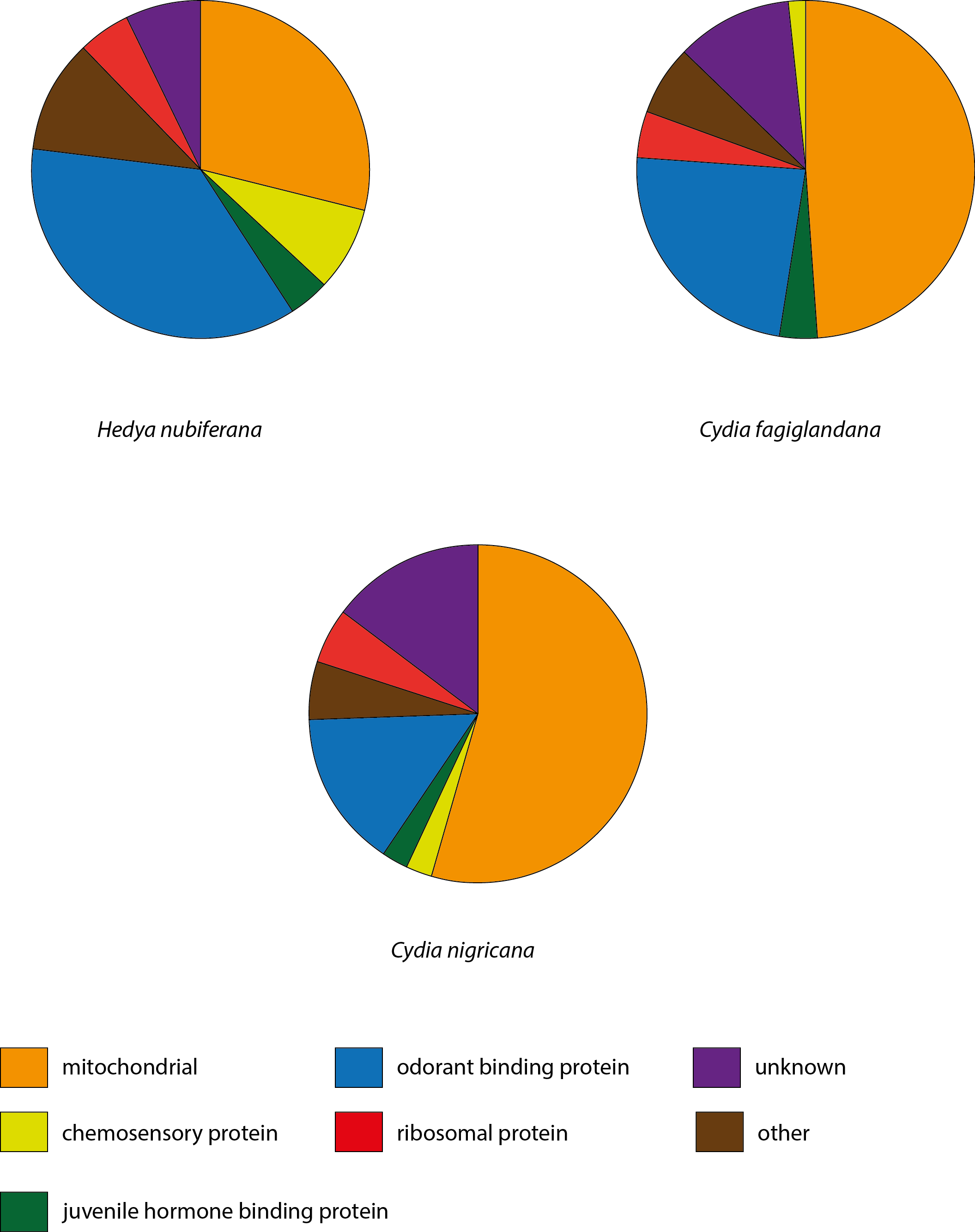
**

SUPPLEMENTARY DATA

**Supplementary Data S1**. Protein Fasta Sequences of chemoreceptors from three tortricid moths.

1. Hedya nubiferana

>HnubOR2.1

MYKTKGLEYLKKEFCDDSDRPSPMVFRHVKRLIFIMSTIGSWPHNISGRPRLHNFLSTYSFILIIVAVMFSGLAGGYLKQHWDEMTFFDMGHIILTICLNGIYMTRLMQARAKKVSEAIRDCFLEFHLYYYKDRSAYWTKIFSEIHIISGIFTIYILILMMVGMSLFTLKPLYTNYSKGMFSSNPPPNGTFEHSVYFYFPTDVFYTTLKGHWILFAINLPNNYNVTCGVFGFDLLLILAVLQIYGHIKIMKHNLLSIPLPKKESDIMFSAEENRQVAQLLKDIIIHHNLIIKFVDKFSDAFQEYLFLFYFFLQIISCVLLMEVSTLNPEALAMYGPLTLGMFQQLMLLSIMFEILNSKSEELEQVVYAIPWEFMDGRNRKSVLFMLYKVQTPISLKAGGMVPVGVKTMSSILRTSLSYYMVLMALAREQ*

>HnubOR2.2

MTAEWIERFKIFLNDAEGIVNPLHYRYMKQTKILMTEVASWPYKVFGDMKSDARISKYNIALILLGICLPFLSGGHLYTHADTMNFNDMGNSIIIIFLDFIFLQRLVLARTDKFFNLMKLFINKFHLIYFKNRSAYAEKVFHRIHLISGVFTMYIFVLLHFGILVYNLIPVYGNYKNGMFGSNRPTNSTFGHSAYYYVPKDYFYITEAGYVILAIINLYLSLIIAGMLTNFDLVVYLIVFQIWGHLKVLKHDLYSFPRPKVRENDVDNGMYTPEENFYIRKLLSDNIEHHQLIIKFVDECSYVLGEYLFMFYANIQVISCLLLLEMASMTAGVIAKFAALFLAIFISLIQCSVLFELVNTKSMELENAVYQLPWECMDVNNRKTVIFFLYRCQTPLSIKAGKIIPVGVTTMLAVLKTSCSYFMLLQTLTKEV*

>HnubOR3

MFFERNSHSEITSPKDLGYIKQVALSLNRIASWPLTKSKANKKYTFSVKRNIAYILFEAILLFLQATYVRNYKHTLSFFKMGHTYITSAMTVICLQRLTMPWLNQYREVAREFLEKIHLYNHKNKSDYAMKDVYSRVEKICIFFTLFIHYQMYTGILLFNITPWYKNLKAGMFSSNKPSNGTFEHAMYLELPFDYLTNFTGYIFVFILGWVETYTVASTFCLGDLYLSLIVFHIWGNLKILKHSLESFPRINNQRMTKIERSWYTKVESEHISLLIKEIVNHHRLIMDFMSKTSNTFSFFLCIYFAFHQIIGCILLLECSKLDSEALGKYGPLTVLLFQQLIQISVIFELIGSQSETLIDSVYGLPWECMGLKNRKLVLFFLQNVREPISLKACGMVSIGVQTMAAIIKASCSYFIILRTFTSNEEMTSG*

>HnubOR6

MFASELPENKSFVHSVDYLLPFDAYRSFTGYLVVFTWNWFPTYNIPTAMGIYDLLVFVMVFHMVGHMNILYNSLKEFPRPKEGQSEDPLPSTREYNEEIFGQLKNVIRHYQMIKEFMGDMTAAFDLTLCCYLAFHQVMCCLMLLECSTLEPEALVKYGLLAAVIFQQLIQTSVAFELIKSKSDSLGDEVYAVPWEYMDVKNRRILLLFLRNVQDPLGLKACGMVPVGVLTMSTIIRTSFSYYLMLATFD

>HnubOR8.1

MKNKNWVDFVTVYRKKHDSDTKDVYDLDYMNAFRLMMDAIGQYPDKEFGVVNRRTRILRAYRYVVMAVTFMCETLCITYAWKNRKTLDFFILGHLYGNIMTTAVLIQRSTLPYQKSFRSLLQRFISKYHLIHLEYESDFVKQEHIRISKFCRIVTMILSLQIFCGVATFNLVPLYNNYRAGMFSDNKPENATLDHAVFFELPFDYTTEVSAGYFAVVVFNFYPCFNVSCGLCAYDGLINILVFHVWGHFNIIVHKLRTFPKLSLEDFQQQDLVQEFNKQAFLKLKNIIQHYQFLKEFMSETSAAFETTLCAYLSFHQLNGCVLLLECSALTAAALGKYLVLTIVVFEQLIQTCMIFEQINSKSERLADQVYDLPWEQMDVKNRKIVLVLLRNVQNTLSVKALGVLPVGVMTMSAIIRTTVSYFVLLNTIAK*

>HnubOR8.2

MEDSVKDVFELGYMRMIRFSLSTIAQWPNKNFGTKTRAARIQSGYHYILVTIIVVGECSCALYLWRNRKILDFFNIGHVYGNILMTALLIQRITLNFQNKYCSVLKKFASEFHLMHHAHKSDFAAKEHSRINRFCKIVTIVNHVQVACGLPMYLGVPHFNNIRAGMFSENRPANKTFEQSVYYELPFDCTTDVTGYYIMFFYNWYPAYNVCAAMCIIDVTIFVIVFHILGHLNVLMHNMKYFPRPSDENEQSEREYNEQAFGLLKDLVQHYQIIKEFMSDTSSAFDMTLFVYLAFHQLNGCVLLLELSSMKAEAIIKYGLLTFFTFQQLIQTSVIFEQISSKSEQLAHKVYGLPWELMDTKNRKIVLLLFTDVQKKLALKVCSIVPVGVTTMSAIIKTTISYFMLLNTIAK*

>HnubOR10

MIVQKIISFSKILEDPKHPLLGPNLKGLYVYGLWQSGSKFRNSCYNFIHFCAFLFVISQLIELWVIRRNLLEALHNLSLTALGMVCIFKAVSYVIWQSDWKELVEGISAEEITQKDKLNDTCAKLKQNYTNYARIVTYLYWNVVVSTNVTMVSAPFLKYATTSEYREQISNGTEPLPQIFSSWFPFDKTTMPGYSVAIFVHILINIHGGGVIALYDSNAVAVMVFIKGQLAILREKCQNLFDDYGELDRQITLDRIKECHRHHNFILRHSTLFNSLLSPVMFLYVLVCSGMICCSVVQFTSDEATAAQKIWVLQYTTALVSQLFLYCWHSNEVVVECQHVDGGVYNSEWWKGDVRIRKQLAMLGGKLTHGIAFSAGPFTTLCLPTFIDVGSKTKYYLVTKIKDKKKPNALCYG*

>HnubOR12

MSLKQSDSFNQNRLVWTIFGLWPGKIPYKYYKYFSCTYLIITLVAYNILLTLNLYYTPRKIDFLIREVIFSFTEIAVASKVSMVLFNRKKLDAIFGLLDCDEFQGSNEIGKEIVQKHNKYYKTYLMIYTVLSNFTYLSQVLFPVIGFIIFGNTLDLPICKYYFLSDETRNDYFTLIFIYQSFGMYGHMMYNVNIDTMIAGFMVLAIGQVKVLCHDLENLKTEKSHRGDDGMLDFKQQQKLRKILNHYALLLEYCEAFQEVISGTMFVQFGIGSGIICVIMCGLLLPSTVETFMFMVGYFVAMNLQIFVPAWLGTQLSYESQELMVAAYKSEWLPRSERYKTSIKLFVERAKHPVVITGLKIFPLSLATYISIMKTAYSCFALLRIIQDRQEQAVH*

>HnubOR13

MRPLRQIDCFKVNMKFWKLLAIWPCWTEMHPYYRYYVYFFISFFVILNNILVTVNFYYLPRQLDKFIEEMIFYFTELAVTSKVLTFLFMHEKIVKILNTLESDMFQPDSHTGLKIVEKAKKFNVRYWIIVAVVSATSHLTQILSPLLLHFFLHMKLDLPLCSYSFLSEDTKQYFIYPLYWYQAYSMHALVLYNINIDTFILGVLIFALAQLDVLDDKLRRVTDRQKVALTDGSIDVAPEDDSMMKLKDSIIHYDELGKFCDLIQEVFSTTLFVQFSVASCIICVVLFRFTLPAPWQYFIFLGSYMFIMIIQILVPCWFGTRIQDKSQLLSEAVYSCDWSAKTRYFKSSLRLFVERANKPLSITAGKMFPLSLTTFTSIINSSYSFFTLLRHMQSRQ*

>HnubOR14

MSSGSGDSAMELVPASDDVEFKPFRETYKIITFTMIVGMLYPTPHTAHCRLVGIVVVLVSVIPLATMTILDVWSSWLRGDIINIIRHFTVIGPFLGALFKMILFFISREEAWGIIRKIDHDHARYNTLPESHKAIARSHIQNTQYYSEKCWSITVATCVLTFPLTAVFLTWYNFTFKEDPVKYMIHDMNKPFSAPEDRFKSPYFEIMFFYMAYCSTIYIISFTGFDAFFGITINHACMKMDMACKIMDDAMTERDREFRRRRMMDVVTEQNDVFRMVELIQETFNNWLGLIVAATMLQICNCMYQIIEGYGIDPRYLIFIFGTVAHIYLPCRYAAKLQGSALEVATRLYCCGWERVNDPRARNMLIFMIARAQYPMKITAFNMFDFDMELFVSILQTSYSMFTLLRS*

>HnubOR15

MFPRVSLPLIIQIRPMAERRINKSIDVKSILREHYRVLIECIAQHQAILNFTKLVENTYHSYLLFQLSGSIGLICMTALRIIVVEWKSVQFMSMVMYLSVMISQLLVCCWSGHELTATSLELHTVLYECCWYEQDVRFKRALLFTMMRVGRPMEFRAGRYVTLSRQTFVAILRMSYSYFAVLKQTNSRNEVLEHDKLDFNKLSVQNDNDLLFQ

>HnubOR16

MAKPKLNIGDLYLSRAKLIMSLLGVWMPPKNESVLRKLYKFFMLFLQYIFLLFQVIYMSQVLGDLEEVSQSSFLLFTQACLCFKITVFHVNINSFRELLGQMNSNVFMPQNQRHEKILKLQAAKIKRLLLGFMVSSQTTCSMFALRPLFDDENRNFPFKMWMPVSPDHSPQYEFGFLFQLLTISMSAFMYFGVDSVCLSMVVFGCAEIDIIKEKLMTIRPMAERR

>HnubOR18

MLEDNVTIFLNRPRNILFYLGIWLRPAIHVTLYVIYAVTVMLSQYSFVLFEFIYIAQAWGDMDAVTEASFLLFTQASVCYKVTRFLINKKNLVVLLDLMEKEVFQPQNKKHERFLLDQSGMVKRLCLFFLCSALTTCTLWGLMPIVDSTEGQRIFPFLIWMPVGPNESPQYELGYFYQMMAIYISAFLFIAVDSVALSMIMFGCAQLEIIMDKVEQIQPVPMSGKLKKEERDLRIEDNYALFVRCLKHHQAVIRFIETVEDTYHANIFFQLSGSVAIICIIGLRITATTPGSVQFISMLNYMVTMLSQLFLYCWCGNELTIRSEILREVMYLSPWHEQSNRFRRLLWIAMERMKRPIIFKAGHYIPLSRPTFVAILRSSYSYFAVINQTRNKEK*

>HnubOR19

MTKMFLVLANRKLVKKLNSEMVEICKDHEDEDRAAHNYRVIRLNVIVYVLTIYAAWFGFLVEGIRKVLTGSHFITTVTYWPFYEDDSMTAVSARIFATLVFWVLMSTMVSTDSMCLVTLIVYKYKLVTLRYYLENLRAQFDKNNHKGNEQYASDQLYAGLVEGLVMHVNLLRLSKDFDRSYGSILAFQVLLSSGSTVSLLLQLALPKDLSVVLAVKIVLFIISIFLLLGLFLCNAGEITYQASLLSDPVFYSGWHATPMRKDLRQLALLACASAQRPIVMKAFKMLELTYGTFIQIVRCTYSVFALFYAQNE

>HnubOR20

MVHEMELKYLEDKQYEFPYFQLVFAYYTFGIFVLVPNFAGFDGSFCIATSHLRLKIKLMTHSVQSAFKDSKNISELKTKLKNCVKDHQEALEFYALIQRLYGTWLFAVFLLTSFLISCNLYQIYLTGVDPRYTMFALTGVFHMYTPCYFASCLIELGEQAGTDIYCADWESWADPAVTKFLIFIIARAQKRLLLNGLGIVTFNMESFVSLMQTSYSFFTLITSK

>HnubOR22

MAENKDSIKVFSDIVTKTNNKKDVKSEYYLLTPRQQLFYQKLSFAMTVFKMGKREWWGFSPIHNFFIWNSYLIFIVCPICLTLQVVYLYTNYNDLSFRTLGTMFSIIPATAAVVIKVLICMIPAYPQIMKQLMDKIHLNNYLDIKDEFLKKKLIQVERHTRWITVCLSAFILFDWLLWIFVPLMNNIKNKELIENGTVRMETCLYMWMPFDYGFDYNTWAITHALNVYLVGSGCCIFALFDSINFIFIFHFLSHIDILKYNITTYFSTEIDDSETKERIVSVIKYHSFILSTFKDMQAAFGMNVAVNYAHNLIVDSLLLYQIMVGDKSNRLTYVIMMQFHMGCLILLSLALEQIRIKTEDLPLVLYNIPWENMSTSNQKLLVPILLRMQTALVFKAAGGLAAGVHPLASVSNLGDKLSS*

>HnubOR25

MFGSLLELSDNFFAFNLKYLFLVGLWPSDPWATSHPILYSMYETCIHVLLITFLILSGMANYEIKHDVLMLMTNLDKCLVAYNMFAKISMFVLKRKQLEILVSEIIGSGDQITAERKKLMKMIVLVMTGLSMAIVSSFSALAQYHNEMTVQIWMPFDPKKSRMNLILAGQVVAVLYVVPVILRGVAMQGIVCSLIIYLYDQLIELQRRIKSLEFTVETEGAMREEFKNIIKKHIRLMGYSQDMNNIFGEYFLVQNLVVTLELCLNALMATMIGFEQKTLLVTFFAFLCVALLNEYIYCYLGNEMITQSDNLALAAYESSWTSWPLDLQKDLLILLGVAQKPLQLSAGGIVAMSIQTYGQILYNGYSIFAVLNDVVG*

>HnubOR26

MSENEGQFLSYLGITPHLKVLRGCGILPVDPSNSKLKRRLHVFYMWIGFMILMLYTLLQIINVYQISADINKVIDSLFLLLTFFACICKQIMFLLKPHKIQEIFDIMKGSSFNQGLDAHRPLLVNTNYYAALLLRIFNRLCVAFCFLWSLMPTISHLNNKTVELTIWVPFDANKRSIFLIATGYTWLQNTWCGFNISTMNLFIVYLFTQTKTQLSILRINLESLVSVCQEEARISSVSFTEVLEYRFKGIIRHFNDIVKFSDLTEDIFNGALLFQFLFSGGIICTTTYRTTYVNESCFGRVFINDDVHDLHSFGIILILLFGKRGNIRE

>HnubOR27

MLKKYVSRLEDPNHPLLGPTLWGLQSWGMWQPNTGPSRIIYNLIHLAAILFVLSQYFELWLIRTDLELALRNLSVTMLSSVCVVKAGTFVVWQEYWRDVVQFVSTLEKGQLKKKDKITPTIIDGYTKYSRSVTYFYWSLVTVTVFTVILAPLGVYLSSSEEQHELMRNGTIPYPEIMSSWVPFDKTKGFGYWLVILEHSMICFYGGGIVANYDSNTVALMSFFCGQLELLVANCKRLFGENNELVSYSEAMERIKQCHAHHLSLIKYSRILNSLLSPVMFLYVIICSLMICASAILLTQEGTTTMQRMWVAEYLVALIAQLFLYCWHSNEVYFMSEKVDKGVYESEWWQCRVSLRRCVILLGVQLRKTIVFEAGPFTTLTVATFIGILKGSYSYYTLLSSNED*

>HnubOR28

MSAQRVGATRAGSSVASLLRTLRFCGFCRLTPLPPGKNPPCITTRIKRVMHDAYCGFALAVTSTYLAQELIYAYQERSDLDKLARVLFLLLCHVTSIAKQVVFMTRASRVAELVKGLDDVTYNPDEAKRASLLTQRAQGASRLGKAYASTAALTCALWTIFPLLVRLGGARVTFALWIPFAYHSWPEFLVVLMYTHYVTSLVGIANTTMDAFIATVLGQCKTQLAILKMDFESLAERASERAHETGERLGAAASTLLVRCIKHHHKICDTSRELQSVFGSAVLLQFGIGGWILCMAAYKLVGLSVASLEFVSMLMFLMCILTELFLYCYYGNEVAVESAQVSEAVYGMQWVGPDGVGRDVQRALPLVLCCC

>HnubOR29

MINAFLKSLEDPDRPLFGPNYWLLNKIGLILPENSTQRLFAIIMHEVATYFVISQYMELFVIRSDLDLILTNLKISMLSTICVIKANTFVFWQNDWRQVINYITEADNFERENQDLAKGWIINSYTKYCRRVTYFYWGLVFTTSVTVTTAPLMKYYSSSKFREGFRNGTEAFPHIFSSWMPIDKEHSPGCWITVLWHIGICAYGATVAAAYDTCVMVLLVYFRGKLELLRIRCREMLGTCGQGISDEDTISAVRQLHQIHVLLLKHSRLFNSLLSPVMFFYVVMCSLMICASAFQLTSATDTTQKLLMAEYLIFAIAQLFMFCWHSNDVIYKSEEVMLGPYESEWWAAGLRQRKSILLLQGQMQIVHIYTAGPFTNLTLSTFVAILKGAYSYYTLLRK*

>HnubOR30

MTKFIYENLKFETKFWIATMAMRLNRSHPYVPRDKQWRIQFGAILMVSAACFIFLLYSILAHDIQNGDFADASKNVIMVIVAYTITYKYSILLRYQESVTELIRIVDEDYELAKEFCEEEQRIVSHYAEKGVKVCQYWFVSACSTSAIFPLKAFLLMGKSYMAGEFKLVPMFELTYPGILEDYKNVHVIFCMLFCMTLFFDFYATSMYVGFDPIVPIFMLHLCGQIDILNLRISKLFSDPKVPDEVVRQNLRKIIVKLQDIYEFVEVIKTNFTVLYEFNMKTTTFLLPLNAFQITESLRNGEINLEFMGFFSAVILHFYMPCYYSDLLMETGENFRLAIYSCGWEKHSDQRILRSILFMLTRAIKPLVISTIFCAICLDTFAEMCRHSYSIFNLMNAAWA*

>HnubOR32

MKHKYSLFLARYLPVLNTENSSLVNTVQRLVRNTGLWSQQSLGLHWLAKFAIICFLSTNLTQVATLIIDRDDPTRMFECFSVLSFCGMGALKLFNLYANRERWLFILSKVKQLENEQLHGITEVSFTQEYDEKEVATRYIEKYTNRHTSIASLLLKLYSITAIIFIVTPFVEYVIAAKHEANPHILPGWAPLDKVGIIGYLINLTFEAVGAVYCVFVHVAFDCTSVGIMIFICGQFSMLRHTTENIAGSGRDCKPSVARDTRAHFRIIKNHGTHITLCTVINELDSTLRSILGVYFLVATLTVCSVAVRLNSETLSAMQLASLLQYMCGTLTQLFFFCRYGDAVFHESSFDMGAGPFGAAHYRRASYKCVDMRYDS

>HnubOR35

MPTGENKRIRNKKTFRFSFQNILSCLEDPDHPSAGPFLRFMNLTGNWHPHMELKLTRFKQVVYYITMFFFFSLYFKCVISLKLSALLYILQTAPFHMGTPKTIFFRKNYNEWEKLVGYMSTTERQQIADKDDEVLDIMDSYTQKSRKVNYLFWLLAFLCNISIFTEPYLKNTIVENGTDVYVKLFDIYVPFDQDVPPGYYYSMALQTVLGNIMSSYVVSWDSLVISALIFFAGQLKISRVYCSKMVDPQSRAKTHQNIVHCHRFHTSLVKHQKLFETLISTVMFMYLIVISINLGACIIQISESTEDIPALMASVLFIIGCLTQLLIFYWFSNQVTEESLSVSYGIFESDWATMDAEVLKEVSLMQYTTSKRLAFRAGPCNEMSLSTFVGILKTSYSFFTLLNETN*

>HnubOR37

MECFGSIQEAVRRVRLRLRADTFDGLVWLVNITPSIAGFDITRKRIAKPFWILHLSLLTYVYGVGSFVYQAKHAEVASDFIKSFVNVSILVLTANNSYWWLMQRDLLQKVLRKAKESDNCTINAGLCVDKHKRLLYLIKCILISFYGFNLGNEFTTYLPKRADLNESTFSMTPCVGIEPLTTSPKREICIALVSIQELTIVIVVLNFQTTMVLLIAHTSAMYLLLSDEIITLNTLLTDPSKYDLVKERLADILYRHALTLDITSDLRALYNMPMGINFGSNAVCICFFFFLSLEEYLNFMPIMTYCFFVFFLYCFLGQRLTNAAEVFSCAVYSCGWEMMNMKEQRAIFTMLVQSQKEVDLLAADLIPVNMRTFASTCQAIYKFTTVVKL*

>HnubOR39

MLALSQEMRELWSDAQLHYKENFNIDTKNNQDKSANRELNKYVKERLYCIVQSHAMNVDLVKKVNDIFRNAIATEFLLLTGALVADLLAGLEQTYLILPFAVMQVSMDCYVGQKLMDASSVFEGAVYDCKWENFDASNMKTVLMILKMSQKTLKLTAGGVSTLSFGNLMVLIKSVYSAYAALRSTME

>HnubOR40

MGERFDVTYKVCSTALKLFGAHPSSSLSAKWLILTFMSYIVNICVFISLIYSSFYESFTKVCANMALAVVFVIISFDHAILIMHKEKFSDLIEFTEYDLERSEAFCPEDKMIVLKYNKRSKKASKIWYVASIITLASFLIEPLGYTIYSAVIGHFEPMPIFEVTYPDYIEKITNRIDIYLVIYILQSYYVWFSCVVYTGFYSLGIILILHTCGQIELVIRHLGQLFVKEHIDVTVTQKSIRGIALHIQNIYRFVDQIQYTHKTSYEICLKTAAISIPITCFGVIESQENGSISIELIIHVFGFLGQCYIPCYYCDLLLRKV

>HnubOR41

MGVKFDNTFKLSVAALNLNRGHPSLPLNLKWAAQMFLLYVGFSPNFLGFLYSSIFHDDFPKICRNLSEALVFNIICINNAILIVYKKKINDMIKITEENIESSKKLSSEDELVVKEYTEKGNKAAKVWLAVCIIAGGVLPGKAIVGSSYLSLQGNLTLVPIYDVTWPAYVDDRKNEFYMYLIIFGFQSFYIICICLMFVAFYPLGPIFMLQACGQIELVIRKLGRLFKDDNIDSDETVKSLQDIARHVQDIYRYVSIRNKKFNLYVFTTCLGCTRTVILFLSFVDIIQKTHTALYEMCLKTTTFLIPLSLIGFVESYNQGNANIQLICFVCASGMQCYIPCYYCELLATKGEELRYAIYGCGWERHWVPKTRSTILVMLTRTAHPLGINAVFCMVGMAAFANVCNLAYSIFNVMNAAWN*

>HnubOR42

MIATTRSSTITTRKRPTIMGTPTFQDVFKQIRANFSLMGVPEDKTKISVKFYLMAMSLTLILVEEASFFVSKMAPENFLELTGLAPCLCVGILSILKIVPVAMKRVTVFSLAERLEKLSAEILNDPMQTDVVRHDISLLQTLIKYYFVLNAVLISVYNFSTPLYILYHYIMTKEEIFNLPYAIIVPFSTERWTTWTLVYVHSIISGFICVLFFTTVDALYFILTSYVCNIFAVLSEEIKNLEKPTYDTLVKIVKKHLTALELADDLEAIFTLPNFFNVVVGSIEICALGFNLMIGDWSNVPGCMLFIFSVLFQLFVMSVFGENLIRASTEIGESVYACDWYKMNRQSKKLLLLVMTRSRVPKRLTAFKYSVICYEGFTAMISTSWSYFTILRTVYSPEEQ*

>HnubOR44

MKFLREYMKRLSFLSRFLPYGVLDSWEDLNPRLYHGVHIYWLKFYGLWYNMHPKSSILFWIHLLYAIAVLWLVCFMPGIGEVVYLLKRRDNIGEIAEGLYLFLSEMYTYIKLSAFWLKRKEIMGLLEYLHRDEFKAKEVEHREILKKSINTARFVMTYYSTMCVGAVSVGILMPLAEQFEVLPTNVEYPYFDVYKSPAYEIIYIHHIYYKPATCIIDGVMDTILAAFIASAIGQINVLSFNLKNFNLLAERRRQKLPYTSMNMNINIMNMNFEESTRYCVRAVFKDIIKHHNSILRYVSQIERAFSLASALQLMLSAMVLCLVGIQFLSIEEPSSHPIQIAWMAIYLTCMLIEVFIICWFGDELIWKSWELRQAAFEAPWPTTDPKTAMFIIIFMERCKRPLRVTAGKIFTLSLDTYTNLINWAYKAFAVMRKMKK*

>HnubOR47

MPDTVDPNRPRRYFSVHYRLLRFLGLGWWHHPDEDDFRNFPGWYLYYSILTQVVWVAGFVGLETIDPFIGEKDIDRFMFSLSFVITHDLTCIKLYLFFFKNRAIQEIVRTLEIEVYDYYQNIDKNRATIRITRIMTASFVFFGWITIGNTNVYGTIQDSRWKKEVALLNETAVKPLRTLPQPIYIPWQYQSDESYISTFVLETVGLLWTGHIVMTIDTFIGSVILHMSSQFAILQEAFITVYGRALSQLVSDMPLDIDMHDNLIMPTNDQTDILKNPLDKVEVKVKSFYTDKQIESAIEKSVLNCLRQHQLLISCVEKFRVTYSYGFMTQLLSSMAAICVVMVQVSQDASSFKSIRLVTSLAFFMAMIIQLAIQCFTANELTLQAERVSDAVMQSKWERMTPRVRRYLLMAMMRAQRPLRLTAAGFAYMDNGCFLAIMKAAYSYYAVLSQREV*

>HnubOR49

MLGYILNKLENPKRPLLGPNVKALEFWGLLLPKNTVMKYIYVFLHISIIFFTATEYIDIWFIKSDINLLLENLKITMLASVSVIKVSTFLIWQKKWRMIVEYVTESDISQRKTDDKINKSIIKKMTTYSRKITYLYWFLMYTTVAVVMVQPIIKYVFSQSYRDNVKSGEESYIQVVSSWVPFDKNTITGYLAACAFQSYAAIYGGGWITSFDTNAIVTMVFFKGELELLRRDSAAIFGTEKNPVSYDEARKRFKDCHRRHVNVVKYSSLFDSCLSPIMLFYMFVCSVMLCVTAYQITSGTSVMQKVLQVEYLMFGVSQLFMYCWHSNDVMHTSRDLIYGPYESTWWSNTVLRKDLYLLMAQYRKEVVFSAGPFTKLTLPTFISILKGAYSYYTLLTQSQM*

>HnubOR53

MQLSHCFNLSLFVLIVAGVWYPRSLDNTRILHVVNIYRVLAITFVLFGMLSIQFVYFFTVFGEDLDKKVDATTLFTFVGYMYKAIVVIINRQRINKLLDIIDTETDKDDLLKSMAANINLLSKFYNGGACLTAVMWNMIPFTKTTLTLPFYYPDLAPTSPWFVRWYVYQAVILIINGVAQTCADHLFGGLMAFAATQLKLLQHKLETVGTRTKSTLEIDAVEREQEDYYEAISCVEYHLKIIRFVDELTDIFGGAAFGQFLLAAPLLCLSMFIIMTSGDATEIVTRLLYFGCLSGQLFIYCFCGNMIKTQSELVATAAYNSCWAATSVRTQKALQLLIIRGQKTLSVVAGNLFELSLVTFGALLKSSYSFFAVLNKQRDDYN-IKRYQIGA-MYLNVCKDISHCKY*

>HnubOR54

MKNSDCLASAIAIMKYTGVWLPDNLQRRQKIAYIACGVVTQIFIFYFMIFAEIAYVYKHRHDVERMVDAAVLLLSHLVQAVKVMTVILRQESIKRLIDLGDGPDFTHADPKLRAIVDRAVKLTGLVGSLIICSAFVTSVFWCVVPALQDQLTLPLNTAYPFDTSGPYIFTAIYAYLSVSVTTVGMADAAENFLVSGLLMLAATQVDLLNQLLLDINAEGATVSYKKTVRCIMFHQRIIDYVKEIGRIFDVPIFCQCIVSSVILCMTVFKITVTREPIELVTMIFYLVCIFMELLMYCYPADVLLNKSLRVADAAFPGWSGDVNTARALVLIALRSQKPLTIDAGGMFRISLPTAALVVKTSYTYYALLQQTLSK*

>HnubOR55

MPSSLIALSNFISYAHLIMDSLTYHTSAAINHFKKITKIIYILTATNFWYEDIELPSVFVKINNISKLLEILIITLVVTGIGARYTQDGNLTEKQNTDVLMKSLSSFFVYAVYGCTVYHEEGIKELLLTLTVTLKGMFNDEKVERMMIKKTYQYLMGLIFVGCCPMVAYGVEGAVNALTSNATFTTIIPIWPDLEDRSHFAGFARILIYIVWLLLVAHVVCIYSVIISVSICLGHQYANLCEYFLSLHDIFDGEGRIEEMETRYENAVRDGIKMHCITLRCVNQLQRTCSVAYSGQVIINVCVMLLLMIQMMQTERSLVQLAPIVFMGTGVLVSSGLIIWSAGDITFEAQRLPTAMFHSGWYNCCGNSVRVRKMITIAMVQAQQPVVIKGLGIIELSYNSYVAIVKSSYSVFSVIY*

>HnubOR56

MWEKLRRFRPKHCDLPTTMDNLSVLLRVLALNPDGRDEGIPIAATVLTVLGIGVYVYVYVFSMVWSVFLRENEDLAGNMIMFSVGMATGMSMVKLFCVHIKSALVTKTVAAYLSCDKRVSRESRMYSNRLRYMRTIKRRATFVWLMTVTNGIAYCIRPFFQRGKHLILDAQTLYGLDPNFESPNFELTFVLVVMAVVVVVYSCATITSLLIIFVGYVEAQMLALGDEMLHLWDDAQALTTEETDPATQKKLQNQHIEHQLKSIVQTHAASLNLLHLTESVFSTSIAIEFCLLALDIISAMLGDLESTYLEIPYALNQVIIDCLIGQRLVEASLRFRETVYACKWEEFDASNMKTVLMILKNTKTMVLSAGGIAELQLTTLMYVLKSIYTTYAALRTTMNKNT*

>HnubOR57

MYSTFQAFRPHFVALSYVAYFKIVPRPSSELKLALHNAYRAVVWVLVITYNLQHVIRVIQARHSTDQMVNTLFVLLTTLNTLGKQVAFNARVGRMDRIIATIQGPLFAARNAFDEEVMKSNALEMSRLLQLYHAAIYACGLMFTVFPLVNKWLGEDEQLTGYFPFDTTGWLGFGIALTFNSILITFQAYGNVTLDCTIVAFFAQAKIQLQILRNSLEHLVDPVGAEGGRMCVQSKGYRDIEDPAFGLLLKKRLTRCVEHYKLIVWFTKEVEAVFGEAMVVQFFVMAWVICMTVYKIAGLSLLSAEFVSMLMYLGCMLAQLFIYCFYGTQVQYESEYINISVYRSDWLSLSPRFRALLLVQM

>HnubOR58

MTAKTYTYKTNDTVLLFHKICNLVYLGCGTNFMFHKLKLPTKFIKTFNHVSKIFEMLSVVLICSEWGAFVTQKNLTLVQKTDLFLFGPTSITLYVMYWNGVYRKGQIKDLSFTLAVTLKEMFCDSEIEKKMIKKTWRFVVAMVCIVFGFLLATGINNGYRALTTNATFTTMLPVWPSLEDHSKAAGICRVVHYIVWWIFVTRVTCMYFIILAIAICLQHQFRILHEYFLSLPGIFDGEGSQEEKERKFEDRLKHGIKMHSLTLWCTDQTQLTCGVAFSTQVITNVLSLVLLMVQWMNMERTFGNAVQVIMFANCMLIGTGIFMWNAGDITFEAAKLPTAMFHSGWHNCRGQSAARVRKLLTIAMRQAQDRVVIKGLGIIELSYESYISIVKSSYSVFSVMY*

>HnubOR59

MMETKLQAQKEIRATLSLCIFCMRCIGLSFERPANSVRFLCQKLMFVVSVCTIVYHVFSEIVYIGLTLSNSPRVEDVVPLFHTFGYGALSIAKVFALWSKKEIFAEHLGELSGIWPMEPLDEDARIIKQKSVSALRLVHQWYFSMNVGGVLFYNVTPICVYLYQLWQGLDAQVGFVWMSWYPFDKHQPLNHVFVYLFEVFAGQTCVWIMIGTDLLFSGLASHIALLLRLLHKRLESLADTDKSQEENYQEIVENIKLHQRLIRYCNDLEEAFTIVNLINVVFSSLNICCVVFVIVLLEPLMAVSNKLFLGSALIQIGMLCWYADDIFHSNADVALAVYNSGWYRTDPRCRRALVFLIRRAQKPVAFTAMKFTNLSLVTYSSILTRSYSYFALLYTMYNDS*

>HnubOR61

MSLTTKEEFICGMDYLSVISSRIFLYPFYGRSKAKLFYFHLICFLITFASIQQFISLCIVKLDSFLDIVSIAPNIGVCVMCVTKYVKINIHKKLYSSIFVHFRTHLWDVFSEESEENVRILKRYQRIIHFITLWFVYYVVPLILVVTSFPILIMYYDNKVLGKELEHRYPFEAWYPFDKVKWYYAAYAWESFITALVVCIYTFSDLINVSYVAYICLELKFLGTHLKELIEDEDISQLTKSHNVAAVHNKIRQKLRRIIIRYNFLADISSQLDGVFGDIMLVNYTFGSVFICLTGFTFTVVGELYSTLRCFFFLISLIISMLNQCIMGQCISDHSGQLVQALYDSKWTYGDRPTRQLILMQIMRMQKPFQLTAKGYIEMNLDTFTAICSTSYQFFNLLRTMYAPKEN*

>HnubOR62

MDLLTSLWRAITHTKALEESSGQMETAFFESVYRVTYIAGLSNTDHGFFYKLYSYTVKLMITLFMMGEIWYMISFLSTLDIFIEQMNVIVIQSMAVFRYKYMRMNEHVYKKLAASMQMSNIDTSTPARKALVESWIQRNERYLKILLGLGSLTLAAWYVYPLVDDLEYNLTVELRLPVDYRTPARYPIAYLITLIAFHYTAFFIMVNDVIMQAHLIHLICQYTVLADCFENILKDCEKEFKGLDRNELIRNSRFREAYVIRLGFLVDQHQHILEHTLELRKTISPPMLGQVMASGLQICFAGYQVALTLTVSFTKFFMSLLFLGYNLFELFVVCRWCDEIKIQSENIRHALYCSGWECGVGNMAGVRARFLLILARASKPVVLTAGGISDLSLNSYADLVKTSYSALTVLLRLRHD*

>HnubOR63

TMFEKLKRIYNKDDFDYSSGQVDPYKFHPTFYFIAKAFQVLDNEPIPIWSTVSFVAMVIDAIIGVVFAGISTFHGMQIFDIPTTTEAGIYTIVLVYKLLILSCTQLDKAQYHCFLRIMREDFRYVCAEGAKYRERFFENQLQTWKVSLASVIFTSGIAIGMSGFALMSLIFYLITRTPGDGSKRPLLVPFWLGDVDFGATPTYEIAFNFSNYCFLAYAYNYIFMIQTQVVWVRQIATKADLVIWAIQDLLQDIHPATCKGEKAHFAELIKYRMREIVSQHQSMYTLMEAYAGVYKKLLMFEQKLCGPVVCLTAYCTAEKLDEGEFNAILLLLCIATVTLVFIPCYLCTFLGIKVKSISDACWSLSFWNAGREIRPYLVLIMQRSLRPLPLQAPGFEEISIQTFSAKMTSAYSLFNMLRQTKL*

>HnubOR64

MPVLQTAKAFLYKTDFEWHEEITLQNFHPQLQIFLAINGVFFNNRESKIRFIWPLLSTLITMVAVGFEMIFIWHGVSTGDYSFATECFCYFFILGSVGIVYFSVLLNRKKVFELLYNMNNDFLLICNLKPEYRDSFLTGQLLIWRLCWSWIVFISFVSMLYISNTLVYLLYQCTLATPDEHMIRPLIFPMWLPEDDPYRTPNYEIFLVLEIILIFVVLLTFGLYVYILFHLLLHYYNLMDIIVLALDELFVGLDQSVVTLPREDPRREAVQAELSGRMGQIVRWHLSIFDSVDNISSVYGPTLVYQVMFSSVVICLVAYQVA

>HnubOR66

MPIKIASKPAVGPAAYRHNDTLGLLCLVCKVIYLSCATNFWFEDLNYQKIFMKIYDTVSKILEVIVVLFVISEWGPFYTQTHLNEKQNNDMYLFAFSHVVIYLVYASALYYKQELRKLVFTLGVRLKEVCNDAVVERMMVKTAGRYLTGLVFVCSSTLISYGFDSGVQSLTTNTTFTTVIPIWPDVEDRRFIAGVARLLYYIIWWIFIVRVISTYLIILTITICLAHQFANLCKYFKSLNSIFEGTGSQADKEKRYEDAFKVGIKMHSITLWCARQTQTTGSVAFSAQVIINVSVLVLLMMQMMVGIGNCRSSTTMYTERTLIALMPIFFMAASVLVGSGVFMWNAGDVTIEASKLPSAMFYSGWHNCQRESSVRVRKMVTIAIMQAQKRVLIKGLGFMELSYESYVKVI*

>HnubOR67

MYPFQLYYFLKIDTLWKYLMLYMTQAVVGTIIHTCMYSSCDQLLVTLTVDVCILLRMLQYDLENIKVEPGQEESLAKLKDIVKTHQKLLRLAENMNEIFGIIMFVVVLCSSVIICLFIFLTIVATAPLQLFKNFLAVTQVLSIVFYIMLPGQILSDTSSGVADAAYQSLWYDSDPKFRKIICIIIARSQEPCKLRALSYTDMNFNAFCKICSTAWSYVSLVNQMYQKD

>HnubOR68

MEHLEVFASEFFKPFAICFDFLAKSNISIHNKTKSAQGKLRSLILVILYVTFYFSLVLSLKKVFSGVLGFYELANLLPIFIVATQGAMKGAVIIANLSKARRVIDDLGSIWRSTGLTKKQQAKKGVMLKRLNLCNAVFYWMNIVGTWQYLLVPLFETIFRNFVLGEDTLLFPFICTLPFDATQNWLVYLVTYFYESYSMLHLIYMYLGVEFLMITLCSHLATEFELLREEMLLAKPILRGAREINNININREQNINTSNCADNDINIDGSDNDEVTEEEPWIQEVIRRHQKLIKLSQLLDDIFNRMIFFNLLFATITICFFGFVAKSAGVADSAYHNLWYQGDVRYQKIIIFIILRSQQPCSLTSMRYSPVTLNTFTTVLSTTWSYFSLAISVYETEK*

>HnubOR71

MVMGDLLFCIFLSHISMQFDLLSVRIRKLVKKTDPKHGFAAYSIQQMRNENYDGPVWEKGHLQELAAIVERHRALIRLSGDVEEMFSAALLLNFLNSSMIFCFCGFCSVIVEKWNEFSYKSFLFTALAQTYLLCSHGQRLVDSSKGISEALYNCYWYNSSKRVKSSVLIMMHRSQKEVHVTTYGFSVINMASYATILKTAWSYLSLLLNVYK

>HnubOR72

MSEFSNFEPISRDTYTLILDCIAKNQIYIWDENALLGRMCWLKLIVNILAVVSHVAGVIEKMSQGADLVQLSNELSATLILCQASLLHIQFCVNKKLIKNLIINLDSKWRTDDQLRPEMIAIKHESVQSFYKWVSLFNKCLFCFSHAYLLSRLVYVAICHLILRRDVVFIMTFHIKMPFQYDNDFILYCLVYLADSFIFLNVAYLVTSDLLLMNAAMRDLRLLFVILQHDLKNIAQPGEDIDHGTAERRLKAIIPQHQDLLQLMIQLSEAFGAVFFIHLAFFSGTMCFFGFAARINCNAESIQNLPAVAIILISIYTCCTSGQHLTDASMDIAEAAYDSQWQLMSHEYKKYILFIILRSQTAQYIKSTSFTDVSLTTFTKILNVTWSFLSLITKVYEE*

>HnubORco

MMNKVKSQGLVSDLMPNIKLMQMAGHFLFNYTDETGGMSMLMRKAYSCMHAFLIVIHFVCMGINMAQYSEEVNELTANTITVLFFVHTIIKLVFFALNSKSFYRTLAVWNQSNSHPLFTESDARYHQISLNKMRRLLYFICGTTCLSVVCWVTLTFFGESVRLIADKESNETLTEPAPRLPLKAWYPFDAMSGTMYIVAFVYQIYWLLFSMAIANLMDVMFCSWLIFACEQLQHLKAIMKPLMELSASLDTYRPNTAELFRASSTEKSEKVPDPVDMDIRGIYSTQQDFGMMLRGAGGRLQNFNTPNPNNPNGLTQKQEMLARSAIKYWVERHKHVVRLVASIGDTYGTALLFHMLVSTITLTLLAYQATKIDGLNVYAFSTIGYLSYTLGQVFHFCIFGNRLIEESSSVMEAAYSCQWYDGSEEAKTFVQIVCQQCQKAMSISGAKFFTVSLDLFASVLGAVVTYFMVLVQLK*

>HnubGR2

MPITRSRPGTITFSWKSRATMYAISFYIVATVVVLAVGYERIMILRSIKKFDDYIYAILFVVFLVPHFWIPFVGWGVAHQVAIYKTSWGKFQVRYYRVTGENLQFPNLKTQIVIISVGCLLLAVCFLLSLCALMDGFLLRHTTAYYHIITMINMNCALWYINCKGIKIASQSLSECFRRDVNVECSAKLISRYRFLWLNLSELLQSLGNAYARTYSTYCLFMFFNITIAVYGALSEIVDHGFGFSFKEMGLFVDTLYCSTLLFIFADCSHKSTLKVAAGVQDTLLGIDVLSVDRPTQKEIDHFIQAIEMNPAVVSLKGYAHVNRELLTSAISMIAIYLIVLLQFKISLPKTEADS

>HnubGR4

MAKIFGYARWFGVAGSGNVSWKAFGFFVLLLLSVIEGVAIWRVIKALAGWALDTVGHRSVTARLAGATFYASSISTLILSWNLSSSWGKIASYWTMVDRSMAINVPPDKSLKNRMITVTAVMVTCVIVEHAMSMMSQVGFDCPPSLLLKRYTLMSHGFLLLRTDYSIWFAIPILIMSKIATVLWNYQDIQIVLISMGLASRYYRLNQYVAKVSALSKDIPWNTQAECSKENTWRRIREAYVKQAQLVRRLDQALGGLILLSNLVNFYFICLQLFL

>HnubGR5

MLKTKPISRMYYVSEAKLINSKTDLLKHYDSKSQPDIFVTSLLTIDHKNYPATFQEAMKLTITLGQVFGLNPVCGIFEVDTIKLRFQVQSWRFAYSFLSIILQSVFVCFSSLKLFSDTNPNLSANASLVFYTTNCITTMMFLRVAMKWPGLCQHISKTEASDPSVDRALIKKCNVSCVLVLSLAALEHLLSDLSSLAGAIDCQKGKNLFEAYSIASFPWIFQYTGYSPYMGILAQFINLQFTFNWNFSDVFVICISFYLASRLEQVNRRIVAVYGKHAPSSFWRSVREDYSRVTGLVRRVDDVIGGVIFISFANNLFFICLQLLHTFA

>HnubGR6

MECYPHTNIYEGFVAVFYPWVYKYISYTTMLGITTQVLHFQATFIWNFSDLFVICISYYLTSRLDNVNRKLLMAQGKYMPASFWRTMREDYGRVTQLVRKIDEVINGIVFISFANNLYFVCLQLFNTLEDGIKGNGECRANGHRGRSVAVSLIASQVNTASSVPAPVLYDVPSPVYCIEVQRFIDQVNGDDVALSGMQFFTVTRGLLLTVAGTIVTYELVMLQFTPTPQTAGSIPPNITVT

>HnubGR55

MLLPKKGNFTKALIDYFHKIYLHVGPRTLHSLINQKYWIISARSVIRSRFSKCLQCFKATPTSLQPIMGNLPKFRLQDIHTFHTVGVDAGGPFYTKESNRRNAKISKSYLLLFVCFSTRAVHAEVLSALTTECFFAAFDRFTARRGLCHTVVSDNGKNFVSAGKQLAEINQFYKQNTDEISTCFNNRRVNWKYNPPTGSHFGGMYESGIKSAKQMLKRVIGTRSLSFEELSTLFARVEAVLNSRPLSSISSDPSEYEALTPGHFLVGRELLAVPEYDLSDEPIHGLTRWQLVQQASQRFWKLWKNDYLHTLQQRQKWLSKCRNLNVGELVLIHSDAPPLQWSLGRVTELHPGPDGVVRVVKVRTQNGEYTRPVVKLSPLPMELDSD

>HnubGR58

MESDKVVNRKMKIKRIVPSECLIKNIIDKDFQSMIFPLNIFQSLFLISKFSITNNFITPKSTIWHSIQSAIGTSVLVLSHFLRFFYYFNTLDGLRTTVVTFLLYFDLVFYCIISIIIYIISASQSGNTVELIIRLQKVLNSIEIDKKPFYSTIKIENFISVVSILIVHICFWLSLININLGFISAFISFLSSAPTMIFDLHIVTATRTVVLIKKELAAWNRILKGCKTISIKTEEEIRNNEISGKVMFDAYIDILRAFRLSQKVYQFAVTCFGFQTFIHILVNLQVVLEFGKSTTGSEFFQSLQIRLTTGAWNLKNFILLTTLSIACENFYGTVKVADSICAQILNSVQTTDMHRACKNVLRVNRASFQKLSAYGLFEVEGVYPVRMLSGLVAYTVVLLQFAFL*

>HnubGR60

MLLDNAIDKDFQMMLLPLNILEFVYCQPKYQITGAFVTPNGLRGKIHCIIGVLIVIFTNIINTINTGFVHGNAISETIFKLSLADAVFYSVSGIFVCVTILVQDNRNVQLIIKMQNACRILHRGGGLKKMQRRNWIFVTTVLMAYFLYNLTYTLCNIYKITHLVFNMILFYFDVNIIIAARIVKFLEYEIILWRKELQRFQRTCSQTNHKQLVQYLVNVDLEEKKLFEAYSDIIGAFKLCSSIFGTS

>HnubGR63

MFGCSLYFIDLYFIYKYSGDSFIAYHKTYSDIDKHFEMKYHRVIRSKVNHSIVMFLFPWFVSSVTDFAVWCLFFDWQSTAVYSIVYFAFLMRILTVLELNSHVMLIGYRLKAIADVLNDHVSLADDTPCRIKIVKKSPQIHMMYKTKIVPIKTRATSEIVTLSSYYLLLIQQANFLNNFFGIRILLYCLYSLSEIISLVNLMGRIVVGSVVTVHLINSTSLIKCSKPDERRRNYFSAFRSEEITLF

>HnubGR68.1

MYSYLKTLISPSDELNVFSAFKPMYITRSVFGLMPYSLKFPKSQEGAIIIHKSIYFNSLCSVSIILLLYSCCFLHVQHVITATETNTFTEVLMTKANYLIELFGFLVFSTVTYFCAFKNRFTNVKILNAIVSSSNKLFYHNQDSLREIHVQVKIVIGCLCLLLMAQLTVNFTRHETMWSITLVTLTFILPQMIQFATISFFYVLILMVVGILKNINGQLINIVKTRKIYVSRVKKVEIKTVDFRLIETVFDKVLEIKREINKAFQASILATAIQCFHSLISETYIIYHGVSIDSSFTVHDFFNCFTWIGYQLLKIYTISRTGILLKAQVNIHFHESTVVR

>HnubGR68.2

MFATLRKYFSPFVNHDEELSFLQIFKPLYGVLSIIGLFPQAIEFPDGKQCNKVVIKSTRINLACTLLIVLVIHGFLVLHLLELSVDSKNSSMTEDGMTVVNYTVGLIASVLFCTVSYFSVIRDRSLYITILNAMDDCWDRLAKEDRKVLLGRLRVQVNCVVLGSVLVTTILLNIATYTGDYNIWKMILVGLTFVLPEMIQFVVIAFYFVMALMLVALLKNIEEQFKTILRARNNVQKYCDKADVGLDIAMAEVRDVYVKTMGIKRQINRAFQAPIMVALLVSFHELISMPHMIYHGLAFQSNFSSHDIFECSCWLFNQLVKMYALAHSGSFLRSQVNRIGRTIHKIPTSADQDMKIFLEVQHFSSLMAFQETQITVYGYFSLDATLMFNIVVSATMYLVILVQLDKHD*

>HnubGR68.4

MFPVLRQYFSPFAKRSEELSLLQIFKPLYIVLSVLGLIPCSIEFPKGKLDCIILHRSAFNHSLCALFTLLTVYIFFGLHVHQVLTSHEENAFADDKMAKTNYIIELVTQCLFCTSAYFCAFQRKNLYVSMFREVTRSWDDLPYVNRGIILGRLRVKVNCVVFGSLLLILITLTAVTYAGSTNLWKRILITLSFNVPEMIQFTVVAFYFVFVLMVVALLKNVEDHCKMFTKARRSIKNNSYKVESGRIPVTLSHMQSVYVKALRIKRQINVAFQAPILFSLLQCFHTMVSESYDICQGLLYQDDFSSHNLIECSYWVILQLLKIYALAHSGTLLKKEALRIGRTIHNIPSIDDDEIKLFLEIQHFSTLMTFQDTEITIFGYFPLEAPLMFNMIAAAAMYLIILVQFAKAK*

>HnubIR1

MQMWRFIFLVAGAAGLPWESNMAADYFLHKAVKYVTYLSCKDSADIKALSRLSMKEGIRTAVGVIDQDPINLERLLYQWDAAVGVLLDADCRKTQDVLNDASDSTLFVDTRSWLVVSETCNDTALIRELFQDLKLSVDADVVVASHCGETYQLTDVFNFGRIQGNPLETRELGTWSPERGLEISPQGFKYYNRWDFHNLTLRAVSVIRSSSKDFDESMLFEPGYTVGVAAMTKICSQLLNVLKEMHNFRFNYTIVGRWIGTPERNSTKAMSNTLFWREQDISSTSARLFSAWLDWVDPFFPSVTELETKFYYTISDKGVGDYENRFLTPMSPGVWWCSAAA

>HnubIR3

MFRRSLLLFLVSSVAGLEDNVIDFSLEFFKTRNVYSVCLLTCGDKAWTRRFTRNASNVSITVAHVSIHETLTEDLVSVDSCLNSVVTSTGVLVDTKCPQFEDVILYASENLFFDANHKWLIIDVDTRLSNMTTYPKNVEMNENLSWLVETLEQVNLSVDADVTISLQKGSENNIYEIYNFGKIRGGNIVVKKLGSWRNRADWIYQLNVYKYYKRWDLENSPLNVVAVMSTPPKIFDVNMIIGNNPYPGVAIITTTASRILEEISKLHNIRYNYTIMDRWIGDFKRNSTRVAANSVYFREQDITPVLRMPPEIFQKLDMLLPPLTAIETRYYYRIPTTGPGKFENQFLTPLTDQVWACVTAVISLCALVLFLTAKAERRPAAAQYAVFSVAATFCQQFFDDNDNSGIDDPRRKSSARQLTVLVTGVSCVLIYNYYTSSVVSWLLSGPPPSINSLQELLESPLSLIFQDIGYTRSWLQNPTYYYNKKNAEVEDKLRKYKVFRKKKGEPLLVPLEEGIEMVKAGGYAYHTEVYNANTLISRKFTQEELCELGSLQSMEKTLLYASIPKKSPYKEFFNWK

>HnubIR4

MRIATPMGHYDESYSGTFADYVHDNTRPERDSAIRCGYGASSLVLEWLKAKEVLTQVEMWGTEVGNRSMFTRLADGTSELSGGILRVQKKRLSKLDYIVPLWPFKVGFTYVSERESSSNMFVMPFTGATWIACAVITVILALAQRATAMAPSEKEGAFVAVLATWLQQDASAVPEGASGRLTFMVLSVCSMLVYAYYTSAIVSALMSAGGGGPTSLRALADSRYSLASEDYDWIRYHMFDVFKPDWPEMEYLKRKKLQSMTNFYMGTEAGMQLVKEGTIAYHAEYNHVYPLMKILSDDQVCKLQYVHTVPPVMTWLITTRRGQWTDVLRTGGGWLLETGLGKRMVSRWQVKPPPCRAALLAERVSYGDVAPLIILTAVGFIAAIALLLLERSVFKWRMKVKPIGSDDAQEEDMID

>HnubIR7d

MVLHNSTIILGLSAFMQIYQKSVIIGKGTFLHGAESSGDRIRQFVVFGSDLTNIKYMLDWMRKRQFDNTGKFIFICKNCDEREAMNIFWDHKIINLVFIKDCDSTSKTVGFTYSIYDNENCVISPPVPLQESCIRNSSLEMFPMKLKNFHSCQVIVSTFIQVPFMSLNTGIPKGVDGDLLVLIGEALNATLKVMTPHQGDGWGMPDSNGNWMGSFGDIYNDLANLSMTSAAITITRYKEFHMSTEYNSINMVWVTHPAKQLPAWQKLLRPFRMKVRISLAVTYLLVVLVAVLIRSNLWMKLTRKITSARPRTSVIFYSWTICMGMPTTSMPSKPAFVTIFLLWVFYCFMIRTFYQAALIHAMKDDSNYPEFENLDDILNSGYPFGGVPSLKDYF

>HnubIR8a

MRPEPSFYAVVGGAAFVADTYKRAVKEKLVRRDYRWNLVLTDYSEGKELQPTLPTMVLHVDSAECCKIMGQKDGCTCNQDFERKQPILSALLQLLAETYSKLEDESLTTRVDCDNVVELNGTRARVYRQLAEDSGASNESLFYWDAERSGIFLRSRFILSTLTPDTGLQNVATWSADEEYKLLPGMTLEPLRQFFRIGTAPAVPWTMPKLDPNTGEPMYNEDGQPLYEGYCVDLIQKLSESMDFDYEIITPKVGSFGRRLHNGSWDGVVGDLMRAETDMAVSALTMTAEREEVIDFVAPYFEQSGILIVIRKPTRKTSLFKFMTVLRTEVWLSIVAALVLTGFMIWLLDKYSPYSARNNPQAYPYPCREFTLKESFWFALTSFTPQGGGEAPKALSGRTLVAAYWLFVVLMLATFTANLAAFLTVERMQTPVSSLEQLARQSRINYTVVEGSTIHQYFINMKFAEDTLYRVWKEITLNATSDQSQYRVWDYPIREQYGHILLAINASMPVPDAKTGFRQVDEHTDADFAFIHDSAEIKYEVTLNCNLTEVGEVFAEQPYAIAVQQGSRLQEELSRALLDLQKERLLEQLAAKYWNETARQQCPDADESEGITLESLGGVFIATLFGLGLAMITLAWEVFYYKRKEKNKVQGLEPEQKPKKAFEKDLEKKIVQSSAGSAEGAADGVAKLRKRDKKGRVTIGDTFKPVTDKEGVSYISVYPKSEYRP

>HnubIR21a

MTYFKIIILNVLFIHNAISQEIEYYPSQASIFQRKLVREMKPEFYLYKHVVYKREAKLRIFNEDNKFDATKNNTQKRAVDPVFHGHPKTREELWNEKFINETLAFDQTPSLISLIHNITLTYLNDCIPIILYDSQVKSKESYLFQKLLKDFPIGYTHGYINDNDELAEPKLLRTNKECINFIVFLADVTRSAKVLGKQAESKVVIVARSSQWAVQEFLAGPESRMFVNLIVIGQSFKDGDDDTLEAPYILYTHKLYTDGLGASQPVVLNSWSHGKFSRPINLFPHKMTEGYAGHRFVVAAANQPPFIFRTIKTDLDGGNPRVVWEGIEMRLLALLAERNNFSIEVKEPLEPDLGPGDAVLKEVTGGRADVGVAGLYITSDRTRDTDMSFSHSSDCAVFVTRMSTALPRYRAILGPFHWTVWLALTLTYLFGIFPLAFSDKHTLRHLLHNSGEIENMFWYVFGTFTNCFTFVGKNSWSKTTKITTRLLIGWYWLFTIIITSCYTGSIIAFVTLPVFPETIDSIQQLLDGFYRIGTLDRGGWEKWFLNSSDPKTKKLLKNLQLVQDVPSGIRNTTKAFFLLPFAFLGSKAELEYIIQSNFTQTRKNRKAQLHISNECFVPFGVSLTFPNNSIYTAKLSGDIARMLQSGLIYKIEDEVRWEMQRSATGKRLSVAGSGSLKLGAVAEKGLTLADTQGMFLLLAAGFILAATALVSEWMGGCSRKCRRPKKEDAPISANSREHLIPSPKSDIDSEIKIISDSAESRFFLDPRPSSEESRDSLEGTIINVTKENIIIHDNFSVDGWDSRRSSSVDIDKEVQEIFKKDENRRRTLSGVNQLTESQRQATASKGAFGDHLSDH

>HnubIR25a

MSALTILLLFLFVPVSFSQTTQNINVLLINEENNALAEKSFEVAKEYVRRNPTLGLAVDPVIV VGNRTDAKAFLENVCRKYNDMLSAKKTPHVVLDFTMTGVGSETIKSFTAALGLPTISGSFGQAGDLRQWRNLNANQTKFLLQVMPPADILPESIRAIVTKQDITNAAIIFDEYFVMDHKYKSLLQNIPTRHVITPVKSFNRDEIKTQLRSLRELDIVNFFVVGSLRTIKNVLDAADENQYFGRKTAWFALTLDKGDISCGCKDATIVYMKPTPDAKSRDRLGRIKTTYSMNGEPEITSAFYFDLSLRTFLTVKSLLDSGKWPNDMRYITCDDYDGKNTPNRSLDLKSAFQEIKETPTYAPFFIPEDDPMNGRSYMEFNTDLSAVTVKDGASIGSRNLGSWKAGLSNPLSLTDPQNMSDYSAQLVYRVVTIEQKPFIIRDDDAPKGFKGYCIDLIEEIRQIVKFDYEITLAPDGNFGTMDENGNWNGIIKELMEKRADIGLTSLSVMAERENVVDFTVPYYDLVGITILMKLPRTPTSLFKFLTVLEDDVWLSILAAYFFTSFLMWVFDKWSPYSYQNNREKYKDDEEKREFNLKECLWFCMTSLTPQGGGEAPKNLSGRLLAATWWLFGFIIIASYTANLAAFLTVSRLDTPIESLDDLSKQYKIQYAPLNGSAAMTYFERMAHIEVRFYEIWKEMSLNDSLSDVERAKLAVWDYPVSDKYSKMWQAMKEAGLPNSVEEAVQRVRDSESSSEGFAWLGDATDVRYYVLTSCDLQMVGDEFSRKPYAIAVQQGSPLKDQFNNAILQLLNKRKLEKLKENWWNNNPEAMKCEKQDDQSDGISIQNIGGVFIVIFMGIGLACVTLGVEYWWYKWRKRPVIGDVTQVEPSKTTRNNADQDTTKVGEGFTFRSRNLGLSNFRSKY

>HnubIR41a.1

MMLTPPSIFLPIEIVVNTLIYNYLQSSFCLTFVTETHLAVKLPSNLSSLRIQPNDSDLVNQILYASEKGCSDYIIQMYEPEDFMVAFEKVNHLGDIRRSDKKLIFLPMQDEIYNASVLTDMLSLKEASFVANILLVAPVVKTSDDCEFYDMITHKFVGHDEEIHKPLYLDRWDSCAGHFQKAMNLFPHDMSNLYGKTVKVTAFTYKPYVLLDLDPSLNPIGRDGIDIKAIDEFCRWVNCTVEFVRNDGYLWGEIYENSTGVGIVGDVVEDRADIGITALYSWYEEFRVMDFTAPIIRTAVTCIAPAPRILTSWDLPLLPFTWLMWVCLIFTFFYASFALFLAQRSTDKIFLSTFGMMMTQSRDDSSDTWRIRSITGWMLVTGLIIDNAYGGGLASSFTLPKYEPSIDSVQDLVDRKMEWGANSEAWTFSIILSEEVIIERDSSSIRASAIV

>HnubIR41a.2

MLIPPSIHLPIEILANTLIYSYLQSSFCLTFVTETDLAVNLPSNLSSMRIQPNDSDLVNQILYASEKGCSDYIIQMHEPEDFMVAFEKVNHLGDIRRSDKKLIFLPMQDEIYNASVLTDMLSLKQTSFVANILLVVPAVQSSDDCEFYDMITHKFVGHDEEIHKPLYLDRWDSCAGHFQKAMNLFPHDMSNLYGKTVKVTAFTYKPYVLLDLDPSLNPIGRDGIDIKAIDEFCRWVNCTVEFVRNDGYLWGEIYENSTGVGIVGDVVEDRADIGITALYSWYEEFRVMDFTAPIIRTAVTCIAPAPRILTSWDLPLLPFTWLMWVCLIFTFFYASFALFLAQRSTDKIFLSTFGMMMTQSRDDSSDTWRIRSITGWMLVTGLIIDNAYGGGLASSFTLPKYEPSIDSVQDLVDRKMEWGANSEAWTFSIILSEEVIIERDSSSIRASAIV

>HnubIR60a

MHSFKLVCKNIGIMLLIVIFLSLQVTAKINPNGPTVVADFANCVSNLVDANFVEPGLIYFVNSNDVSTPVAGIRNAILKSLHAKLQHSIKIGKPTKKDKGLCTDNENKVLEVSFGLYMDNFEATPLADYFIVIVEEFKDFTYIASRLTRSRSWNPQAVFILLYFGINNSDDQNIRHAEDMLFCLFKVNVVNAVVIIPKVNDVRKANIYSWRPYDPPKYCGYYNESIKNRLYVENICERGVVKYAKNIFQSKVPSDMKGCVFKVLALERQPFISRDPKGPNIERLLIDQVAKRYNISLRYEILNSFRGEKQFIGDWDGALRDLTAKKGHLLLGGIFPDDEVHQDFECSSSYLADSYTWVVPRALHQPIWLALFIIFQKTVWLSVIACFIFIALSWKVLAKLSRDPTHRANLDHYFINTWISNLGFCAFSRPITHSLRLFFVFLNIYCILLLTAYQTKLIDVLTNPFFEYQISTVKELVESGVKCGGSEELHDLFENSTDPFDIHLLEEWIDVVDIRDAMRDVAVHRNFSLLCSRLELAYVSAIVPELSDNFGSYKYYAFPSNVFSVSMEMVSLRGFPFMKGFSSTLTYFRQYGVNERVRTYFAGYVLRQRALLINELQSEFSERDALSLQTLQGGYLALLFGYVCGTLVLIVEIVVNTKFVKNIKLFKKSKKKKIRF

>HnubIR64a

MRFYVNPSMNLITYSTLLTIFSTAEIYLIKDVFKYKNLNVGTLFHCSNPENVINLQRFLNKIDLRVSSIRMGGNTSRFKQTSGIRVGIIIKTSCDNWTQVFELFNSNLFKKAQYSWLIFADDVSSTSEELLTYPIEVDSDVTIISQSEKFFNFYEVYNTGYYTNGRYHVEPVGHWYNKLWMKPHRRTDLSGVVLKSPVVVTHNIDHQTFEEYVSRSKPELDSLHKLKYFTLLKYLREMFNFSFVLQRTNSWGYLTNGSFDGMVGTLQRREADIGGTPVFIRPDRAKFIHYVTATWSSKPCFIFRHPKHPGGFLTIYTRPLSAEVWGCIVALLVLAGSLLCGLLKLGAVMLVGDEGDSSASLALLSVWSAVCQQGMTVNRRSTSVRLVLFSSFLFSLFVYQYYNALVVSTLLRVPPVTRSLNDLLHSKLKAGVEDVLYNKDYFRRTTDPIALELYARKIASPHPHYFPPEHGMALVKRGGFAFHADTAFSYPIIRRTFTEREICELQEVQLFPPQIMFAVTKKGSPYIKHLSYGIRKMAESGLMQRLKNSWDEPKPSCVRTPDSSIFSVTLREFCVPLMILCAGVVAAAGVLLAELIVFRAMQDPV

>HnubIR75p.1

MLFCNSICGIVASKRMSRFQFSIFCAKGDFIESVLSYCSRKTIIPVHKTARGRPTILTPRGVFHGILTDVRPHRELYRRRCDVMGAPIAMVNSIQSSNSSINHILNEDLLELEHDVMARNSWSYAKIGFLMLNATPVAKFSTRFGYLKNGQWTGTIRELIDYKGDIGTNIGVTSARLNAVTYIDVMDNSRPRFIFRQPALSLTSNIFSLPFSSGVWMATGITSFAAGLAFWLSIKLIGRTTDSDAAMGGDGAIDDEMMRDAFLVTMGAISQQGVEVHPRNVSARIILWVIFTTLMALYAAYSANIVVLLQAPSTSINSLEQLAKSKLTLAALDVDYNHFLFKGGMDPVRNEISKRVDPDKGPKAFYDITEGVEKIRKGMFALHGLVEPVYRQIEKTFLEPEKCDLVEIDYLGFSGMYSPASKKSPYVELLRVVYPRLREVGIKAAINNRFETSKPACKDSAAKFSSVGVTELWPVLIFMMYGVALSVGVACMELLVFHANRFISTRTRRRRLIEFNFFEPQQKES

>HnubIR75p.2

MKIVVLFAVFCLSLVQGLDNNDVNMIVSFATLDERATSVLAPFVCWSVYELSTVAKSLRDAGIATAASLQPNRPEFHLQNLVILADLSCRGTDEFLIKASNEGFFKSPYRWLLLSRDPAELDILEQLLMLVDSDVVLAQKRSEDYEFIEVYKITENSELIYNTRAFWRSTNNKSKMNTTRNEETDTALVIEDKSTSIAIVDTEYGIIEDYRKSKVLSRRRMNLRKHILVMANVITDSNDTRKHMDDRLNLHQDSITKMSYMVVKICFEMMNSTEKLQFSNTWGYVDKNGSWNGIVEMIIKKEADLGTLTIFTQERMRIIDYIAMVGATGVRFVFREPPLAYVSNIFALPFTGAVWVAVLVCVLACALFLYITSKWEATMGMHPMQLDGSWADVLILIIGAVLQQGCTLEPRHAAGRVVTLLLFIALTILYAAYSANIVVLLRAPSSSVRSLQDVLNSPLKLGASDFSYNRYFFKKLNDPLRKEIYNKKIAPKGKKANFYSMKEGVEKIRKGLFAFHMELNPGYRLIQETYQEDEKCDLVEIDYINEINPWLPGQKRSPFKDLFKINFIKIRESGIQSCIHQRLHVGRPRCLGAVNTFSSVGIMDMYSAMLATLYGMLLSPAVLLMEIAYKRLMVIREKKRMRQDKY

>HnubIR75q.1

MNFFVTIWCVTFMGLPIASLISKENDIKVIVDVLQSFHKPSNVIANLCWKSSTIKYLVTTLAELENPKSVKVVDEVKDKDLVSGGKNMFLVDVACKGVGEFLDKAQSRKYFARPYRWFVMNRPNTKKKVPSELDEIDILPDSEVYVAQSKNSSYNINLIYKINRNCDWIIEEYASWSTGSGLNMSSRVKENVIAIRRRNFQREPVVTSMVVSDNNTIADLELMKYKEVDPVSKSGYNQMTPLYEFINATRRLILTNTWGYNVNGSWTGIMSHLSQGKADLPGSVMILTNERMQILDYLSFPTPSSIKFIFREPPLSYQNNLYLLPFKSSVWYCIGSFVVVLIIAMYVNAYWEARKTAADEKKQDGSMVLIPKVSDVTVFVMSAISQQGSTVELRGTLGRFVIFILFLVFVFLYTAYSASIVVLLQSNSNQIRTLADLLNSKLELGAEDTPYNRHFFMTARDPIRKAIYEKKIAPSGSKPKFFNLVDGILQLQKKPFAFNSNLGSAYKVIEKYFYEHEKCGLQEIAFIQGNYPWMACRRGSPYREIFKLGLLRNAEHGLNDRVNRIMFSKKPVCTVRGGSFVSVSLVDCYPILLLLLYGMILGVLLLLAEVLHHRRCQLFSR

>Hnub75q.2

MKLLYLIVFLFNSCYAENESKPAMVADVINAMQRPAAVIAMLCWSPHLKLRLYTALEGENVTQIHMMQFLKMGTVPERHAQDQQIVFLADLDCPDIASYFQASNLKKHFRSPFRWILVENARNSALNYESYIPDAIADIDVLVDSEVILARDLGDGSYELHLIYKIGANTRWKKEFYGTWHESNRLQLQVTEGEISLRRLDLEGYEIVICYVLTDNDSVNHLFDNVNDHIDTITKASFPTTNHLLDFLNAGRKYVFANTWGYRVNGTWNGMTGYLVREEVEIGGSPMFFTTERISIVDYISSPTPTRSKFVFRQPKLSYENNLFLLSFKATVWYSSIAQLLLLMIVIFIVASWEWKKTHRHEDKQKEADPGILRASLADVSLLIFGATCQQGSTVELKGSLGRIVMLILFLALMFLYTSYSANIVALLQSSSSQIKTLEDLLHSRIKFGVHDTVFNKYYFSTATEPVRKAIYETKVAPRGSQPGFMTMEEGVKKMQKGLFAFHMETGVGYKFVGKYFQESEKCGLKEIQYLQVIDPWLAVRKNTPFKEMFKIGTKRIQEHGLQTRENHLLYEKRPRCSGREGSFVSVSMVDCYPALLVLFYGTLCAISLLFIEIIVARRNEILRKMNRMNTLT

>HnubIR76b

MAGMELIISSICNATFCEAVYDNNFTDTPLTKHQEELMGTKNELNGKHLKIGTYDNYPLSWVTTDNGTLTGNGVAFVVLDILRERFNFTFEVVTPAKNYEIGVRGDLEDSLIGLVNSSEVDMAAAFLPIVNKYQQYVDFSSILDEGTWMMMLKRPKESAAGSGLLAPFEIHVWYLILAAVLSYGPCITFLTYLRSKLVKDEEKHISLSPSFWFVYGAFIKQGTTLAPEANTTRILFTTWWLFIILLSAFYTANLTAFLTLSKFTLDVEYPEDLYKKNYRWVAPEGSTVQYVVNDPESILYYLSRMVTNGRAEFRSVNVDRQYLQFVSGGAVLVKEQTAIDHLMFEDYMRKSKEGVTEVKRCTYVVAPNPFMEKLRGFAYPKNSKLKKLFDSVLTYLLQSGIVKFLEHRDLPSTKICPLDLQSKDRKLRNGDLSMTYMIMIVGLAAAIAVFVGEMIIRHYVRIKIRSRGNRKIKTAKTSKRRSLKAHDETRPPPYESLFGQNSRYKMNEKLMKAKIINGREYWVVGTVSGDTRLIPVRTPSALLYQRDK

>HnubIR87a

MIPLSLLPDSSKMHSRLFLSYLCFIHFSAAKSENPLLMASGNSGQIEEIAECVLKLSAKYFVEKKALSGSIVIININSYASTTQRLLLRTIHSGIKYSIMVKDSFYKHANASHFPEKAKNYMLILEEKSELVRNILQLNKLPTWNPLAKAIILYQLLPGEDGETISKRFINELRDYKLLKSIIFIFSPDDAELISYTWSPYSDTNCGGECDSVYILDKCKKSIVRQKNPQKETFPLNMKKCPLVTYAIVSEPYVMPPVRKLTDTNYDDAYEFQKGGEINLVKLISEFTNMSLIVRISDVPENWGLIYSNGTATGAYAVLRNDSVDLVIGDIEVTRTIRKWFHPTVSYTQDEMTWCVPKSAQASTWNNLVIIFQWTTWVATFLSIVLMGLVFHYIYYVENNRMVTKLPTNSMLYTFSMLLGWGASFRPKTATFRVLIFAWLFFGMIMSISYESFLRTFLMHPRYERQISSETDLIESGIPLGGRAIYRSYFETNNASSFYLYRKYISTSFAEGIQRAALNRNFAVVASRRQAEYQDQKLGKGEQLVYCFKEGSNLYKYGVVLLARRWFPLLARFNNIIRSVSENGLIDKWNQELFIHKVGGVESTGNIAPLGIQHLLGAFMFIGIMYAVSVAVFLGEVLLSVLAKRRAYKVSVHTVKLI

>HnubIR93a

MRIWVVLVCFVSASAEDFPSLITANASVAVVLDRQYLGEQYQPILEDLKDYIKELARVELKHGGVVVHYYSWSTISLNKGFLAVFSIASCEDTWSLFSRTEEEELLLFALTEVDCPRLPPDAAITVTYTDAGQELPQLLLDLRTTRAFNWKSAVILHDDTLNRDMVSRVVQSLTSQIDDEDVPTISVTVFKMKHEINEYLRRKEMHRVLSKLPVKHIGENFIAIVTSDVMSTMAETARDLFMSHTQAQWLYVISDTDIHNSNISSLINALYEGENVAYIYNITDNHETCKNGIMCYSEEMMNAFISALDAAVQDEFDVAAQVSDEEWEAIRPSKIQRRDMVLKHMQQHIAANSKCGNCSTWQAMAADTWGSTYQNFVEANGAENETSGVIEKIELLQVGYWRPIDGMRFTDILFPHIAHGFRGKMLPIITYHNPPWTILKANESGSISSYSGLIFDIVNQLAKNKNFTLKLIFPGDLKDILSNKTASDDMYSQSAMLTMMAVAKKHAAFAAACFTVLSDKNPGINYTVPVSTQPYAFLIARPRELSRAMLFLLPFTTDTWLCLGFAVVLMGPTLYLVHRLSPYYEAMGVTRQGGLATIHNCLWYI YGALLQQGGMYLPRADSGRL

>Hnub-iGluR2

MRGAKAIYLILFFGHLSALPDTIRIGGLFHPEDDKQEVAFRYAVERVNADRAVLPRAKLLAQVETISPQDSFHASKRVCHLLRSGVAAIFGPQSAPAAAHVQSICDTMELPHLETRWDYRTRRESCLVNLYPHPAALSRAYVDLVRAWGWKSFTIVYENSDGLVRLQELLKAHGPSELPVAVRQLPDSHDYRPLLKQIKNSAESHIVLDCATDRIRDVLQQAQQIGMMSDYHSYLITSLDLHSVDLEEFKYGGTNITSLRLLDPERADVQRVVRDWVYDEARKGRKLQLGHTSAKENMTFIKTETALMYDAVHLFAKALHDLDTSQQIDVRPLSCEAEDTWPHGYSLINYMKIVEMKGLTGVIKFDHQGFRSDFTLDIIELTRDGLQKAGIWNSSEGVNYTRSYGENQKQIVEILQNKTLIVTTILSAPYCMRKEASEKLTGNAQFEGYAIDLIHEISKILGFNYTFKLAPDGRYGSFNRESKEWDGMIRELLEQRADVAIADLTITYDREQVVDFTMPFMNLGISVLYRKPIKQPPNLFSFLSPLSLDVWIYMATAYLGVSVLLFILARFTPYEWHQTRSPDGEKMENIFSLANCLWFAIGSLMQQSCDFLPKAVSTRMVAGMWWFFTLIMISSYTANLAAFLTVERMDSPIESAEDLAKQTKIKYGALKGGSTAAFFRDSNFSTYQRMWSFMESARPSVFATSNKEGEERVMRGKGAYAYLMESTTIEYVVERNCDLTQVGGMLDSKGYGIAMPPNSPYRTAISGAVLKLQEEGKLHILKTKWWKEKRGGGSCRDETSKSSSTANELGLANVGGVFVVLMGGMGVACVIAVCEFVWKSRKVAVDERVYF

>Hnub-iGluR4

MQGLTPSPMSILDSLCKEFLAVNVSAILYLMNHEQYGRSTASAQYFLQLAGYLGIPVIAWNADNSGLEKRASHAALRLQLAPSIEHQTAAMLSILERYKWHQFSVVTSAIAGHDDFIQAVRERVTALQDRFKFTILNAVVVKKPADLNELVTSEARVMLLYATREEAADILSTAGDLHLTSENFVWIVTQSVLGSMQQPNKFPVGMLGIHFDTSSASLIAEIATAVKVFAYGVESYIQAPENAHHPLGTRLSCSGTAVGEARWPTGERFYQHLRNVSVESEAGRPSIEFTPDGELRAAELKIMNLRPAIGEQLVWEEIGTWNSYPKERLDIKDIVWPGGLHTPPQGVPEKFHMRITFLEEPPYINLAPPDPISGRCILDRGVICRVAPESEVAGLEAGTAHRNSTLYQCCSGFCIDLLQQLAEQLGFTYELSRVEDGRWGTLHHGKWNGLIADLVNKKTDMVLTSLIINSDREAVVDFSVPFMETGVAIVVAKRTGIISPTAFLEPFDTASWMLVGAVAIQAATFSIFFFEWLSPSGFDCSTGQNSKRAPQNRFSLCRTYWIVWAVLFQASVHVDSPRGFTARFMTNMWAMFAVVFLAIYTANLAAFMITREEFHELSGLDDPRISRPLTIRPPLKFGTVPWSHTDATLAKYFSEPHAYMAQFNRSTVSAGVSGVLTGELDAFIYDGTVLDYLVSQDEDCRLLTVGAWYAMSGYGLAFARNSKYLSMFNKRLLDLRSNGDLERLRRYWMTGTCKPNKQEHKSSDPLALEQFLSAFLLLMAGILLAALLLLLEHVYFRYMRGHLAASTVGPCCALVSLSMGQSLSFHGAVVEAAARGFGTGPGGRGHCRSAACAAQ

>Hnub-iGluR5

MNNENYHYIFTSFDIELFDLEDFYYNRVNMSGWRLVDRDSDKVKDTLLVMEKFHPIGASILTGGHIKTEPALLYDAVQVLALALAATKEVNPTNASCDEETPWSHGKAVMENINKIHAHGLTGPIQFKNGVRTNFTMQLMRLVGGEKGGTVVSGHWNPDAGLTITDPAAYKRDPPPNVTLTIVTVEEKPYVMVKEGWNLQGNARFEGFCIDLLARVAAKAGFAYRLRLVPDNMYGARDPDTGQWNGIVRELMDRKADIAVASMTINYAREAVIDFTKPFMNLGIGILFKVPTSQPTRLFSFLNPLAIEIWLYVLAAYILVSFTLFVMARFSPYEWSSSTHVCGHETKLLTNQFSVCNSLWFITGTFLRQGSGLNPKATSTRIVGGIWWFFTLIILSSYTANLAAFLTVERTVLPIQSAADLAAQTSVQYGTLNGGSTMTFFRDSNIDIYQKMWQHMSTTSPPALVSSYEEGVRRVLQGNYAFLMESTMLDHRVQRDCNLTQIGGLLDSKGYGIATWKGSPWRDRISLAILELQEKGVIQILYDKWWKNTGDVCNRDGKDSKANPLGVQNIGGVFVTLLCGLALAIMVAILEFCWHTKKNASQGRQSLCSE

>Hnub-iGluR6

MDWDRFSFLYEDEGSFIRLQEVINTWENDKKPMLFKRLIPGGDNKETFKHVFKVARLSYHILDCKTENILGYLEEITQVENYTAYQNILLTSLDAYTVNLDSIDIEGNVSTWHLTTKNEAWTDNRISSPKKVETHLQVDALNHLEKSIKVLLTKFDGQQMFQDPPEFCYKTEPWAMGSELRDALLQTSIKGLTGNVQFDKNGKRIDFVLHYSKLNNESKFVHAGSWNSTTDQISDLYPYSHQSVKSKIEFRVVTKKEKPYYMISPNDTVARGYVIDLMNAIFENIREQQNLDFTYNIDVGPGDKVGNPIEGSRRWDGLIGELLEHKAHFAVCDLTITSERNAVVDFSIPFMSLGISMLFKVDPAPEPDMFSFVNPLSTDVWLYLAVVYILTSLVLLICARMSQEDWVNPHPCNRDPEKLENIWSLYNCMWLTMGSIMTQGCDILPRGAGSRWIAGTWWFFAMIVTASYTANMSTFISNNRRSNNIENVKDLSE

>Hnub-iGluR7

MRATNLLQMASNRLTVSIRELSGKDYRDVLINAKKNSYNNFVVDCPSKKLEQFLRHAQQVGLMADEHSYIFLSLDLFNTNLTPYRYGGVNMTGFQIIKQVKDEIETNFAAQFEDIKIKQFLIFDAVKVFYEALKMINMTIESRVDCINFQSWNYGSSLLNFMKTNKINGITGPLVFDAFGQRSDVFMNVLELTPAGGQLMGEWKVNNLTITRPFMTIPDISEESIMKNQTFKILVEMVEPYCYLKESATTLEGNARYEGFAIELFEKLADMLGFTCEFEVTNMSYGGWDKDLNVSYGVVREIETEKADFAIFDFTITAERQKVIDFLTPFMSLGISILYKEPSKQ

2. Cydia fagiglandana

>CfagOR1

MTSKRVFEKLKKRFADNDVDSPLNFKYVNQLQVIMSSIGSWPHKQFGRHRLHTILSLYNLLLQALCALMFILGVLYWRQRMHTMSFFDSGHIFLCMIFDLLVLIRLLVARTMKYQETIKDYLLEFHLFYFKNRSPYAAKVHTQIHTISGMFSFYVICQMAHGVLLFVLMPWYNNFKNGMFGKNRPENSTFENSAYYYLPDACYTTLKGYWILFAFNSFTSYIVTIGLFEFDLLISLMVFQIWGHLKILRHNLLNMPPPENSKNGMYSVEENIKIRALLKEIYEHHNLIIKFVNRCSNAFSEYLFTFYLFMQFITCILLLEVSSFTADALAKYGPLTIGMHQQLIQVSILFEMLNTKSNQLIDAIYAIPWEHMDTRNRKTVLFLIRRIQKPVSLKAGGMVPVGVNTMMAVLKGSVTYYMMLKAFAGER*

>CfagOR2.1

MHTQIHNISVIFTLYLVGQMICGLTLFIFMPWYNNYNNGMFGPDRPANRTFEQAVYYHCFTEDVYTTIKGYWLLFLFNLPTSYNTIIGVVVFDLLLCLIVFQIWGHLKILKHSLSSIIPKDGMYSTEENMRVREILKEIIEHHKLIIKFVDKCSDAFSEELFAFYLLMQLLTCTLLLEVSSLTADALAKYGPLTFGMHLQLIQVSILFEMISTKSEQLIDAVYAMPWQCMDASNRKTVMVLLQRAQTPITLKAAKMVPVGLRTMAAVLKTSFSYYMMLNAIAGER

>CfagOR2.2

MVLKRLYEVLKKRFDDGYVNSPLDFKYVTQLKFVMTTIGSWPYKQFGRNQLAPIMSMYNAFLILFGTIMGALAFNYIRVNRVKLSFFDLGHNILCWIFCILYLQRIVTARTRKYQETIKDYLLDFNLFYFKGRSPYAAKVHAQIHIISGMFTIYVMWEMFIGVSLFIFMPWFNNYSRGMFGENRPENSTFEHSVYFYFPDTVYSTEEGYWMLFIFNIPISYVTTIGLCVFDLLLCLIVFQIWGHLRILKHNLQNIPLPENNIMFSVEENNNIRMLLKDNILHHNFIIKFVDRCSDAFSEYLFAFYLFMQFITCI

>CfagOR3

MLSHQNDDSQITSPKDLGYIKQVATSLSRVASWPLMESKNKKYTFAVKWNITCFLFMSYIFIFQIWYVRNNISTTSFVVIGHTYITLAMNTICLQRLTMPWMAEYRQVIKEFLDKIHLFHQKDNSEYANKIYKYIEKICKIFTVFVHFQLYGGIALFNITPAYKNFKAGMYSSNKPVNATYETAVYLALPFDCFTDIKGHMFLSLMSLVSTSIGSTSFCLWHLLLSLIVFHIWGNLKILKHNLDNFPKPASQMTSIGIPWYTDEESKNISTLIVELVNHHRNIMDFMAKTSSAYSFFLLLNFAFYQIVGCIILLECSKLDSEALGNYGPLTVVLFQQLIQISVTFELLGSQSEKLIDAVYGLPWECMDMKNRKLVLFFLQNVQEPINLKACGMVPVGVQTMAAILKACCSYFIMLRTVTASEEMT*

>CfagOR4

MQSSVTKNVFKLGYMRKVRAVLNTIAQWPNATFGKLHRRARILEAYKYVIPVAQLLALIPVLLYLKKYITTINFIDKGQVYCNIFLTALFLQRASLPFQKGYQNTVKKFCLEFHLNHYEYKSEFAKKETRKVNTLCKYVTGIILCQLYAGILLYNFVPLTLNIYMGMFSTPVPENKTFVHSVDYLLPFDAYTDARGYLVIFFFNWFPSYNIPTAMCSYDLLIFIMVFHISGHLNILSHSLKTFPQPKVVSTEQTRLYNIQEDNKEMFRALKGAIDYHRVIKKFMTEMTETFDVTLCSYLAFHQVMCCLMLLECSTMDPKALGKYGILGGVIFQQLIQTSVVFELIHSKTETLGDQVYSIAWEDMDIKNKKLFLMFLNHVQKPFGMKACGIVNVGVLTMSAILRTSFSYFIMLRTLSNQEK*

>CfagOR5.1

MAPPRVFRSLKRWFDDSDAKYPLEFNYVRQLIFLLSSVGSWPHNQFGRDRLHFVMSIYNLILIGVAITISSAASAYIWINRETISFSFMGHVILCILLETLYLQRITTSRTKQYGEIVKDLLDEFHLFYFQNRSQYAAKMYKQIQVISKIFTIYVSCHILCGISLFAFMPWYNNYKSGMFGADPPANKTFEHALYFYCFTDKFYTNVNFYWILFFFNIPTSFHTSSGILTFDLLLSLTVFQILGHLKIMKHDLSCIPSTADIYSPEENVRVRETLKGIIDHHNIIIKFVDKCSEAFSTYLFMFYMLMQLLTIVVTLEVTAFTADALAKYGPLTVAIYQPLIQISILFEMISTQSEKLIDAIYDIPWECMDTSNRRTVMFFLLRAQEPVTLKAAKMVPVGVMTMTAVLKTTFSYYMLLNAIAESAEQ*

>CfagOR5.2

MQRLFTARTDKYQQIIKSYLLEFHLFYFKNRSPYATKIYKQMQIVSRIFTIIVPCIILSGVTLFNFTPWYANYRSGMFGPDRPPNTTFQHAVYFHCFTEDVYTTLNAYWILFIFNIPISIHTTCGLLAFDLLLCLIVLQIMGHLKIMKHSLSNITRKADMYSAEENMRVRESLKDIVVHHNIIIKFVNKCSDTYSEYLFAFYSLMQILSIISLLEVAAFTPEALAKYGPLTYALHQQLILVSILFEMISSRSTELIDAIYDIPWECMDTRNRRTAMVLLMRAQTPVTLKAAKMVPVGVTTMSAVLKTSASYYMALNAVAKERQMDQR

>CfagOR6

METTIQTSEPKIFSLNYMKQLRFTLETIGQWPNRALGDFSRRAILLSMYHKFLIGTFCFTEVLAFLYMRKHRATIRFIDMGQIYTNLFLTGLFLQRAALPFQKNYKECVKKFVFEFHLMHHEHISDFALKESRKVNKICQIATKVIYLQLACGMLAYNLSPLFRNYYEGMFASELPENKSFVHSVDYLLPFDAYRSFTGYLVVFTWNWFPTYNIPTAMGIYDLLVFVMVFHMVGHMNILYNSLKEFPRPKEGQSEDPLPSTREYNEEIFGQLKNVIRHYQMIKEFMGDMTAAFDLTLCCYLAFHQVMCCLMLLECSTLEPEALVKYGLLAAVIFQQLIQTSVAFELIKSKSDSLGDEVYAVPWEYMDVKNRRILLLFLRNVQDPLGLKACGMVPVGVLTMSTIIRTSFSYYLMLATFD*

>CfagOR7

MTTQISFRERSLDLDYMRAIRLYLDTAGHWPNEQFGPKTMRTRILYIYHKLMLTILVYAEISALCLIRVRMTTLDFIDLGQDYLSICISFVMITRMTLILQGKFCSLIKNFVSKFHLLEHKHESELAAKEYRKVDKMCRIATLFILLQCLFGQVMFNAVPIYVNVQAGLFTDRAHRPPNVTFVHSVNYYFIIDQYEDAIGYTMVSFLNAYISYICGVSFCGLDLLIYIIVFHILGHFNILVAKMRNFPTPLSCPDKSEESSEREYNEEAFKILKNLIQHDQLIKEFLNDTSNTFDITLCLCLLFHVISGCISLLAISPMTAEALTRYGPLIFILYTQLIQMSVIFELINSKVRLRNLSNTLSDEVYALPWEVMDERNRKTVLLFLVNVQQPSSLKAGGLVPVGVLTMSQIIRNSFSYFLMLRTLGNV*

>CfagOR8

MDTGNTDVFDVPYMRMIRFSLNTIAQWPYNTFGPKTMRTRILSLYHYVMISVSTYLEISCVFYVRYNQDKEFIVLGHDYFNILMGIVIIQRMTLSFQKRYCLLVKNFVSKFHLMNHQYKSEFAAMELRRITKICNIAAVIIHIQIIFSMMFFNMVPLWKNIHAGMFSDHRPENGTFVHSGNYLSFVDQYTDTRGYFLVFFLNFYPSYNAAVTFLCMDLLIFIMVFHIAGHLNILVHDLRYFPRPNEMEQCLGTGTRKYNEEVFVRLKDLIDRDQAIKEFMINISETFGISLCIYLAFHQVTGCVLLLECSQMTPEALGNYGFLTMMMFQQLIQTSVIFEFISTKSDTLADEVYCLPWELMDARNRKAVLLYLKNVQPPRALKAGGVVSVGVLTMSTIIKTSCSYFLMLKTLTVEE*

>CfagOR10

MVSVQKIISLAKRLEDPKHPLLGPNLKGLYVYGLWQCGSKFRNTCYNVIHFCAFLFVISQIMELWIIRHDYLEALHNLSLTALGMVCIFKAVSYVMWQSDWKKLVEGISAEEISQSESLNDACIDLKQNYTNYARIVTYFYWNVVVSTNITMVSAPFLKYATSSEYREQISNGTEPLPQIFSSWFPFDKTTMPGYSLAIFIHILINIHGGGVIALYDSNAVAVMVFIRGQLAMLREKCKHIFDDYDLVNEEIILGRIKECHRHHNFILRQSSLFNSLLSPVMFLYVLVCSGMICCSVIQFTSEEATAAQKIWVLQYTTALVSQLFLYCWHSNEVVVECQNVDGGVYESEWWKGDARVRRQLAMLGGKLTHTVVFSAGPFATLCVPTFIDVIKGSYSFFTLLTQMQE*

>CfagOR11

MSTKQSDSFTQNRFFWTIFGLWPGKIPNEYYKYFSFTYLMITFVAYEALLTLNLFYTPLRIDSLIREIIFFFTEIAIGTKACMVIFKRKKIAAIFDMLDCEEFKGSDETGREIVRKYHQYYKSYLKTFTILCNFTYFAQVLFPVFGYWIFGNNLELPICKYYFLSDETRNQYFTLIYIYESYGMYNHMMYNVNNDTLIAGYMALAIGQVKVLQHDLENLKVDKSDDSSEKMRDLKQQQKLRKILHHYGLLIEYVNQFQNVIGGAMFVQFGIGSAIICASMLTLLLPATVESYMFTVAYFMALNLEVFTPAWLGTQLTYESHDLMTAAYSGEWLPRSERYKRSLKLFVERAKQPIIITGLELFPLSLPTYISIMKTAYSCFALIRIFHNRQE*

>CfagOR12

MAPKLSDSAKSTPSFKQSDRFKQPNNSKQSDGFKQSDSFKQNRFCWSVFGLWPGKIPEKYYKFFSFAYLMISYVVYNALLTLNLYHTPRRIETLIREIIFTFTEIAVGSKLSMILFKRNKIAAIFEMIDREEFKGNDDIAREIVVKHNDYYKKYLLLNTVLSNFTYFSQVLFPVFGFWFFGNTLDLPICKYYFLSDETRDYYFTSIFLYQSFFMYGHMMYNVNIDTLIAGFMVLAIGQVKVLCHDLENLKTDKSVGGQSIIDLQQQYKLRKILNHYELLLEYCDRFQDVIGRTMFVQYGIGSGIICVIMCGLLLPSSLETQMFMVGYFMAMNLQIFVPAWLGTQLTYESEALTTAAYKSEWLPRSERYKSSIKLMMERAKNPVIITGLKIFPLSLATYIQIMKTAYSCFALLRIIQDRQEQTGA*

>CfagOR13

MRPLRQIDCFKVNMKCWKLLAVWPPNDMQSYYRCYQMFFTAFILLNNLLATVNFIFLPRQLDMFIDEMIFYFTELAVTSKLLTFLFMREKILKILSILESDMFQPESENGLKAIDKAKKFNVRYFKIVAAVSATAHFSHIVPPILLHFILHVKLELPVCNFSFLSDDTKQKFIYPLYEFQALYMHSQVLFNISIDTFVLGILIYAIAQLDILDDNLRRVTVKNQIGSTRADDSIQRAEKENALKKLNDSIIHYGEVGQFCDLVQDVFSITLFVQFGVASCIICVVLFRFTLPAPLQYFVFLGSYMIIMILQILVPCWFGTRIQDKSQQLSQAVYDCDWTAKSRYFKSSLRLFVERANKPLSITAGKMFPLSLTTFTSIMNSSYSFFTLLRHMQSRQN*

>CfagOR14

MDRIKAFWTSDSTTILLPVFDDVEFKPFRETYRIITFNMVVGMLYPTPETAVCRLVGIVVVLISITPAQLIGLLDVWHSWQRGDIINIVRHITVLGPFLAVIFKMMLFYYTRNEAWCVIKKIDADHARYNMIPESHKEIARRHIQNTQYYSEKCWAITVAVTVLTFPFTAVVLNFYNFAFKEEPVKYMIHDLEKPFSPPEDRFSSPYFEIMFGYMAYCSLWYIISFTGFDAFFGITINHACMKLELACKIMEDAMLEKDRDSRQRRMKEVISEHIDFYSMVELIQETFNLWLGLIVIATMCQICNCMYQIIEGYGIDPKYIIFILGTIAHIYLPCRYAAKLQVSALDCATRLYCCGWEHVNDERAVKMVAFMIARAQVPLKITAFNMFYFDMELFVSILQTSYSLFTLLRS*

>CfagOR16

MNIGDLYFSRAKFVMSFLGVWMPPRNESIMHKCYRFFMLSLQYSFLVFQVIYICQVMGDLGEISESSFLLLTHASLCFKITVFHVNIESFRELLAQMNSETFMPQTEGHDKILKLQASRIKRLLLAFMISSQATISLFALSSLFDDANRKFPFKLWMPVSPESSPQYELGFMFQFVTLSMSAFMYFGVDSVCLSMVIFGCAEIDIIKEKIMNVKPIAERRVNRSVTAKNILDEHYKILVECVAHHQAIVKFIEQVEDTYHLYLLFQLSVSVGLICMSALRLLAVDWKSVEFMSVVMYLLVMVSQLFVYCWTGHELTATSSELHTVMYECCWYEQDVRFMRVLLITM

>CfagOR18

MAVRTVNNVTLFLNRPRNILLYLGIWLKPANYVSLYVAYAIIVMFTQYSFVFFEFIYIALAWGDMDAVTEASFLLFTQASVCYKVTRFMIKKNNLVFLLSFMEEEVFQAQNERHVRCLLNQSIMIKRLCLFFLGSALTTCTLWGLMPIVDSTGGERIFPFLIWMPVGPEKSPQYELGYFYQMVAIYISAFLFIAVDSVALSMIMFGCAQLEIIMDKVKQIKRVSMSGKLKRQDREQLIQENQVLFVECLKHHQAVIRFIESVEDTYHANIFFQLSGSVAIICIIGLRITATTPGSVQFISMLNYMVTMLSQLFLYCWCGNELTIRSELLREVMYLCPWHEQSNSFRRLLWVAMERMKRPIIFKAGHYIPLSRPTFVAILRSSYSYFAVLNQTRNKEK*

>CfagOR19

MKNYYILKDLCRKIYLAGAGDFWFEEGEISKGKSLRYQLLCILLFSIYISMTVLEIIGVFFGDMPDDERSDCTTFAVSHTIVLGKMFSVIYNRKRVKELNRKLVEICKDHEDEHRLAENYKIIKINVTAYAVSVYGSFVFFLYEGIRKMMTGSHFITIVTYWPYYEDNSAIAITFRFFTTGVLVVMMATMICIDSFAMVTLIMYKYKFITLRYYLEGLREQFDINNYAGNEEYATDQLHSGLIEGIKMHSNLIRLSKDIDRCVGAVLALQVCLSSGSAVSLLLQLALSKNITVGAQLKIILFVGALYFLLALFLCNAGEITYQASLLSDSIFYCGWHASSMRRDIRRLVLFSCAAAQRPIVMKAFNMLELTYGTFIQVVRGTYSVFALIYAQNESTTE*

>CfagOR20

MNQSKTDCMKYKSFNETFKFCSFALAFGLIYPNRKNVCLRSTLFSFVLLFNCGTLFWFMWYTVKCLWELDIYNSTRNITVGVIILLFVFKTYYVNFKTDMFAFLLEQITKDLLKGNNMDADYQEIYDYYIKEGMFGQKCYVWIPLILSSIFPTYAGVSMIYGSLTSDDFKKVMLHEMDLKYLEDKQYDFPYFELVFAYYFLGIYSMVPNFAGFDGSFCIATSHLRMKIKLMTHSVQKAFTDSKDILELRAKLKTCVKDHQEALEFYDLIQRLYGGWLFAVFLLTSFLISCNLYQIYLTGIDPRYTLFAATGVFHMYMPCYFASCLIELGEQSCTDVYCAKWESWADPTATKFLIFIMARAQKRLLLHGMGIVFFNMESFVSLMQTSYSFFTLITSK*

>CfagOR25

MLGSLLELSDDFFAYNLKYLFIVGLWPDDPWAKAHPTLYKMYEYTTHVMSIIFLIVTGIGTYQIKDDVVLLMTNLDKCLVAYNFVAKVVIFVVKRRQVEILIFEIIDSGDQLTEERKKMMLMMIVIVTGLSTSIVGAFSALALYHNELSVEAWMPFDPMESKMNLLTASQLLAISFVVPVIWRAIAMQGTVCSLVMYLCDQLVELQDRIRSLEFTSTTERAVREEFKDIVKKHVRLMRYTQDMNKIFEEYFLIQNLAVTLELCLNALMATMVGFEQKTLLATFFAFLCVALMNAYIYCYLGNEMITQSENLALAAYESSWISWPLDLQKDLVILLRVAQKPLYMSAGGMVAMSIQTYSQTLYNGYSIFAVLNDVVA*

>CfagOR26

MAEYEGELLSYLSLTPHLKVLRSCGIFPLDPSSSNLKKKLHGFYIYIGFSLVILYTLLQIIHVFQVRADIEKVMDAMFLLLTFLDCIFKQVMFMKKPHKILEMLNIMKGPSFNQGLDEHRPLLVRTINHAQFLLRLFNKICIVTCFLWITLPVYLHLKKETVEFTIWVPFDTNENSKFYIVIGYVWMQTTWLGLNNSTMDIFIVYLFAQIKTQICILRLNLENLVSRCQEESKSSFHSFTEHLEWRFRGIIYHYNQIIKFSKINEEIFSSAILFQFLISGWIICTTAYRTINMKPLSGEFVSMILYMICILSELFLFCFYGNEVAHESRRLMESAYCMQWEEMPVKYRRYLIIFMERIKCSILPKAGKIVPLSINTFVQIVKTSYTFYTFLSNSNAN*

>CfagOR27

MLRKYVARLEDPNHPLLGPTLWGLQSWGMWQPNSGPSRIIYNLIHLAAILFVVSQYVELWIIRADLELALRNLSVTMLSSVCVVKAGTFVVWQTYWQDIIQFVSTLERSQLEKKDKTTCTIIEGYTKYSRNVTCFYWGLVTATVFTVILAPLGVFLSSSEQRELMFNGTIPFPEIMSSWVPFDKTKGFGYWFQIVEHSVICFYGGGIVANYDANTVALMSFFCGQLEILVANSKRLFSEDNKLVSYSEAMDRIKQCHQHHLSLIKYSKILNSLLSPVMFLYVVICSLMICASAVLLTKEGTTTMQRMWVAEYLAALIAQLFLYCWHSNQVYFMSESVDRGIYESEWWRCGVRLRRCVVLLGGQLRKTIIFQAGPFTDLTVATFVAILKGSYSYYTLLSSNES*

>CfagOR28

MDTLARVMFLLLCHVTSIAKQIIFMARASRIAKLVQDLDDMAYNPKETTRKNLLIERAQGASRLGTAYAGTAAVTCLLWTIFPLLARLGGARVIFALWIPFDYYSWPEFLIVLLYTHYVTSLVGIANTTMDAFIATILGQCKTQLTILKMDFESLAERANERARQTGEQVGVAAAALLVRCIKHHHKICDTSREVQEIFGGAVLLQFGIGGWILCMAAYKIVGLSVASLEFVSMVMFLMCILTELFLYCYYGNEVAVEVSSLCKIFDTITEV

>CfagOR29

MIKEFFQSLEDPNIPLFGPNYWLLSKLGLLLPKNRMERILKIIIHELAFCFVISQYIELYIIRSDMDLVSTNLRISMLSIVVTFKANTFLFWQEDWRQIIDYITEADKFERDNQDEAKGNVINTYTKYCRRVTYFYWVLAFFTSCTVIIAPLMKYCTSEKFREGFHNGTEQFPHILSSWVPFDKEYSPGCWFTVIWHILITYYGGSVAAAYDTSIMVILVYFGGKMNLLRIRCREMLGTEGKGVSDVNADKVVRQLHQIHVLLLKHSRLFNSVLSPVMFCYVVMCSLMICASAFQLTSATSTLQKLLMAEYLIFGVAQLFLFCWHSNDVIYKSEAVMMGPYESDWWAANLKQRNNILIIQGQLQIVHIYTAGPFTDLTLATFINILKGAYSYYTLMRK*

>CfagOR30

MRLNRSHPFIPRDKKWRTQFTAISILSFFCSSFLMYSTFFHDIPCGAYADASKSAIMSIVAITITYKYLIILRYQESIVDLIRIVDEDYELAKGFCEEEQRIVYKYAKRGVKVTQYWFVSACSTSAIFPLKAFVLMGKSYLAGEFQLVPLFEMTYPWILNDYKNVHVVFVMLFGLTLFFDIYATSMYVGFDPIVPIFMLHLCGQLDILNLRISKLFSNTEDSAETRRENLRRIILQLQDIYKFIDIIKTNFTVLYEFMMKTTTFLLPLTAFQITEGENFRLAIYSCGWEKHSDTRVMRTILFMLTRALKPIVISTVFCAICLDTFAQMCREAYSIFNLMNAAWA

>CfagOR32

MIKQIFSNLLSRYIPVWNQENPALANTVLRLIRNTGIWHYQSFGLHWVAKFAIICFISTNLTQVVSLVIHRDDSTRMFETFSVLSFCGMGTLKLFNLYTNRKRWTSIITQVKCLEHEQLHGKLLSSIDSGIEEDYSPQIIAKYTRRHTFISSILLRLYSITAIVFMATPFVEYAVTTDASYYPHILPGWAPLDNVGFAGYFLTLIFEIVASVYCVFVHVAFDCTSVGIMIFICGQFSLLRRKTEDIAGSGKDCMPSTKRDARAHLRIIESHGTHSALCTISKELDTVLRGILGVYFLVATLTVCSVAVRLSSEILSFMQLVSLLQYMAGTLTQLFLYCRYGDAVFHESSFNMGEGPFGAAWWS

>CfagOR35

MSIEGYKRIRNKKISRYSLQYMLKCLEDPEHPSAGPYLSFMNLTGNWHPNMELKSTRFKQMVYYILMTFFFSQYLKCLIGLDLSALLFIIQTAPFHMGTPKTIFFRKDYHLWEKLIDYMSRTELRQLSDEDEEVIDVMDEYIRKSRRVIYPFWFMAICCNISLFTDPYQKNQIVENGTDIYVPLFQFYVPFNQDVPPGYYYSMVIQTILGNIMSSYIISWDSLVISTFIFFAGQLKISRVYCTKVIDPESKERSHENIIKCHRFHTSLKEHQKLFEKLISSVMFIYLIVISINLGSCIIQISNASGDLPAMMAAMLFVFGILVQLLIFYWFSNEVTVESLSVSSGIFESKWITMDAKIQKEVALLQHTTSKRLFFQSRAM*

>CfagOR37

MNADTFSMAPCVGIEPLTSFPQREICIALISIQEITILNTTHSFQTVMLLLLAHTSVMYRLLSDEITTFSTLLTDPRNYDFVKERLPVIIYRHVLILDITKDLRALYSIPMGINFGSNAVCMCFFFFLEPEEYLSFMPIVVYCFIVFSLYCFLGQRLTDGAEMFSQAVYNSGWEMMHIKERRAIVFMLLQSQKEVDLLAADLIPVNMSTFATTCKAIYQFATVFKL

>CfagOR38

MRMLEGLRQFGLERHDFPTLLGNVATLLRMMTLNIDSRNTKRIPFIFYVWMLVSLFCYVYGFPISVFWFIFLRDEEEELLKKIIAFSQLTVCTGSVGMYLHMYWNKNKLNRIIDAYILCDSQVVPYSRMSRNIQVALKSVKKRAFIFWIFAMSQVVIYFMIRPLVLDRNSMKDYQLLWGLDPIVETPNFEISLFVIMMISTVSVFAPLNILVLLMVIVGYSEAHLLALSEELLYLWEDAEIDYQMQSPKNLQIDEFVKRRLENIIKSHAQSIEMFQQVEDLFRNGFAIQFMLNSVSLVACLMGGLQNTYVQTPFTLMLIGMDCFTGQNLMDASLTFEAAVYSCKWERFEKNNMKSVLLMLRNAQKTMQLSAGGVAFLNYACFTTIVKTIYSTYTAFQSSHVDTGETKELTTF*

>CfagOR39

MMETMDKFGLAHCDLPTMMWNVAVMLRMVAVKIEGSAKSIPIFFYLLAAVSIVLYFFNYYASMIVFVLVDCRETGDVLAAIMALSLSITSLIGINKLMYMYIHQDKVQSLVADYISYDRELHWPGTAALMADLMRNVKMRLILFWAVTMGNAFIFNLQPLAMPSEVSRLNLYDKHVVYGLKFILGITNYPIALVNIVICVFFICSVTSCIGGLLIVTTGYSEVRLLALSQEMRDLWSDAHQHYSENFEGDSEGKKAAKELNNYVNYRLQVIVKSHAINIDLIKKLEGIFRNAIAVEFILVTAGLSIDLLGGLEDTFIILPFSLMQVSMDCYLGQKLMDASKVFEDSIYDCKWENFDVKNRKTVLLMLKMSQRTLSLSAGGVATLSFECLMAIYKSVFSAYTALESTMK*

>CfagOR42

MSTSTFDEVFRQIRINLSVMGIQEDKSRPGVTFYIFYAMVFTMVSSELVFFSVNMAPENFLELTGLAPCICVGILSLLKIAALAYKKETVFSLADKLERLSTENLNDPVKTDIVSPDINLLKKLINYYFVLNAVLICVYSFSTPFYILYHYLTTNEEIFILPYAVIMPFSTETWPAWTFVYVFSVLCGFICVLFFTAVDALYFTLTSYVCTIFDVLSNEIISLNQPTGDMLSQIIKKHQNVLELAADLEDIFTLPNFFNVLVGSVEICALGFNLMIGDWNNVPGCMLFLVSVLFQLFMMSVFGEKIIGASIKVGESAFLCDWYKMNLKTQKVLLILMIR

>CfagOR44

MIFSNEYMREKLSFLTPFLPYGVLESWEDLNPRLYHAVHIYWLKFYGLWYNTHPRTSLLFWAHIVYTIVVLWLVCFLPGIGEVVYLLRRRDNIGDIAEGLYLFLSEMYTYIKLSVFWLKRREIMSLLEFLHRDEFKVKEPEHREIIRKSIKRARFVMTYYSSMCVGAVSVGILMPLAEQFEVLPTNVEYPYFDVYKSPAYGIIYIHHIYYKPATCIIDGVMDTILAAFIASAIGQIDVLAFDLRNFNLIVERRRKTLPFAGVNNKSINHMPLVNDQESTQHCQRAVFKDIIKHHNSIIKYVSLIESAFSLASALQLMLSVMVLCLVGIQFLSIEEPSSHPIQIAWMAIYLTCMLIEVFIICWFGDELIWKSWELHQAAFDSPWPSTDSKTAMFIVIFMERCKRPLRVTAGKIFTLSLDTYTNLINWSYKAFAVMRNVKK*

>CfagOR47

MSKIVNSDRPRRYFGVHYRLLRFLGLGWWHHPDEGDFRNFPSWYLYYSILTQVVWVAGFVGLETIDPFVGEKDIDRFMFSLSFVITHDLTCIKLYLFFFKNRAIQEIVRTIEIDVYDYYQNVDKNRRTIRITKIMTASFVFFGWITIGNTNVYGTIMDLRWKKEVALLNDSAVKPPRTLPQPIYIPWTYQSDESYIATFVLETVGLLWTGHIVMTIDTFIGSLILHMSSQFSILQEAFMTAYERALSQLISDMPLDTQDESNNENIDILKDRLDEIEAKVKSFYTDVQIESAIEKSVKTCLRQHQLLISCVEKFRVTYSYGFMTQLLSSMAAICVVMVQVSQDASSFKSIRLVTSLAFFMAMIIQLAIQCFTANELTLQAECVSDAIMQSKWERMSPRVRRYLLIAMMRAQRPLRLSAAGFAYMDNRCFLAIMKAAYSYYAVLSQKEV*

>CfagOR48

MQSLKSRIGIQKTATKEIYTFETNTTVSLFYKICAIVFLGCGTNLMFDDLKLPRKVVKICRIVSKVFGLLVLMLVCSASIASFTQHNLDMMHKAKLWLFGPPLVALNAMQLNCVLRKNQIKKLSYTIAVVLRGMFTVDELEVEMIKKTWRLSIAFALLLNGLFISTGIVNGYVASTTNETFTVIIPAWPDLNERSLTASISRIVHYCLWYLVIMRVCSVYFIILAIIIGLQFQFSIMCNYFHSLNSIFAGEDSHEEKQRKYEDAFKFGIKMHSLTLWCIDQTQVTCGVAFSSQFITNVVTLISLMVQFMYIERTFGNCLPIFLFVGVTLTATGIFMWNAGDVTFEATKLHTAMFHSGWHNCRGQASVRVRKLLTIAIRQAQDPVTIKGFGILELSYESYISMVKLAYSIFSVLSQK*

>CfagOR49

MIKCILKKLENPKRPLLGPNVKALQFWGLLLPENVTMKYVYICLHISIIYFTATEYVDIWFIKSDMNMLLENLKITMLASVSVIKVSTFLIWQTSWRDIIDYVTEADLNQRKTTDETNLTIIKKNTKYSRKITYLYWSLMYTTVVIVMVQPIIKYVFSQTYRDNVKSGEESYIQVVSSWVPFDKSKVIGYLGACAFQSYAAIYGGGWITSFDTNAIVTMVFFKGELQLLRRDSAEIFGTENNPVSREEAEKRFKECHSRHVNLIKYSRLFDSCLSPIMLFYMFVCSVMLCVTAYQIRFGTSLMQTILQVEYLVFGVSQLFMYCWHSNDVMHISQEVIHGPYESRWWSQNALRKDLVIILGQYRKEIVFSAGPFTNLTLPTFISILKGAYSYYTLLTNSQSDI*

>CfagOR54

MYQQIFNTLVYFFSSHPGDTAMKNSDCLASSIAVMKYTGVWMPDNLTHGKRMAYMVFRCVTQTVLFFFIILAEIAYVYKHRHDSERMVDAAVLLLSHLVQAVKLMTIIVRQERIKRLIALGDGPAFTHTEPKLKVQLERAVKLTGLIGNLLLWSAGVTAIFWFVVPALKDVLTLPLKITFPFDISGQYIFAVMYAYTSLSVVTCGVGDAAENFLVSGVLTLASTQVGLLHEQLLDIKADGKDGCYKKAVLCVKFHQRIVEYVEEIANIFSLPIFCQCVTSSIVVCMTVYKITITKEPVEMVTMIFYLMCVMMELMMYCYPADVLLNKSLQVSDAAYPEWSGNVKTAQVLLLTALRAQRALVVNAGGMFKISLPTAAAVVQTSYTYYALLQQKLKKE*

>CfagOR57

MTTTYSAFEAFRPHFNALAYVAYFKIVPKPSSGLKHTLHTAYRGFVWFLVTIYNLQHVIRVIQARHSTEQVVNTLFVLLTTLNTLGKQLAFNTRVERMDRLIATIEGPLFTACNAYDLKVMRRHAWIMSRMLTMYHGSIYLCGAMWGISPLVTKLSGEVELTGYFPFDTSDWMGFGIAVAFNTIVITLQGYGHVTMDCTIVSFHAQTKTQLQILRNSLEHLVDPVGDTGREICVRSTVYKDIEDPAFGVVLKKRITRCVEHYKLIVWFHNEVEALFAEALMFQFFVVAWVICMTMYKIVGLSLLSAEFFSMFVYLGCMMGQLFIYCYYGTQVKAESEFINDSIYRCDWVSLSPQFRALLLIQMSRCMRPVAPRIALIIPMSLETYISVLRSSYTLFTFLERK*

>CfagOR58

MPRIKKASRNMTTIKSNRTYVLFHTICKIAFLSSGTNFWFEDMDFPAKLINIYNAIAPVLEVIVALFIMSHCGAFWTQPNLNEKQSNDRMLLTCVNGLTYLVYVNILYYKREIRELVMTLAVRLKEVCNDGTIEKMMLRTTYRYISALVFICSSAMISYGLGSGIQALTTNATFTNMIPVWPDVEDRRIIAGAGRVVHYIAWLFLLARIIAIYMLILTITISIAHQFKHLCKYFVNLNDIFEGSGSQEEKERKFENAFKVGVKMHAITLWCTRQIQLTAGMAFSGQVIINVCSLGLLMIQMMSTERTLVAMMQIVFMALLILIGTGLFLWNAGDITIEASRLPAAIFHSGWHNCSRQSSVRVRKLVTIAMTQAQDRVVIKGLGLIELSYESYVAIVKSSYSLFSVIY*

>CfagOR59

MNVGGVLFYNVTPICVYLYQLWQGQDAAVGFVWVSWYPFDKYKPINHVFVYIFEMFAGQTCVWIMICTDLLFSGLASHIAMLLRLLHKRLEKLAETEKSQEEYYQEIVANIKLHQRLIRYCNDLEEAFTIVNLINVVFSSLNICCVVFVIVLLEPFMAVSNKLFLGSALIQIGMLCWYADDIFHCNADVALAVYNSGWYRTDPRCRRALIFLIRRAQKPVAFTAMKFTNLSLVTYSSILTRSYSYFALLYTMYNDSY

>CfagOR61

MSEKEEFLAGMEYLSVITSKIFLYPFLARSKTKLLCYHLICFLIFFASFQQFVFLCVSKLNSFLDIVNIAPNIGVCAMSVTKYIKVNSNKELYNQIFVHFRTDMWDIISEKCPENLKILKRYQKIINFITIWFVYYVVPLILIVTSFPILIMYYDNMVLGKELEHRYPFEAWYPFDKVKWYYAAYAWESFITGLVVCIYTFSDLINVSYIAYICLELKLLGTHLKELIGAEDIKQLKRSQNATAIHYKIRQKLRGIIIKHNFIANISSQLDIVFGDIMLVNYTFGSVFICLTAFTFTVTNELYSTLRCFFFLISLVISMLNQCIIGQCVSDHSEQLAKALYDSKWTYGDRQTRQLVLMLIMRMQKPFQLTAKGYIAMNLDTFTTVSCIVVYKKSYGLRISISGLHNSINKCIK*

>CfagOR62

MVLLTSLWRAITHTKALEESSGEMETTFFETVYRITYIAGFSNSDHGFFYKLYSNTVKLMIATFMVGEVWYMITFASSLDSVIEQLNATLIQVMALFRYKYMRMHEHVYKNLATSMQLSNLDTSTPARKALVDYWMEKSETYLKLMLGLGSLTLAVWYVFPLVDDIEYNLTVNLWLGVDWRRPSRYPIVYIIHIVAFHYTAFFIMVNDVIMQAHLIHLICQYTVLADCFENILDDCEKDFKGLSRDQLIRDSSFREVYISRLGLLVEQHKKILMHTMELRKIISPPMLGQVAASSLQICFAGYQVAMTLTVSFIKFSMSLLFLGYNLFELFVICRWCDEIKIQSENISNALYCSGWECGVATMAGVRARLMLVATRASKPIVLTAGGITELSLNSYSNLVKTSYSALTVLLGMRHE*

>CfagOR63

MLLDISITTEAGVYCIVLIYKLLILTCTQLDKAHYHCLLRFLREDFRYVCAEGAKYRERFFENQLETWKVSLCSVIFTFGIAVGMSSFALLSLFYYLMMRTPGDDSQRPLLVPFWFWDLDFGKTPIYEIALNFSNFCFVTYAYNYVFMIQTQVVWVRQIATKADLVIWAIEDLLQDINPATNEEEKAHYAELIKYRMREIVSQHHSMYTLMEAYAGVYKKLLMYEQKLCGPVVCLTAYCTAEKLDEGEFNAILVLLCGATVTLVYIPCYLCTFLGLKVRSVSDACWNISFWNAGREIRPYLVLIMQRSLRPLPLQAPGFEEISIQTFSTKMTNAYSLFNMLRQTSI

>CfagOR64

MKTLISTAKAFLYKKDFEWDKEITLQNFHPQLQIFFAINGVFFNNRESNIRFIFPVLSSLITLVAVAFQIFFIWHGISINDYGFATECFCYFFILGSVGIVYSSVLLNRLKVFKLLHNMNKDFLFICKLKAEYRDTFLTGQLLIWRLCWSWIGFIIFVSALYVSNTMLYLLYQSTLATQDEHMIRPLIFPMWLPEDDPYRTPNYEIFLSLEVVLIYVVVLTFGLYVYILFHLLLHYYNLMDVILIALEELFDGLDESVVALPREDPRRVAVQNELNIRMAQIVRWHLSVF

>CfagOR65

MTLLTIFRDIKSFVNKDDYDLERPDVTLQNFHPQLEIFFAIKGIFFNNHQSKKRFIWPALSSFMANVATGFELMFIWRGLTIKDYAMATESFAYLIILGSVNLTYLGVLAHRTTILKLLGEMSKDFRYICNLAPNYRKCFLEGQLLIWKLTMTWGIFIIIVAILYLLNTFLLLIYQSLFATLDEHYVRPFIFPMWLPHDDPHRSPNYEMILIFHVALIFVAMASFGLYVPLSLHLFMHYYKLLDMILIAIDELFDELDESVVTLHVADQRLLDVKAELSRRMGRIVTWHQSALDSVGAITSIYGPMLVYQVMFSSVIICLMAYQVAIQLAEGKFNYLFALLLLGAVVQLWIPCCIGTLMQTKV

>CfagOR66

MESRKYRKNKTTEFLHKLDKIVFACSSMNYWVDHNDLPEVYLNAYKRIMKVINAGIVLFLVGELASFFTQNNLTENQKSDRVMQTLSQNILYSVALSLDHHKETVTEILFTLAVGLKKDFNDLETEILMLKRTKIYACTFVLLCFNSVLFYGVKGLSKVLFSDETFITMITAWPDVHDRSLLAGVCRVGVYVLCWLWMARVTAAYLIVFAVTISLSHQYMNLQMYFKSLAGVFKERISQSEKEEKYERALKLGIRLHATTIWCTQQVQRTCGNVYNGHIIVNICVMVQLMSQFKDSDRSLSHVLAILATFMSMLFSTGFIMWSAGDITVEAGNLPTAIFMSGWQNCTNMSSYRIRRLVLIAMIQSQKPVIIKSFGFIEISYQSYVSIVKTSYSIFSVLY*

>CfagOR67

MEHLKDFASEFSKPFAICFDLLAKSNISIYNENKIRGKLRVFALVVFYVTFYFSLVVSFKKVLTGELGFYELANLLPIFIVATQGAMKGTVIVSNLSKAKIIIDDLGSMWRTTGLTKTQLMKKGVMLKRLNLCNAVFYWMNIIGTWQYILVPLFETLFRNFVLGQDKLLFPFICSLPYDAKRNWMVYLGTYFWESYSMLHLIYMYLGVEFLMITLCSHLATEFELLREEMLHAKPILEHTENTSYKDYIGATLSCSNNEDVDAIDYFEEDITNEIRADEDGPRIQEVIKRHQKLIMLSELLDDIFNRMIFFNLLFATITICFFGFVAKIARDPPEMANNFVGVVASMIPIFNLCYYAELLSGASAGVADSAYHNLWYEGDNRYQKIIIFIIVRSQQPCCLTSMRYAQVTLNTFTTVRILSIKNVCHEKDLISEFKKT*

>CfagOR68

MASLEQLPSSLIDTIIIPVKLFRFIGQEFFDDYEARFKNYCKFTIFVLLSFIFSSGFTLFFIKINETDAGILEIANAIPCFLLVIQSLLKLSLLRKKHRIRCVVYEIAELWPRDIEDREKKEIMNYWIHRNKIVCEAYFRCTRAGLIIYNSINLVIYFVLRILDKNPDYVFPCQLYYPFEMDSVWKYVAVYLMQILATTTIYECSYESCDMFLFTLTIDVSMLFRLLQHDLVNINVGRGQDADESLENLKNIVKRHQNLLKLAEDLDEIFGAIMFNVLIFSSLIICFFGFLGIVIEVKFQQCMYLTAAIVVLFSVFYIMLPGQILSDTSSGVASAAYQTLWYNSDQRFRKIIVIIISRSQKPCKLRAMGYADINLETFYKICGTTWSYLSVVNQMYQNSL*

>CfagOR72

MSEFSNFEPMFQETYKFILDRIKSNQIYIMDEGSWRGRLCWIKLAVNILAAISHTAGVFERIGQGADLVQLSTDLSAALILWQACLLYIQFCLNRKLLKNFIVHMGSNWRIDDQLRPDMVAVKHEYVTTFLRWISVFYKAVNTYLFLYLMPRLAYTAVKHFILKDSVAFVTPFYVKMPFKFDDNFLLYSLVYLADSKILQDVGYLVTFDLLFMNAALHHLRLMFVMLQDDLKHVQEESVEQAENTLRKIIPLHQDLLKLMFELSNAFGAIFIIHLAFFSATMCFFGFAARIHCSPESIKNLLAANIILVCIYTCCYYGQNLTDASVDISQAAYESQWHLKSQEYKKCILIIMLRSQKTQYIKSTSFTDVSLETFTKILNVTWSFLSLITEVYEA*

>CfagORco

MMGKVKSQGLVSDLMPNIKLMQMAGHFLFNYTDETGGMSLLLRKVYASMHAFLIVLNFICMGINMAQYSDEVNELTANTITVLFFAHTIIKLAFFAINSKSFYRTLAVWNQSNSHPLFTESDARYHQLSLDKSRRLLYFICGTTCLSVVSWVTLTFFGESVRLIADKESNDTLTEPAPRLPLKAWYPFNAMSGSMYIIAFVYQIYWLLFSMLIANLMDVMFCSWLIFACEQLQHLKAIMKPLMELSAALDTYRPNTAELFRASSTEKSEKVPEPTDMDIRGIYSTQQDFGMMLRGAGGRLQNFNSPNPNPNGLTQKQEMLARSAIKYWVERHKHVVRLVASIGDTYGTALLFHMLVSTITLTLLAYQATKIDGLNVYAFSTVGYLSYTLGQVFHFCIFGNRLIEESSSVMEAAYSCQWYDGSEEAKTFVQIVCQQCQKAMSISGAKFFTVSLDLFASVLGAVVTYFMVLVQLK*

>CfagGR1

MENDNPRFKMFNPNQNEQIRKRDMYGKNAQLDADENKETAQLPGLQITAQDGELLDKHDSFYITTKSLLVLFQIMGVMPIMRVPKDAQTTKRTTYNWISKATFWAYLVWSLESIIVVKVGKERYENFQKSSNKRFDEVIYNIIFLSILIPHFLLPIASWRHGPQVAIFKNMWTHYQLKYLKITGTPIVFPNLYSLTWGLCFFSWGLSFAVILSQHYLQDDFELWHSLAYYHIIAMLDGFCSLWYINCNAFGTASKGLAQNLHKALEADHPALMLAQYRHLWVDLSHMMQQLGRAYSNMYGIYCMVIFFTTTISLYGALSEILEHGLSYKEMGLFVIVGYCMTLLFIICNEAYHASRKVGHEFQVRLLNVNLGAIDHSTQREVEMFLVAIAKNPPIMNLDGFTNINRELFTANISFMSTYLIVLMQFKLTLLRQGARKAVRSIVKAIFNSTTMLPDEEYEDDE*

>CfagGR2

MDGFLLRHTTAYYHIITMINMNCALWYINCKGIKIASQSLSECFRRDVNVDTSAKLISRYRFLWLNLSELLQSLGNAYARTYSTYCLFMFFNITIAVYGALSEIVDHGFGFSFKEMGLFVDAAYCSTLLFIFADCSHKSTLKVAAGVQDTLLGIDVLAIDRPAQKEIDHFIQAIEMNPAVVSLKGYAHVNRELLTSTISMIAIYLIVLLQFKISLPKTDS

>CfagGR3

MTRSRRGVAISRSSPGSPLAVQTPTWLVPRKKAEMAFYTNNSLFPNQPPIPNGIAAQMDEKSKNKIIFLDVTPNRTPRLPTPNNAIAPIQDNLINPDITRDIIYENIKPVFTLLRIMGVLPLTRPVPGVTQFQPASPSMLYSAVLYFSLIGYLLYLSLNKVQIVRTGAQEGKFEEAVIEYLFTVYLFPMIAVPLLWYETRKIAEVLNGWVEYEIAYKKLSNRVLPVGLYKKALAMSIVIPALSTASVIITHVTMVHFKLLQIIPYVFLEILTYMLGGYWYLLCETLSICAHILAEDFQQALRNIGPAGKVAEYRALWLRLSKLARDTGIANCYTFTFMSLYLFLIITLSIYGLLSKISEGFGVKDIGLALTAFCSIMLLFFICDEAHYASHNVRLNFQKKLLMIELSWMNADALTEVNMFLRATEMNPSQISLGGFFDVNRTLFKSLLATMVTYLVVLLQFQISIPDDSRVQEADDDDDFVNATASVTEAPTTLTTITTLLTTLAKKKKKH*

>CfagGR6

MFKNKKSSKLFFVTETKLTNDKTGALKHYNLKLQPEIFMTPLLTADYNGYQATFQEAMKLTIVIGQFFGLNPVTGVSEIDATKLRFQIQSYRFVYSLLSIIGQCTVVSFCLLKLFTDSNPNLSANSALVFYVTNCITTILFLRVATRWPRLCQLISKTEASDPSIDRTLIKKCRVSCVLVLTMALLEHILSDMSSIAGIIDCQKGKNVYETFVVASCPWIFQYTGYSPYVAIFTQIIGLQFTFNWNFSDVFVICISFYLTSRLEQVNRRIESVFGKHAPSSFWRTLREDYSRITGLVRRVDDVIGSIIFISFANNLFFICLQLLHTLAEGIRRNPSCRLGEPDERPFQGYEQTVYFVYSFGFLIARSLAVSLIASRVHTASREPAHALYFVPSTAYSVEVILLHDVL*

>CfagGR9

MTVFNAVMLAAFILDICLEPGQRIRIGETTLKMFVWSSDMLLMMMIASVAVYMAPNRMYHLVHVLDQLRQVSTELKINSSAGSERTKTITIVCIPLWAASILIADFYSFLGPLLDGKLWYIMCMYGPYYVGNLMGVLVLLQWSCAVLAVHAAVVAVNDELATLRRAKFELEPTTALEDLLRPPKSERNTLIDCFTKPAKSPGDSMTLSQAQATIRRLALTHERISELTRQLNASNGVFLMFVLMSTFIRLVLTPYYLLQRFDHDERILYELLLQINWSLFHVVTLLLTIEPCHWTQEQRERTQILLSQLIVHLAPKCERLSKELDQFAKQILLSNAKYMPLGVYALARPLMATILGGVTTYLVIIIQFQKISDQL*

>CfagGR29

MTDQVRKIFTKRATVWPHDYRTVRAYKYFRSSYLISAILGYNFFPFTIFKSRFVIIIFTIYALSVSIGFSYLVLSYVKYLDRSPYVYIAAEYITTVILLLFMNADNRAQFLRSLEFIDDKLHVDGSYYYRIQKITQVFVIIVILMRFFYSMAVCFSFRLNCDESPIIVFTSGFILIAVDANQIPRLITFFMIKFRVSLLRNKVENLFQNIGISVIDTKDKRKSLLLCQDIYEDIIDSVNIISNQVDPFFLLSLVCSFPKIMLLLYNIIVLQKRGVPIIHLVALGLEAMQWALIPCLPGILFEMTRAHVDKMKLFLLKHYKDSTRNNYPIKAEIKEFLDYIEIRPCRYLLYRFIPVDLSLPVCLFHLCTTYLIVMIQFSHLFDDAA*

>CfagGR63

KMRVSGVFRVISADRRSAEIQNTFKPINRLTSILSLNCSVSGPNTSWQLFWIVCKALASASVLGFLTFYCLYIKIRYHYNDVNLSIKLTDAVQMCYDYSQYLVDLFFVFKYGRETYAEYHKHIINIDQILITTNYSAIKRRLIKFIAYFIVIWIITSVCDFTAWAITYGTLLPTFYAISYIYFLIKMFSTLDLMSHVMHVEYRMKCIVYQLQECYCDTKDIPGDFSDPIGNKLWFYFESPSKLGNTNETPQPVRTLAGNNPQNVKWLSRCYLLLCEQCIFINSIFGTRILLNSLSLLIDMIRFTNTSVRLVTGSQPTIHASGIYPAAANIL

>CfagGR68.1

MSYFKKYTSTKTEKINIWGAFKPLYFILTLFGLLPYSLKFPKGSGGAIIIHKSIYFNSLCSVSMILILYSFFVLHTQQVFASTENNTMTEVLTTKFNYMLEMLAFLLFVTISYFCAYKNRFNYVKILNAIVSSSNKFVDYKVIRRLQVQVKIVILCLFLLLLIQITTNFTRHDTVRKMMLVTSSFILPQMIQFTLISFYYVLVLMVAGVLKNINEQMKSLCNYNRVSAEFIKLDKRITLNQIEVVYVNMLEMKREINRAFQASILATAIQCFHSIVSESHILYHGLVVEHTLTTHDVCNCSIWIVCQLIKIYTISCSGSMLKEQAAKVGRSLHNIHPGKDDVRLYLEVQHFSSMILYQNAEMTVYDFFPLDSTFTYNVVSAAVMYIVMLVQFDTNKKS*

>CfagGR68.2

MFTSLKQYFSPFVNKDEELCLLQIFKPLYILLSALGLFPQAVRFPDGIKNTTLNIKNSIINSICTLFMIVIVHAFLVFHLQELNVSSKDNSMTEGKMTLMNYIADLVLEILFCTVSYFYVIRDRNVYIIILNDIAICWDRLAMGKRRLILGRLRVHINCVVLTTVLAMILVLAVATYTGDSGVWKMILVTLTFVLPDMIQFTMIAFYFVMILMVVAMLKNIEEEFKVISLVKNSVPNDLVEARLVVTINELRELYVKTLKIKRQINEAFQGPLLVSLVVCFFELVTMPHMIYHGLAFQANFTTHDDIECTVWVLNQLIKMYALAKSGALLKSQ*

>CfagGR68.3

MSSTLTKYFSPVINHEEELCLLQIFKPLYVLLSALGLFPQSIKFPDGIHKTNVELKNSSINSACTMFMIAVIYVFFFFHVQESYMSSKDNTLTEGTMTLINYIVCLIIQVLFCTVSFFRVTCDRKIYITILNEMADCWERMAMGKRRLILGRLRVQVNCVVLPSVLLIFLLLLISRNIGMYVNIWKAILISLTYDLPELIQFGMLAFYFVLVLIIVALLKSIEEELILILHDVEKNNRHKSFEFDMRMGMGEIMKVYVKTLGLKRQVIAAFRTSILVALVSTFHQLVSLLHLMYHGLAFQTNFSTHYIIDCSIWTVNQLLKIYILARSGDLLTSQVNEIGRTIHNIPISGDPDWKVILEVQHFSSLMTYHDAKMTVYGFFPLDATLLFNMVASAAMYLVILVQFDKPE*

>CfagGR68.4

MFSSLRIYFSPFVKKNEELRLLQIFKPLYIVLSFLGLIPCSIEFPQGNVDCIILHKSAFNHSCCAFLTLLLVYVFFGLHVYEVLTSREENALADDQMAKANYIIELVTQFTFCNATYFCAFRYKDIYMSILKEITRSWDDLPYVNRGIILGRIRVKVNCGVIGSICLILLTLTAVTYAGSSSLWKRILITISFNLPEMIQFILIAFYYVLILMVVALMKNIEDHCRMFMKARRSTSIKNSSSKVELGRIPVTLSQMKCVYVKALRVKRQINAVFQAPIMLSLFQCFHTMVSESYDICLGLLYQDNFTTHNLIENSYWVLLQLLKIYALARSGSLLKAEALAIGRTIHNIQSDDDDIKLFVEIQHFSTLMTFQGTEITIFGYFPLEAPLMFNMVAAAAMYLIILVQFAKTR*

>CfagGR68.5

VLCMCHHLESRRNRFNRIILKKDVNRMFSLIITYFTPDQNIVTVFKPVYILLSIVGLFPYSIKFPKGRHAFKITRKTFTLHSLCGLAMFVFLCYNCFYKFKTLISTINKHRSMNLITHINYFVGIFSRLICDVAIYYYTVRNAKVYVNILQEIAFRWSELGLVNVNVNVNPIQRYLHKRLVVAPLLWFLFFLIFRLEIYYAEHNIFGLGIFLFLPEVITSLVVIFYSSLILLVVGLLKNIEMHCKMLVKMKHGVCGTNSNDVDNRNSLHHLERTYVKALEIKREIGKAFETPLAIIICLCFHTALRSTHTMYHGLVISKTINYYKIAERSFWILYPLAKIYVLAYTGNQLQNEVSKIGETLHNIPTGDQERHCLEVQHFISLMSFHKPEITIYDYFAMDITLFFSVMAGLVTYVILLVQIDTPPSSTATGFTFVGFRECCL*

>CfagIR2

MQGETFTVASTPDCITVLRDERNDPLWRETQVGCRVLLLLQEIHKFNITYTEGYHHDENGLYVDDVDIFAKPWSLWSPIMVNCTPIAPVMDWKFGYILRRPIPNIQHFYSLAFSTPTWNVIAAMILLVSVLFYILNRAEQKLSGENLKCYFWRELLTAFGIICQHYISISPMELSSRRIAFISFSLFSYIIYCYYTSTLLSDLVHDRDNEMDLESLGESDYEHAVLDTVAGTIKVVVEQLHNNNKLSSQRLSLMQDKLTNHRLVNISTGLAEVKSSKTALLSDYVSIHSAVIQSFTDSEVCDLIKVDMFSNVLKYLVTSKNFKYIEEFKMSTLRAREAGVLHRLLSPHTAQHFETSECVSAHFQAHFGLVRKPFIILVLGYVLCGFILLGERVHYNRYKVWPYVN

>CfagIR3

MLRKSIFFFLFSSVAGFEDSVIDFSLDFLKARDVKSICLLTCGDRTWNHKFAKNASKISIAVSHVGIDDSMPDLDSIGLCLNPGFSGVGVLIDTKCPLYEEVLMYASENLLFNVNHKWLIIDIDTWMPNISTVSNVEINENFSWLLDTFEKLNLSVDADVTLSLQKGSDNNIYDVYNFGKLRGGNVVVKKLGNWRNKADLINHLNIYKYYRRWDFENSTINYVGVMSTPPIVFDVNMLIGDTPAPGVAVMTVTGTRVLVEIAQLHNIRYNYTIVDRWIGKFERNTTPVAATLVYLKEQDITPILRVPLEIFQRVDMVSPPVTSIETRYYYRIPTTGPGKFENQFLRPLTNGVWACVIAVISLCALVLFLTARSERRPAAVQYAVFSVAATFCQQFFEDGGNDDPTRESSARQLTVLVTGVSCVLIYNYYTSSVVSWLLNGPPPSINSLQELLESPLSLIYQDIGYTRSWLQNPTYYYNKKNAEVEDKLRRFKVFKKKKGEPLLVPLEEGIEMVKAGGYAYHTEVYNANMLISRKFNQEELCELGSLQSMEETHLYIVVPKNSPYKEFYNWNLLRMCETGVVSHLQRKANSPEISCGGSSPRALALGGAAPAFLLLAFGYLLAAVIVLIERLVARNELRLIRKIKDRVPTVGAELLGVD

>CfagIR8a

MPKLDPNTGELMFNEDGEPMYEGYCIDLIQKLSESMDFDYEIITPKTGSFGRRLANGTWDGVVGDLMRAETDIAVSALTMTAEREEVIDFVAPYFEQSGILIVIRKPTRKTSLFKFMTVLRTEVWLSIVAALVLTGFMIWLLDKYSPYSARNNPDAYPYPCREFTLKESFWFALTSFTPQGGGEAPKALSGRTLVAAYWLFVVLMLATFTANLAAFLTVERMQTPVSSLEQLARQSRINYTVVEGSTIHQYFINMKFAEDTLYRVWKEITLNATSDQSQYRVWDYPIREQYGHILLAINASMPVPDAKTGFRQVDEHTDADFAFIHDSAEIKYEVTLNCNLTEVGEVFAEQPYAIAVQQGSRLQEELSRALLDLQKERLLEQLAAKYWNETARQQCPDADESEGITLESLGGVFIATLFGLGLAMITLAWEVFYYKRKEKNKVRQVEEERPKKAFEKDLEKKIAGGVARLRKRDKKEKRGQVTIGDTFKPVSEKDGVSYISVYPKTEYKP

>CfagIR21a

MRFLRTAFFNYILLHYVISQEIEYYPSSFAVKVVSELKSEPNKHKHDLYKREAQWRKFNNNDDPELTKNKTLKRAVDPVFHGHPKTREELWNERFINESLAYDQNPSLISLIHNITLTYLNDCIPIILYDSQVKSRESYLFQNLLKDFPIAYVHGYINENNELAEPKLVRASRECIHFIAFLSDVTRSAKILGKQAESKVVIVARSSQWAVQEFLAGPQSRLFINLIVIGQSFKDGDDDTLEAPYILYSHKLYTDGLGASQPVVLTSWSHGRYSRQVNLFPRKMSKGYAGHRFVVAAANQPPYVFRTIKTDADGGNPRVVWDGIEIRLLTLLSQINNFSIEIKEPQEPHLGPGESVLKEVTGGRADMGVAGIYLTSDRLRDTDMSFSHSSDCAVFVTLMSTALPRYRAILGPFHWTVWLALTLTYLFGIFPLAFSDKHTLKHLLHNSGEVENMFWYVFGTFTNCFTFVGKNSWSKTTKITTRLLIGWYWLFTIIITSCYTGSIIAFVTLPVFPETIDSIQQLLDGFYRVGTLDRGGWEKWFLNSSDPKTNKLLKKLQLVGDVPSGIRNTTKTFFLLPFAFLGSRAELEYIIQSNFTKTIKSKKAQLHISNECFVPFGVSLTFPNNSLYSSKLSGDIARILQSGLIYKMENEVKWEMQRTPSGKFLSAGSGTLKLGAITEKGLTLADTQGMFLLLAAGFLLAAAALISEWMGGCSRKCRPQKKEDAPSSANSREHLIPTPKSDVDSEIKVISDSAESRFRLNPRPDSEDSKDSLEGAIINVTKESIMIHNNFHGSTDCWDSRRSSSVDIDREVQEIFEKDEKRRRIKSGTIPLTDNQREATASKGAFGDHLSDH

>CfagIR25a

MSSLTILLLFLFVPVSFSQTTQNINVLLINEENNALAEKSFEVAKEYVRRNPTLGLAVDPVIVVGNRTDAKAFLENVCRKYNDMLSAKKTPHVVLDFTMTGVGSETIKSFTAALGLPTISGSFGQAGDLRQWRNLDANQTKFLLQVMPPADILPESIRAIVTKQDITNAAIIFDEFFVMDHKYKSLLQNIPTRHVITPVKSFNRDEIKTQLRSLRELDIVNFFVVGSLRTIKNVLDAADENQYFGRKTAWFALTLDKGDISCGCKDATIVYMKPTPDAKSRDRLGKIKTTYSMNGEPEITAAFYFDLSLRTFLTVKSLLDSGKWPNDMRYISCDDYDGKNTPNRTLDLKTAFHEIKETPTYAPFFIPEDDPMNGRSYMEFNTDLSAVTVKDGASIGSRNLGSWKAGLSNPLSLTDPQNMSDYSAQLVYRVVTVEQKPFIIRDDEAPKGFKGYCIDLIEEIRQIVKFDYEITLVPDGNFGTMDENGNWNGIIKELIEKRADIGLSSLSVMAERENVVDFTVPYYDLVGITILMKLPRTPTSLFKFLTVLEDDVWLSILAAYFFTSFLMWVFDKWSPYSYQNNREKYKDDEEKREFNLKECLWFCMTSLTPQGGGEAPKNLSGRLLAATWWLFGFIIIASYTANLAAFLTVSRLDTPIESLDDLSKQYKIQYAPLNGSAAMTYFERMAHIEVRFYEIWKEMSLNDSLSDVERAKLAVWDYPVSDKYSKMWQAMKEAGLPNSVEEAVQRVRDSESSSEGFAWLGDATDVRYYVLTSCDLQMVGDEFSRKPYAIAVQQGSPLKDQFNNAILQLLNKRKLEKLKENWWNNNPEAMKCEKQEDQSDGISIQNIGGVFIVIFMGIGLACITLGVEYWWYKWRKRPVIGDVTQVEPSKTTRNNADNSTTKIGEGFTFRSRNLGLSNFRSKF

>CfagIR41a.1

MITPSILFPIEILLNLLIKEHLQESFCLTFATETKLTVNMPTNISSMIIQPNNSVLVEQILDASEKGCSDYIIQMHEPENFMKAFEKVNHLGDIRRSDKKLIFLPFQDEMYNKSDLTNILTLTETGFVANILLIVPFLQSSDCEVYDMITHTFVGADKEVQKPLYLDRWDSCTSHFERGVNLFPHDMSNLYGKTVKVAAFTYKPYVLLDLDPSLNPLGRDGMEMRIIDEFCRWVNCTVEIVRDDEHEWGEIYENNTGVGVLGNVVEDRADIGITALYSWYEEYRVLDFSAPIIRTAITCIAPAPRILTSWDLPLVPFTWTMWMCILFTFFYASFALSVAQRSTKNVFLDTFGMMITQTREDATSWRIRSITGWMLVTGLVIDNAYSGGLASSFTVPKYEASIDTVEDLVDRKMEWGATHDAWIFSIILSEEPLIKSLLSQFKTYPADILKRKSFSRSMAFSIEHLPAGYFAIGEYITKEAAMDLEIMLDKIYYEQCVVMLRKSSPYTAKLSELVGRLHQSGLMLSWETQVALKYLDFKVQLEVRLSRARKDLEGIEPLSIKQLLGIYIFYFGGVVIALLGFFGELLSKCSKPSIVL

>CfagIR60a

MCISMLKIVFLFSIGVNAKLNPHGPTVVSDFSGCVSEIIDGNFAKSGHLYFVDTFNVSTPVAGIRHAIIKSVHAKLKYSVKIATPTKKDKGICFNNDNTAIEMSVGSHMDHFEATPLAHYFILIIEDYKEFSYIASRLIRSRSWNPSALFILVYFSITGSDDKNIRHAEDMLFCLFKINVINAVVIIPEVNNVRKANIYSWRPYEPPKYCGYYNESIRNRLIVENVCERGKIKYAKKIFESKIPSDMMGCSLKVLALERQPFISHNPLDPNMESLLIDQLAKRYNLSLRYEILNSYRGEKSFHGDWSGALRDLADKKGHLLLGGIFPDDEVHQDFECSTTYLADSYTWVVPRALPKPAWLALFVIFQKTVWLTVITCFVFIALSWMVLAKLSRDPTYRTNLDHYFINTWLSNLGFCAFSRPITHSLRLFFVFINLYCILLITAYQTKLIDVLTNPSFEYQISTVEELVESGLKCGGSEELHDIFENSTDPIDNYFLGEWIDIADIKDALRDVAIHRNFSLLCSRLELAYVSAIIPELSDQFGNYMYYAFPTNVFTVPLETVSTKGFPFMKGFSRILTYFRQHGINNGVIVYFGGYLLRQRALLLKELKHEYNKRPALSIQTLQSGYLALIFGYLCGTIVFIVEI

>CfagIR64a

MNVTAYTSLFTILSIAEIDLIRDVFKYKNLHFGTIFHCSKPENAICLQKSLKKIDLRFSSTKMNSNASHLKQTNDSRVGIVLKTSCQNWTKVFEHFDFNLFKKSLYSWLIFTDDVSSTSEALSRYPIEVDSDVTIISRQEKSYDIFEVYNTGYFTNGRYHVEPVGYWYYKLCMKGHRRTNLDGIVLRSAVVVTHGIGHQTFEEYISRLKPEVDSLHKLKYFTLLNYLREMYNFSLIVQRTNSWGYVTNGSFDGMVGTLQRGETDVGGTPVFIRADRAKFIHYVTATWPSKPCFIFRHPKHPGGFLTIYTRPLSYNVWLCIIALLVFAGSLLCMLIKLRVTRTAGDDDDLSTSLALLSIWSAVCQQGTTVNVRASSVRLVLFSSFLFSLFVYQYYNALVVSTLLRAPPVTIRSLEDLLGSKLKAGVEDVLYNKDYFRRTTDSIALKLYSRKIASSPRPNFLPPDRGMALVK

>CfagIR75p.2

MKILFLFVIVYYLSLGKAFDDNDINMIVSFVTLDDRATAVLTPYVCWSAYELTNLAKSLRDTGISIAASLQPNRPELFLQNLVIVADLRCRGTDDFLIKASDEGFFKSPYRWLLISQDQTELNVLDQLAMLVDSDVIIAQRRGEDYQYVEVYKIIENSQLIYSTRALWRPIDKRSNNTAIITYYNKSKAVANKYGVVEDYRKSKILSTRRMDIRRHTLTMVNVITDSNDTRKHMDDRLNLHQDSITKMSYMVVKICFEMMNSTEKLIFTNTWGYVDKNGSWNGIVERLIKKEGDIGTLTIFTQERMKLIDYIAMVGTTAVRFVFREPPLAYVSNIFALPFTGAVWLAVLVCVLACALFLYITSKWEATMGTHPLQLDGSWADVLILIIGAVLQQGCTLEPRRAAGRIVTLLLFIALTILYAAYSANIVVLLRAPSSSVRSLQDILNSPIKLGASDFSYNRYFFKKLNEPLRKEIYNKKIAPKGKKANFYTMKEGVEKIRKGLFAFHMELNPGYRLIQETYQEDEKCDLVEIDYINEIDPWVPGQKRSPYKDLFKINFIKIRESGIQHCIHQRLHVGKPRCLGTVNTFSSVGIMDMYSAMLATLYGMFMAPAVLLLEIAYKRLMVAREKRMQHNNAHLVY

>CfagIR75q.1

MYRCVTFFCAMFLCSSFALSILKENDRKVIVDIIQSFNKPSDVISNLCWTNTSKKKLTISLTQANKPRSIKFVSALHDNDLVYPEKIIFLIDVSCKETKAFLNEAASRKYFGRPHRWFIINRPANEIRVPLEIDNMHMLPDSEVYVMQLINNSYSINLMYKIKPNREWIIENYGNWSTENGLTISSHAKEALAVRRRDLARASIATSMVITDNSSFADLEVLQYKQIDPVTKSGYHQLTPLYEFMNASREFILTDQWGYRANGTWYGMVGHLADGTAEIAGAVLFITEERMQHVEYMSHPMASSVKFLFREPPLSYQNNLYLLPFQASVWYCIASFVMVLIVAMYVSAYWEAKKVKEKHKGDDTAVLVPTISDVTIFVMCAISQQGSTVELKGMLGRLMILILFLVFLFLYTAYSASIVVLLQSSSNQIRTLTDLLNSKLELGVEDTPYNRYWFMNEKEPIRRAIYEKKIAPSGSKPKFFDLTEGVLQLQKKPFALNCNLGVAYKVMEKYFYEHEKCGLQEISYLQDDNPWQAVRRGSPYREIFKIGLLRNEEFGLNDRTNRIIYSKKPVCSVRGGSFVSVSLVDCYPILLLLLYGMILGVLLLLVEIFHHRKILNMPCGGGKSQRLLVDTTPHSELSDKMQVAEL

>CfagIR75q.2

MKLFCLAVFLFTIQVGCHAEEASKPAMVADVIRAMQRPSAVIAMLCWSSNLKLQLYSALEGENVTQITMMQFLKAGTVAERHAQDQHIVFLADLDCPDIVSYFRTSSLNKHFRSPFRWILIDSGNNDTSERYIPNAVANFDILVDSEVILAHHLGDGSYRLHLIYRIGYNTDWKKEFYGTWDESRRLQKEVMEGEIILRRLDLERIELAICYVLTDNDSINHLYDNVNDHIDTITKVNFPTTNHLLDFLNASRKYVFANTWGYRVNGTWNGMTGYLVREEVEIGGSPMFFTSERISIVDYISSPTPTRSKFVFRQPKLSYENNLFLLSFRASVWYSSIALLLLLVIVLFIVTIWEWKKTRGHEDKKVEADSGILRASVVDVILLIYGAACQQGSTVELKGSLGRIVMLILFLALMFLYTSYAANIVALLQSSSSQIKTLEDLLHSRIKFGVHDTVFNKYYFSTATEPVRKAIYETKVAPRGSTPRFMPMEEGVKKMQKGLFAFHMETGVGYKFVGKYFQESEKCGLKEIQYLQVIDPWLAVRKNTPYKEMFKLGTKRIQEHGLQSRENRLLYEKRPKCSGQGGSFVSVSMVDCYPALLVLFYGSVFSVGLLFIEILTKRRNDILRKISHAKTLSVDVGDY

>CfagIR76b

MTGLELIISSVCNATFCEPVYDNPITEAILSDQKKEIQKIAEDLNGKHLKIGTYDNYPLSWVHTDANGKLTGRGVAFIVLDILRERFNFTFDVVTPVKNYEIGVEGRMQDSLIGLVNSSQVDMAAAFLPILYTYQIFVDFSTILDNGVWMMMLQRPKESAAGSGLLAPFEIHVWYLILAAVLSYGPCITLLTYLRSKVVKDGEKNISLSPSFWFVYGALLKQGTTLAPEANTTRILFTTWWLFIILLSAFYTANLTAFLTLSKFTLDVEYPEDLYKKNYRWVAPEGSTVQYVVNDADENLHFLSKMVANGRGEFRSVNADRQFLPYVTAGAVLVKEQTAIDHLMFEDYLKKTKDKVPEVKRCTYVVAPNPFMEKLRGFAFPKKSKLKLLFDPVFTYLRQSGIVTYLEHRDLPSTKICPLDLQSKDRKLRNSDLSMTYMLMGVGLATAIAVFGGEMIIRYYVRIKIRTNRGDRHKIKTVKSSKHRRFRIQDDSHPPPYESLFGHNSRYKTNGHSTTKIVNGREYWVVGTVSGDTRLIPVRTPSAFLYQRDK

>CfagIR87a

MFPLNMKKCPLVTQAIISEPYVMPPVRQLTNTPYPDAYEFQKGGEINLVKLISEFTNMSLIVRISDVPENWGLIYPNGTATGAYGILRNDSVDLVIGDIEVTRTIRKWFYPTVSYTQDEMTWCVPKSAQASTWDNLVIIFQWTTWVATLLSIVTMGMIFHYIYYRENDRKVTKLPTNSLLFTFSMILGWGASFKPKSATFRILIFTWLFFGMIMSISYESFLRTFLMHPRYEKQISSETDLIQSGIPLGGRAIYRSYFETNNASSFYLYRKYISTSFSEGIKRAALERNFAVVTSRRQAEYQDQKLGKGEQLLYCFKEGNNLYKYGVVLLARRWFPMLERFNNIIRSVSENGLIEKWNQELFIHTVGVDGTSKVVPLGIRHLLGAFIFIGIMYAASIIVFVVELLLNVSKKRKSNKPIAMPVYRVKAVQGDNKSKLQLSDYEGGSNLFS

>Cfag93a

MRIWVVLICIVGVRGEDFPSLITANASIAVVLDRQYLGEKYQPLLDALKDYIKELARVELKHGGVVVHYYSWSTISLKKGFIAVFSIASCEDTWSLFSRTEEEELLLFALTEVDCPRLPPDSAITVTYTDPGQELPQLLLDLRTTRAFNWKSAVILHDDTLNRDMVSRVVQSLTSQIDDEDVPTISVTVFKMKHEINEYLRRKEMHRVLSKLPVKHIGENFIAIVTSDVMSTMAETARDLYMSNTQAQWLYVISDTSIRNSNLSSLVNELYEGENIAYIYNITDDREDCKNGLMCYCEDMMNAFISALDSAVQEEFDVAAQVSDEEWEAIRPTKIQRRDTLLKHMQQHIAVNSACGNCSTWQAMAANTWGSTYGGYVQDNAAAPDNDTNEAIQKIELLQVAYWRPSDGLRFTDFLFPHIVHGFRGKVLPIITYNNPPWTILKANESGSISSYSGLIFDIVDQLAKNKNFTLKLIFPGDLKDVLSNKTVTNDMYSQSAKLTMMAVARKQAAFAAAAFTVLSDRNPGINYTIPVSTQSYSFLIARPRELSRAMLFLLPFTTDTWLCLGFAVVLMGPTLYVVHRLSPYYEAVGVTRQGGLATIHNCLWYIYGALLQQGGMYLPRADSGRLVVGTWWLVVLVVVTTYSGNLVAFLTFPKQEVPVTTVSELLENRALYTWSISRGSYLEFELKNSDEPKYVSLLKGAELTSDSSGLEGNLASGSPLLSRVRKDRHVIIDWKLRLSYLMRAEHLATDKCDFALSAEEFLDEQVAMIVPAGSPYLPVFNKEINRMQKAGLITKWLSAYLPKRDRCWKTSSIMQEVDNHTVNLSDMQGSFFVLFLGFFSASSVLLMEWFYHRRKSQKEDDAIKPYVE

>Cfag-iGluR5

MTINYAREAVIDFTKPFMNLGIGILFKVPTSQPTRLFSFLNPLAIEIWLYVLAAYILVSFTLFVMARFSPYEWSSSTHVCGHETKLLTNQFSVCNSLWFITGTFLRQGSGLNPKATSTRIVGGIWWFFTLIILSSYTANLAAFLTVERTVLPIQSAADLAAQNSVQYGTLNGGSTMTFFRDSNIDIYQKMWQHMSTTSPPALVSSYEEGVRRVLQGNYAFLMESTMLDHRVQRDCNLTQIGGLLDSKGYGIATWKGSPWRDRISLAILELQEKGVIQILYDKWWKNTGDVCNRDGKDSKANPLGVQNIGGVFVTLLCGLALAIVVAILEFCWHTKKNASQGRQSLCSEMGQELRTAMR

>Cfag-iGluR3

MPYEHGDKISRMIKKTEIDGLTGVVRFNEEGHRKNFSLQVMEMTVEGEMIKIATWYDNKGFVPVVPKLPGPSIPGVYNRNKTYIVSTIEEPPYIMRRNSDYAEFTPNDPYKGFCVDLAKMLSDKLEIKYEIRVVKDGKYGSENPKIIGGWDGMIGEILRKEVDMAIAPLTVTVERETVVDFSKPFLSFDIKPSLKNIADEPGAIFSFLDPLSTEVWLCLMFSVLAVTVVLFIVSRFSPYEWRVVSYTDTQSSEHTEVATTKTTVVNEFSFWNSMWFSVGSFMQQGSDITPRSVSGRIVGTVWWFFTFIVISSYTASLASYLTLQRIAEPSQTYSKVAACPEDTSEGIRSIAIPRSRHSWLSYLLDHSSAGDDAEKPCEMLVTVTNSGVKDFAVALQKGSELRDGINLALQSLKDDGELQKLIRKWFTKAECDVTEQNMHVTELTLGQVAGLFYVLV

>Cfag-iGluR4

MQGLTPSPMSILDSLCKEFLAVNVSAILYLMNHEQYGRSTASAQYFLQLAGYLGIPVIAWNADNSGLEKRASHAALRLQLAPSIEHQTAAMLSILERYKWHQFSVVTSAIAGHDDFIQAVRERVTALQDRFKFTILNAVVVKKPTDLNELVTSEARVMLLYATREEAADILSTAGDLHLTSENFVWIVTQSVLGSMQQPNKFPVGMLGIHFDTSSSSLIAEIATAVKVFAYGVESYILAPENARHPLGTRLSCSGAGASEARWSTGERFYQHLKNVSVESEASRPSIEFTPDGELRAAELKIMNLRPAIGEQLVWEEIGTWNSYPKERLDIKDIVWPGGLHTPPQGVPEKFHMRITFLEEPPYINLAPPDPISGRCILDRGVICRIAPEAEVAGLEAGTAHRNSSLYQCCSGFCIDLLQQLAEQLGFTYELSRVEDGRWG

>Cfag-iGluR8

MMSDYHSYLITSLDLHSVDLEEFKYGGTNITSLRLLDPERADVQRVVRDWVYDEARKGRKLQLGHTSAKENMTFIKTETALMYDAVHLFAKALHDLDTSQQIDVRPLSCEAEDTWPHGYSLINYMKIVEMKGLTGVIKFDHQGFRSDFTLDIIELTRDGLQKAGIWNSSEGVNYTRSYGENQKQIVEILQNKTLIVTTILSAPYCMRKEASEKLTGNAQFEGYAIDLIHEISKILGFNYTFKLAPDGRYGSFNRETKEWDGMIRELLEQRADVAIADLTITYDREQVVDFTMPFMNLGISVLYRKPIKQPPNLFSFLSPLSLDVWIYMATAYLGVSVLLFILARYVLSFLLKQTHLIFCGIPLSYKVTLFLFVC

>Cfag-iGluR9

MATHCCYGLAMDLLENIAQELEFDFHLYLVEDGLYGSRKLVRSFSKLHEFTNFLNDEPMFTMSEHLNYRAQFRNGFKANSKEQYSEPNYDDNIDEESQKWNGIVGDLVSGSAHMSFAALSVSAARAEVIDFSQPYFYSGISLLAAPNQKADIPLLAFLLPFSTELWIAIFLSLNVTAIAVAIYEWLSPFGLNPWGRQRSKNFSLSSALWVMWGLLCGHLVAFKAPKSWPNKFLINVWGGFSVIFVASYTANIAALIAGLFFHNAVDDYQGRNNWLSLRVGTAKSSISEYYVQRGNPQLAQRMRGYALQNIEEGIQRLRNRTLDLLIADTPVLDYYRATDHGCKLQRVGDHAFIEDTYAIGMTKGFPLQKSISAAIAKYSTNGYMDILTDKWYGGLPCFKLSQDYGIQPKPLGVAAVAGVFLLLLV

3. Cydia nigricana

>CnigOR1

MALSRIFEIMKTKFADGEVDSPLQYKYVTRLQLMMSSIGSWPHTQFGRHKLHTVLSLYNLVLNGLCLLMFILGVLYWRQRRENMSFFDSGHIFLCMIFDLLVLVRLLVARTTKYQETIKDYLLEFHLFYFKNRSPYAAKIHTQIHTISGMFTFYVICQMASGVALFVLMPWYNNLRNGMFGKHRPENSTFENSAYYYLPDACYTTLKGYWMLFAFNSFTSYIVTIGLFVFDLLISLMVFQTWGHLKILQHNLLNMPPPENSTTGLYSTEENVKIRNLLKEIHEHHNLIIKFVDRCSDAFSEYLFSFYLFMQFITCILLLEVSSFTADALAKYGPLTIGMHQQLIQVSILFEMLNTKSHQLIDAIYAIPWEYMDTSNRKTVIFLIRAIQKPVSLKAGGMVPVGVNTMMAVLKGSVSYYMMLKALAGEQ*

>CnigOR2

MTVTEVFNSLKRRFDDSDANSPLDFKYVSILRFMLSSIGCWPHKQFGRRWLDFILSMYNVLLIVVAIALPTLGAIYIWYKRDTISFFDMGHVLLCMFLELLFLQRLIMARTVKYREILKDYLLEFHLFHFKNRTQYASKVHTQIHNMSVILTLYMACQMFCGMSLFTFMPWYNNYKNGMFGPDRPANRTFEQAVYFHCFTDDVYTTLKGYWILFAFNIPTAWNTSNGLVAFDLLICLIIFQIWGHLKILKHSLLSIVPKDGVYTAEENMRVREILKEIVDHHKFVIKFVDKCSEAFSEELFVFYLMMQLLTCTCLIEVSSLTADALAKYGPLTVVNHQQLIQVSIMFEIIGTKSEQLMDAVYALPWQCMDTRNRRTVMILLQRAQTPITLKAAKMVPVGLSTMTAVLKTSFSYYMVLNAVAGER*

>CnigOR3

MEETTQTFHRVLSVAGISIFAKKNWDSKLWLSLQIFNVVMGFLTIVFTTAFVVVNMSDILVCIQGACIWTTGLIMFISFCVCLAFRRQFREFLNEMGFKDAMLEMPLIEYVLKLERAGRLNELKGMVVDSQEKLLKLTRVLLKTYVFSVWACATLYLSDSVYSMFTREDDNLRLLGFEMWVPWSLENFTVYIITYFFSAYSGYLCCIAYPGFQLTIILLVGQTARQLRILTFILMHLDELVLEMTGQRDEKWQAYSTAILSQCVDHYIKLKRFSNKLNVICRPFYLALILDAIMLVCVCSVKIAISNKTSPDTMKYYVHEFCFIMVVLMFCTLGQHVENECARLETAVTENWYIYDRTHKTHVKIFKMALSQRMPIYIFGTITLSAPTFTWFLKTGMSFFTLVMSVLDEN*

>CnigOR5

MALRVFRSVKIWFDDSDAKHPLEFNYVRQLIFLLNAIGSWPHNQFGRDRLHIVYSIYNVFLIGVAITISSAAAGYIWFNRETISFSFMGHVILCILLETLYLQRLLTSRTQKYGETVKDLLLQFHLFYFQNRSQYASKLFKKVQIISKIFTIYVTCHILTGVGLFTFMPWYNNYRSGMFGPDPPVNKTFEHSLYFYCFTDKVYTTLKGYWILFFFNIPTSFHTSSGLLTFDLLLSITVFQILGHLKIMKHDLLSIPSTRDLYSREENMRVRKTLKGIIDHHNLIIKFVDKCSDAFSNYLFMFYMLMQLLTIVVTLEVTAYTADALAKYGPLTVAIYQPLIQISILFEMISTQSEKLVEAIYQIPWECMDTSNRRTVMIFLHRAQTPVALKAAKMVPVGVMTMTAILKTTFSYYMLLNAVAESADQKMGS*

>CnigOR6

MPSTLQRSEPKVFTLDYMRSLRFTLETIGQWPNRTLGDFSRRAILLSIYHKFLVCWFCFTETIAFFYMKKHRETIRFIDMGQIYTNLFLTGLFLQRASLPFQKNYKECVKKFVLEFHLMHHEHISEFATKESRKVNKICKIATKVIYLQLACGMFAYNLSPLVRNYYEGMFASELPANKSFVHSVDYLLPFDAYRSLTGYIIVFTYNWFPTYNIPTAMGIYDLLVFVMVFHMVGHMNILYNSLKEFPRPKEGQSEDPLPSTREYNAEIFQCLKNVIRHYQIIKEFMGDMTAAFDLTLCCYLGFHQIMCCLMLLECSTLEPEALVKYGMLAAVIFQQLIQTSVAFELIKSKSESLGDEVYAVPWEYMDVKNRRILLLFLRNVQDPLGLKACGMVPVGVLTMSTIIRTSFSYYLMLATFAD*

>CnigOR7

MAKHSRERVLDLDYMRVIRLCLDTAAHWPNEQFGPKTMRTRILSMYHKSMLTLLIYAEISALFLVRVRMKTHDYIDLGQDYLSILMSVVMITRMTLILQEKYGLLIKNFVSKFHLLKHKHDSEFAAKENHRINRICRIATLVIMIECFMGQVMFNVVPIYVNIKAGLFTDRAHRPPNVTFVHSVNYYFIIDQYNDAIGYTIVSFLNAYISYICGVSFCGLDLLIYIIVFHILGHINILVDKMRNFPRPKSLPDESEESSNKKYNEEAFETLKNLIQHDQLIKEFLKDTSETFGITLCICLLFHQVSGCILLLEISPMTAEALTRYGPLILVLYNQLIQMSVIFELISAKCNTLSDEAYELPWELMDTKNRKTMLLFLLNVQRPRGLKACGLVSVGVLTMSQILKTSISYFLILRTLGNV*

>CnigOR8

MDTGAKDIFDVPYMRMIRFSLNSIAQWPYNTLGPRTMRTRILSIYHYVMVTVSTFLEISCVFYVRKNTDKEFIVLGHDYFNILMGVVIIQRMTLSFQKRYCLLVKNFVTKFHLMNHQYKSDFAAMELRRITKICNIAAVIIHIQIFFSMMFFNMVPLWKNIHAGMFSSHRPENGTFVHSGNYLSFVDQYTDIRGYFLVFFLNFYPSYNAAVTFLCMDLLIFIMVFHIAGHLNILLHDLRYFPRPDGMEQCMETGTRKYNEEVFVRLKDLIDRDQAIKEFMINISETFGISLCIYLAFHQVTGCVLLLECSPMTPEALGNYGFLTLMMFQQLIQTSVIFEFISTKSDTLADEVYSLPWELMDVRNRKAVLLFLKNVQPPRALKAGGVVSVGVLTMSTIIKTSCSYFLMLKTLTVEE*

>CnigOR9

MPSYPRDVFSLTYMSRIRFLLNMIASWPNQEFGASKIWWQAASSYRCLLITFVIFNMTTTISYLQKYVNHDLAHSYVNMMLASVYLQRLFLPFQKKYCLMIKRFVLEFHLIHYKHKTENAAQVYDRVNRICAIMTAISMIHTAGLPLFYNGIPLYNNIKAGMFTKHRPPNGTFQHAVYFDLPFDQYGTFDGYLIVFFYNIYVSYNACIGICMYDALVFCIVFHIWGHLNILIHDLKEFPLTSLQAFKMRVPSQFAQEDMFGRLKDIVRHHQMIKEFMRCTSEAFSISLCYYLLFHQLSGCVLLLKCSSLDPIALGRYGLLTIMVFQQLIETSVIFELVNSKSDILADHVYGLPWEDMDLRNRRVALILLHNVQKSLAVKAGDMVPVGVLTMSTIIKTSCSYFIMLRTFTDE*

>CnigOR12

MAPELSDISKPSSSLKQPDRIKKQSDSSDGFKQSDSFKQNRFCWSVFGLWPGKIPEKYYKVYSFTYLIISYVIYNALLTLNLYHTPRRIDALIREIIFTFNEVAVGAKLSMILFKRRKLAAIFEMLDCEEFKGNDEIGREIVMRYNGYYKKYLFFNTALSHCTFFFSVFFPVFGFWIFGNTLDLPICKYYFISDETRDAYFTSIFIYQSVFMYSHMMYNINIDTLIAGFMVLAIGQVKVLCHDLENLKMEKSDGDQSIMDLKQRYKLRKVLNHYELFLEYCDKFQDLIGGTMFVQYGIGSGIICVIMCGLLLPSPLAAKVFMVGYFMAMNLQIFVPAWLGTQLTYESAELTTAAYKSEWLPRSERYKKSIKLLMERAKNPVIITGLKIFPLSLPSYIQIMKTAYSCFALLRIIQDRQETAAP*

>CnigOR13

MRPLRQIDCFKVNMKFWKFLAIWPPNDKLYYRYYQIFFAAFILFNNLLTTINFIFLPRQLDMFVDEMIFYFMEVAVTSKFLTFIIMRDKILQILSMLESDIFQPDTEYGLKIIDKAKKFNVRYFKIVAVVSAIAHLSHIIPPVLLHFILHVKLGLPICRFSFLSDDTVQKFIYPLYELQALYMHSQVLFNISIDTFVLGILILAIAQLDILDENLRRVTDKNQIVKQAFECVQRAEKDKAMKQLNDCIIHYGELGQFCDLVQDVFSITLFVQFSVASCIICVVLFRFTLPAPWQYFIFLGSYMFIMILQILVPCWFGTRIQDKSQQLSQAVYDCDWTAKSRYFKSSLRLFVERANKPLSVTAGKMFPLSLTTFTSIMNSSYSFFTLLRHMQSRQN*

>CnigOR14

MDAMKSLWASDSTTVFVPVFDDVEFKPFRETYKIITFNMVVGMLYPTPETAVCRLVGIALALLSITPMVLIAFIDVWNSWQRGDFINIVRHTTVLGPILAAIFKMMLFFYTRDKAWSIIKKIDADHARYNILPESNKETVRRHIQNTQYYSEKCWFMTVAVTVLTFPLTAVVLNFYNFVFIDDPSKYMIHDLEKPFSPPEDRFSSPYFEIMFGYMSYCSLWYIISFTGFDAFFGITINHACMKSELACKIMEDAMLEEDRDSRQRRMKEVISEQNDFFSMVELVQETFNFWLGLIVMATMCQICNCMYQIIEGYGIDPRYIVFILGTVAHIYVPCQYAAKLQVSALDV

>CnigOR15

MKSFRRKESSTLPLSPATEDLEFKPFRETYKIITFTMIVGMLYPTPNTEVCRVVGILTILVTMSPVCIVAMLDMWNSWFRGDIINIIRHTTVIGPFLGAIFKMMLFFYSRKEAWSIIKKMDADHARCNTLPEPHKEIARRHIQNTQYYSEKCWSITVATCVLTFPLTAVILTFYNYACKDEPVKYMIHDIDKPFSPKEDRFSSPYFEIMFFYMAYCSVFYIISFTGFDAFFGITINHACMKMELACRTMEDAMLERDRGSRRRRMLDVIVEQNDLFRMVELIQETFAIWLGIIVIATMLQICNCMYQIIEGYGIDPRYLVFIVGAIAHIYLPCRYAAKLQVSALDVATHLYCCGWERVYDEQARKMILFMIARAQIPMKITAFNMFDFDMELFVSILQTSYSVFTLLRS*

>CnigOR16

MVKSKIDIGDLYLSRAKLVMSFLGVWMPPPNESIIRKCFKFFMLSLQFIFLVFQLIYMCQVLGDIEEVSQTSFLLFTQASLCFKIFVFHFDMESFRELVVQMNSDVFMPQTEGHEKILKLQASRIKRLLLGFIIGCLGTCSLFVLRPLFDDANRSFPFKMWMPVSPEHSPQYELGFLFQLTTIFMSAFMYFGVDSVCLSMVIFACAEIDIIKEKIMNVKPIAERRINRSITVKNILNEHYKILVECVAHHQAIMKFIQQVEDTNHAHLLFQLSGTVGLICMSALRILVVDPRSVQFMSVVLYLVSLSSQQFVCCWSGHELTATSLELHTAVYECCWYEQDVPFNRALLIMMLRLGRPMEFRAGRYISVTLSRQLFVAILRTSYSYFAVLQQTNSRNEAVEIEN*

>CnigOR71

MVVRSTEYVEIFSSMPCLCNLLLAIFKSYKMVVYRPVFNNLVWELRTMWPQGTVTEEEDRIISRTLRSLNMVVKGYYWCNVFLVIIFLSPSFVALGYRAAGHDTPLILPYWYWYPFDPYEGGPGYAFALAFEDFHGCSAIGFMVMGDLLFCIFLSHIAIQFDLLAVRTQKLVPTIEPKNRLSAFTTEQMRNENCNTPEWEKTHLKELAAIIERHRALIRLSGDVEEMFSGALLLNFLNSSMIFCFCGFCSVIVEKWNEFSYKSFLVTALAQTYLLCAHGQKLIDSSKGLTNALYSCLWYNASKKVKSSVLIAMHRSQKEVHVTTYGFSVINMASYATILKTAWSYLSLLLNVYK

>CnigOR18

MAVSAVDNISLFLNRPRNILLYLGIWLRPANYVSLYVAYAIVVMFTQYSFVFFEFIYIALAWGDMDAVTEASFLLFTQASVCYKVTRFMINKNNLVFLLGFMEQEVFQAQNERHVKCLLHQSMMIRRLCLFFLGSALTTCTLWGLMPIVDSTGGERIFPFLIWMPVGPEKSPQYELGYFYQMVAIYISAFLFIAVDSVALSMIMFGCAQLEIIMDQVQQIKRVPMSGKLKKQDREQLIQENQVLFVECLKHHQAVIRFIETVEDTYHANIFFQLSGSVAIICIIGLRITATTPGSVQFISMLNYMVTMLSQLFLYCWCGNELTIRSEILREVMYLCPWHEQSNSFRRLLWVAMERMKRPIIFKAGHYIPLSRPTFVAILRSSYSYFAVLNQTRSKEK*

>CnigOR19

MKNYFILKNLCRKMYLSGAGDFWFEEGEISKGKSLQYQMLCCILFSIYISMTILEIIGIFFGDMPSDERSDCTTFAVSHTIVLGKIFSVIVNRKRVKELNRKMVEICEDHEDEHRVAENYRIIKINVVAYAVSVYGSFVFFLFEGIRKMMTGSHFITIVTYWPFYEDNSVTAVSFRFFTTLVLAVMMVTMICIDSFAIVTLIMYKYKFITLRYYLEGLREQFDRKNYAGNEESATKHLHAGLVEGIMMHSNLIRLSKDIDRSVGAVLALQVCLSSGSAV

>CnigOR20

MNQLKVDCMKYKSFNETFKLCSFALALGLIYPNRKTVCIRSTLFFFVLLFNFGTLFWFIWYTVKCLWQLDIYNSTRNITVGVIIILFVFKTIYANFKNDMFASLLEQINKDLLKGNNMEEDYQEIYDYYIKQGLFGQKCYLWIPVILTLIFPTYAGISMTYESLRTDNFKKVMLHEMDLKYIEDKQYDFPYFELVFAYYFFGICALVPNFAGFDGSFCIATSHLRMKIKLMTHSIQRAFTDSKDIIELKARLRTCVKDHQEALEFYTLIQKLYGGWLFAVFLLTSFLISCNLYQIYLTGIDPRYTMFAATGVFHMYTPCYFASCLIELGEQSSTDIYCAKWENWTDPTVTKFLIFIMARAQKKLLLNGLGIVTFNMETFVSLMQTSYSFFTLITSK*

>CnigOR25

MFGSLLELSDDFFAYNLKYLFLVGLWPDDPWARAHPTLYKMYENNTHLLSIIFLIISGIGTYQIKDDVVLLMTNLDKCLVAYNFVAKVGIFVFKRRQVEIIILEIINSGDQFTEERKKMTLMLITVITGLSTSIVGAFSALALYHNEMSVEAWMPFDPMESTMNLLTAAQLLAITFVVPVIWRAIAIQGIVCSLIMYVCDQLVELQDRIRSLEFTSTTEKAVRDEFKDIVKKHVRLMGYTQDMNKIFEEYFLIQNLAVTLELCLNALMATMVGFDQKTLLATFFAFLCVALMNAYIYCYLGNEMITQSENLALAAYESSWISWPLDLQKDLVILLRVAQKPLYMSAGGMVAMSIQTYSQTLYNGYSIFAVLNDVVA*

>CnigOR26

MANEGELLSYLSLTPHLKVLRNYGIFPLDSTSPNLNKRLHNFYICISFSLMILYTLLQIIHVFQVRTDIEKVMDAMFLLLTFLDCIFKQVMFLKKPHKILKLLNIMKGPSFNQGLDEHRPLLVRTINHAQVLLRLFNKLCIVTCFLWITLPVYLRLKNESVEFTIWVPFDTNENTKFYIVICYVWMQTTWLGLNNSTMDIFIVYLLAQIKTQISILRLNLENLVSRCKEESRTASYSFTELLELRFRGIIYHYNQIIKFSKINEEVFSSAILFQFLVSGWIICTTAYRTMNMNPLSGEFLSMILYMVCILSEVFLFCFYGNEVAHESQRLMQSAYCMHWEEMPVKYRRYLIIFMERIKCSILPKAGKIVPLSINTFVQIVKTSYTFYTFLSNSNAN*

>CnigOR27

MLKKHVAKLEDPDHPLLGPTLWGLQSWGMWQPNRRLSPIIYNLIHLAATLFVVSQYIELWFIRADLELALRNLSVTMLSSVCVVKAGTFVVWQPYWQDIIQFVSKLEKSQLEKKDKTTSTIIEGYTKYSRKVTYFYWCLVTATVFTVILAPLGVFLSSSEQRALMLNGTIPFPEIMSSWVPFDKRKGFGYWFQIVEHSVICFYGGGIVATYDANTVALMSFFCGQLDLLIENSKRLFSEDGKLVSYSEAMERIKQCHMHHLSLIKYSKILNSLLSPVMFLYVVICSLMICASAILLTKDGTTAMQRIWVAEYLAALIAQLFLYCWHSNEVYFMSEKVDRGVYESEWWRCGVGLRRYVVLLGGQLRKTIVFEAGPFTNLTVATFVAILKGSYSYYTLLSNNEG*

>CnigOR28

MDTLARVMFLLLCHVTSIAKQIVFMARASRIAKLVQDFDDMAYNPEETSRKNLLIERTRGASRLGAAYAGTAALTCTLWTIFPFLGRLGGAHIIFALWIPFSYYSWPKFLIVLFYTYYVTSLVGIANTTMDAFIAIILGQCKTQLAILKMDFESLAERANERALETGEQKDKAAAALLVRCIKHHHKICDTSREVQAIFGGAVLLQFGIGGWILCMAAYKIVGLNVASLEFVSMVMFLMCILTELFLYCYYGNEVAVESAQLSDAVYGMDWVGPDGVGKEVRRALPFVICCSA

>CnigOR29

MIKEFLNNLEDPNRPLFGPNYWLLNEIGLLLPKNWMERIWKIIIHEIVTLFVVTQYMELYVIRSDLDLVLTNLKISMLSIVCVIKANTFVFWQDNWRQVIDYITEADKFERDNQDEAKGNIINAYTRYCRRVTYFYWVLVFTTFITTITAPLMKYCSSEQFREDFHNGTEPFPHIFSAWVPIDKENFPGCWITVMWHIGLCAYGAIIMAAYDTSVMVILVYFGGKLDLLRIRCREMFDTEGKGVSDDISDKVVRQLHQIHVQLLKHSRLFNSVLSPVMFCYVVMCSLMICASAFQLTAATNTTQKLLMAEYLIFGIAQLFMFCWHSNDVIYKSQDVMMGPYESDWWAANLQQRNNVLILQGQMRIVHIYTAGPFTDLTLSTFVAILKGAYSYYTILRK*

>CnigOR31

MSNILSENMKFDNIFWIATTAMRLNRSHPYIPRDKKWRTQFTAIMILSFFCSLFLMYTLFFHNIPCAAYADACKSAIMSIVAFTITYKYRIMLQYQESITDLIRIVDNDYELAKEFCEDEQRIVYKYSQRGVKVTQYWFVSACSTSAIFPVKAFVLMAKSYLAGEFHLVPMFEMSYPWILDDYKNVHVVFVVFFGLTLFFDFYATSMYVGFDPIVPIFMLHLCGQLDILNLRISKLFSNTEDSAETMRGNLRRIILQLQDIYKFIEIIKTNFTVLYEFIMKTTTFLLPLTAFQITESLRNGEINLEFIGFFTGVILHFYIPCYYSDLLMERGENFRLAIYSCGWEKHWDQRVMRTILFMLTRALKPIVISTVFCAICLDTFAQMCREAYSIFNLMNAAW

>CnigOR32

MMNRIFLNLLSRYIPVWTQETPALANTVQRLIWNTGIWHYQSLGLHWFAKFAIFCFISTNLTQVAALVIERDDSTRVFETFSVLSFCSMGTLKLFNLYTNRKRWTFLLSQVKLLEREELHGKLLSYTDSDIEEDYTPQVIAKYTKRHAFTSSTLLKLYSITAIIFIATPFLEYAVTKDASYYPHILPGWAPLDNVGVAGYLLTLIFEVVASVYCLFLHVAFDCASVGIMIFICGQFSLLRWKTEKIAGSGKDCMPSMKRNVEGHLRIIECHGTHTALCTVIKELDTVLRGILGVYFLVATMTVCSVAVRLSTETLSFMQLVSLLQYMCGTLTQLFLFCRYGDAVIHESSFGMGEGPFGAAWWSLCPRTRRQVA

>CnigOR35

MSIEGYKRVRNKITSRFTFQNILRCLEDPKHPSTGPCLRFINLTGNWHPNMKLKSTRFKQLVFYITMTLFFSQYLKCVIGLDLSALLFLLQTAPFHMGTPKSIFFRKNHVLWQKMIDYMSRTELEQLSDKDEEVRAVLDEYIRRARRVIYLYWSLVICGNISLFTEPYLKNQIVENGTDIYVPLFHFYLPWNQDVPPGYYYSMFLQTILGNIMSSYVISWDSLVISTFIFFIGQLKISRVYCTKVIDPESAERSHQNIIKCHRFHTTLVEYHKLFQKLISSVMFIYLIVVSANLGSCIIQISNASGDLPVMMGAIFFVFCILIQLLTFYWFSNEVTVESLSVSSGIFESKWMTMDAKIQKEVALLQLTMSKRLCFRAGPSNEMSLNTFVAILKTSYSFFTLLKETK*

>CnigOR37

MECFSRAQNGFQRLKKLLRENSFDNLVSIVMVMPSMVGFEITRKKISVPFWIIHISLLIYVYGMGSLVYQVQHARVASDFIKSFVNVSILVLTANNSYWWLTHRDLLRSVLSKANESDKRTIQAGLFVDKHKHSLHMIKRILFIFYFINLTNEFTSYLPKRADLNEKTFSMTPCVGLKPLTSFPQREICIALTSLQELTIVAVVLNFQTTMLLLIAHTSATYQLLSDEIMTFNTLLTNPSNYDSVKDKLGVIIKRHILILDITNDIRVLYSIPMGINFGSNAVCMCFFFFLEPEEYFNFMPIAMYCFVVFFLYCFLGQRLTNAAEVFSRAVYSCGWEMMGIKEQRSIFIMLLQSQKDVDLLAADLIPVNMRTFASTSQGIYKFATVFKL*

>CnigOR39

MLRVVAVKIEAGANSISIFFYLLATVSIVLYFFNYYASMLVFVIVGCRETGDILAGIMVLSISMNSLIGINKLLYMYRHQDKVQSLVADYIAYDRVPLWPGTAALMAELMRSVKKRLILFFTVTMGNAFIFNLQPLVMPSRLTSNLYDKHVVYGLKFILGITNYPIAVVNIVACTFFICYITSSIGGLLIVTTGYSEVRLLALGQEMRDLWSDAHKHYRDNFEGDSHDKQAASELNNYIKYRLQVIVKSHAINIDLIKKLEDLFRNAILVEFILLTAGLSIDLLGGLEDTYIILPFSIMQVSMDCYLGQKLMDASIVFENSIYDCKWENFDVKNRKTVLLMLKMSQRTLSLSAGGVATLNYECLMAMYKAVYSAYTTLRSTVE*

>CnigOR42

MSTPTFNDVFHQIRINLSIMGIQEDKSRIGVTFYIIYAMVFTMVTSEIVFFSVNMAPENFLELTGLAPCICVGILSLLKIAALAWKKETIFSLADALEKLSTENLKDPVKTNIVSSDINLLKTLIKYYFVLNAVLICVYNFSTPFYILYHYLTTNEEVFILPYAVIVPFSTKTWPTWTFVYVFSVVCGFICVLFFTAVDALYFTLTSYVCTIFAVLSNEIICLDQPSGDVLDQIIRKHQNVLKLAEDLEDIFTLPNFFNVLVGSIEICALGFNLLIGDWNNVPGCMLFIVSVLCQLFMMSVFGEKLIEASIKIGESAFLCDWYKMNQKTQKVLLMLITRTRKPTRLTAFKYSVICYEGFTKIISNSWSYFTILRTVYSPEDQ*

>CnigOR44

MKFPSENMRKKLSFLTPFLPYGVLESWDDLNPRLYHAVHIYWLKFYGLWYNTRSKTSLLFWVHIVYATVVLWLVCFLPGIGEVFYLLKRRDNIGDIAEGLYLFLSEMYTYIKLSVFWLKRKEIMALLEYLHRDEFKVKEPEHREILRKSIKRARFVMTYYSTMCVGAVSVGILMPLAEQFEVLPTNVEYPYFDVYKSPAYGIIYIHHIYYKPATCIIDGVMDTILAAFIASAIGQIDVLAFNLRNLNLIAERRRNMLPFTKVNVKKSIKYMPSVDHEESTRHCVRAVLKDIVKHHNSIINYVSLIESAFSLASALQLMLSVMVLCLIGIQFLSIEEPSSHPIQIAWMAIYLTCMLIEVFIICWFGDELIWKSWELHQAAFDSPWPSTDPKTAMFIIIFMERCKRPLRVTAGKIFTLSLDTYTILINWAYKAFAVMRKMKK*

>CnigOR47

MQSLKSLLGIQKTATKEVYTFKPNTTVSLFYNICALVFLGCGTNFMFDDLKLPRKVVQICRLVSRVFGLFVLMLMFSACGAFFTQHNLDMMRKAQLWLYGPTSLTLYAMYLNSVIRKEKIKRLGYTIAVVLREMSTDTEIEKEMVKKTWRFSYAFAFLVNGLFFCTGIVTGYVATTTNETFTTVVPAWPDLNDHTTAASIARIAHYCVWYIFLVRVGSIYFIILAITIGLQFQYGIMCNYFHSLNNIFDEDGTHEEKEQKYEDAFKFGVKMHSLTLWCIDQTQVTCGVAFSSQFITNLLTLIALMVQFVFMERTFGNCLNVFLFAGVTLTGTGIFMWNAGDVTYEATKLHTAMFHSGWHNCQGQASLRVRKLLTIAMQRAQDRVTIKGFGILELSYDSYISMVKFAYSVFSVLY*

>CnigOR49

MITYILKKLENPKQVLLGPNVKALKFWGLLLPENVILKYVYICLHISIIYFTATEYVDIWFIKSDMNMLLENLKITMLASVSVVKVSTFLIWQNSWRDIIDYVTESDLNQRKTTDETNIAIIKQNTKYSRKITYLYWSLMYTTVVIVMVQPIMKYVFSQTYRDNVKSGEQSYIQVVSSWVPFDKSNILGYLAACAFQSYAAIYGGGWITSFDTTAIVTMVFFKGELQLLRRDSAKIFGVKDNPVSREEAETRLKNCYTRHVNLIKYSNLFDSCLSPIMLFYMFVCSVMLCVTAYQIRTGTSMLQTILQVEYLVFGVSQLFMYCWHSNDVMYTSQEVIYGPYESRWWAENALRKDLVILLGQYRKEIVFSAGPFTNLTLPTFISILKGAYSYYTLLTKSQIDI*

>CnigOR53

MQLTHCFKVSYFWLILGGVWYPRSLDNTRMIYAVNIYRVFATIFINVGMLAIQFIYFFTVVGKDLDKTVDATALFTFVGYLYKALTVIKNRQRINKLLDIVDNETDKDDLLRNMAANINFISYFYNGWACLTAIMWNLIPFTKATLTLPFYYPDLPPSSPWFVRWYIYQATILIINGVAQTSADHLFGGLMAFAATQLKLLQHKLEAIGTKTDSTVENDAQREQEDYEETVSCVEYHLKIIWFVNELTDIFGGAAFGQFLLAAPLICLSMFIIMTSSDATEIVTRILYFGCLSGQLFIYCFCGNLIKTQSDLVATAAYNSHWTSTSVRTQKALHLLVVRGQKTISVVAGNLFELSLVTFGALLKSSYSFFAVLSKQRDE*

>CnigOR55

NMQPPKSPSSRTTEAVVVYQTNARVSLFYKICKIMFFGTGTNFMFGDLELPDNFVKVFNYISKVCELLVIMLICSQWGAFFTQHDLDMISKSQLWMFGPTSLTLYGMYLNGLWRKQQITKLSFTIAVTLRGMFTDDEIEKNMIKKTWRYIYAFSCLMAGLFFSTGIFTGYVACTTNETFTTMIPVWPTLDNHSLAASISRIAHYCIWFIFIIRFGSIYLIILAITIGVQYQYRMMSSYFLGLNSIFEGDNSSQEEKERKYEDGVKHGVKMHCLTLWCIDQTQSTSGVTVSCQLITNIITLIMLMIQWMHMEHTFGNCLNLILFVGVTLTGTGIFMWNAGDVTFEAANLPTAMFHSGWYNCRGKRSVRIRKMVTIAMQQAQKRVVINGFGILELSYESYISMVKF

>CnigOR57

MTITYSTFEAFRPHFNALANVAYFKIVPKPSSGIKHTLHTAYRAVVWFLVIIYNLQHVIRVIQARHSTEQVVNTLFVLLTTLNTLGKQVTFNARIKRMDRLITIIEGPLFAGRNAYDEKVLRENARIMFRLLKMYHGSIYLCGAMWGISPLVSKLSDEVELTGYFPFDTSDWLGFGIAVAFNTIVITLQGYGHVTMDCTIVSIYAQTKTQLQILRNNLEHLVDPVEETGREICVRSTVFKDIEDPAFGVVLKKRLTRCVEHYKQIVWFHEEAEAVFDEALMIQLFVVAWVICMTVYKIVDLSLLSAEFFTMFVYLGCMLGQLFIYCYYGTQVRAESEFINYSIYRSDWVSLSPRFRALLLILMSRGMRSVVPRVAHIVPMSLETYISVLRSSYTLFTFLERK*

>CnigOR58

MKFKSIFPNSSTRYFQMLTQFIYIVTATNFWFVEDVKLPSNFLKVYNLVSKFLEVIIITFVFTGFGASYTQKNLTDKQNTDVFMKSTSSLCIYTMYGCIIYKREEIKQLLFLLMVTLKEIYNDKIEKQMMMKIKIYMIGLMFFGCAAPMMSYGLEGAFHAVTSNATFTTVIPIWPDLEDRRLIAGFTRILIYIVWLLLIAHVMAIYSLIISISICLGYQYVNLCEYFLNLHNIFDGEGSQEDWETRYEEAVKVGIKMHVIILRCVNQLQSSCKVVYGGQVLLNVCVMLLLLIQMMQTDRSLVQLVPIVSTAIAVLVSSGLFIWSAGDITFEAQKLPTAIFHSGWHNCRRQSSVRVRKLITFAMAEAQQAVVIKGLGVVGLIWKAIYYSSFAYPLFSYISVVRYLTIHLLYLNIIFRTVHQTFP*

>CnigOR59

MSTNAEARREIGATLTLCMFSMQCIGLSFERPDSTARLLRQKLMFVASVCTIVYHVFSEIVYIGLTLSNSPRVEEVVPLFHTFGYGALSIAKVFALWSKKDVFAEHLNELSGIWPMEPLDEVARNIKKKSVAALRLVHQGYFSINVGGVMFYNVTPICVYMYQLWQGQDAAVGFVWVSWYPFDKYKPINHVFVYIFEIFAGQTCVWIMICTDLLFSGLASHIAMLLRLLHKRLEMLAETEKSQEEYYQEIVTNIKLHQRLIRYCNDLEEAFTIVNLINVVFSSLNICCVVFVIVLLEPFVAVSNKLFLGSALIQIGMLCWYADDIFHSNADVALAVYNSGWYRTDPRCRRALIFLIRRAQKPVAFTAMKFTNLSLVTYSSILTRSYSYFALLYTMYNNS*

>CnigOR60

MQPPKSPSSRTTEAVVVYQTNARVSLFYKICKIMFFGTGTNFMFGDLELPDNFVKVFNYISKVCELLVIMLICSQWGAFFTQHDLDMISKSQLWMFGPTSLTLYGMYLNGLWRKQQITKLSFTIAVTLRGMFTDDEIEKNMIKKTWRYIYAFSCLMAGLFFSTGIFTGYVACTTNETFTTMIPVWPTLDNHSLAASISRIVHYCIWFIFIIRFGSIYLIILAITIGVQYQYRMMSSYFLGLNSIFEGDNSSQEEKERKYEDGVKHGVKMHNLTLWCIDQTQSTSGVTVSCQLITNIITLIMLMIQWMVSIDTV

>CnigOR61

MSTAKEEFLAGMDYLSVITSRIFLYPFLGRSKTKLLCYHLICILISFASFQQFIFLCVSKLNSFLDIVNIAPNIGVCAMSVTKYIKVNSNKELYNQIFVHFRTEMWDIVSEKCQENLKILKRYQKIIHFITIWFVYYVVPLILIVTSFPILIMYYDNMVLGKELEHRYPFEAWYPFDKVKWYYAAYAWESFITGLVVCIYTFSDLINVSYIAYICLELKLLGTHLKDLIEAEDIKQLKRSQNAKAIHSKIRQKLRGIIIKHNFLAYITSQLDIVFGDIMLVNYTFGSVFICLTAFTFTVTDELYSTLRCFFFLISLVISMLNQCVIGQCVSDHSEQLAQALYDSKWTYGDRQMRQLVLMLIMRMQKPFQLTAKGYIAMNLDTFTTICSTSYQFFNLLRTMYDPKSN*

>CnigOR62

MDLLKSLWWAITNTKALENSSGEMETEFFGTVYRITYITGLSNSDHGFFYKLYSTVVKLMIATCVISEWWYMFAFGSSLDIVIEQMNFIVINTMALFRYGYMRMHEPVYKKLAALCSTLDTTTPARNALRRYWMEKNDTYLKLVLGLGSMNLAVWYVYPLVDGIDYNLSVGMWIGVDFNTPLRYPIAYTIHIVAFHCTAFFVLANDVIIQAHLLHLVCQYSVLADCFENILVDCEKYFKGLNRDQLVRDSRFREVYISRLGLLVEQHQNILMHTMELRKTISLPMLGQVMASGLQICFAGYQVALTLTASYTKFYMILQFLVYNLFALFIVCRWCDEIKIQSENISNAVYCSGWECGVGTMAGVRSSLML

>CnigOR63

MKAFTVIDEPIPKWSYFSLAVNVFDATVAVVFAAISTFHGISLLDISITTEAGVYCIVLIYKFLILTCTQLDKEHYHCFLRVLREDFRYVCAEGAKYRKRFFENQLETWKVSLCSVIFTFGIAVGMGSFALVSLIFYLMTRTPGDGSQRPLLVPFWFFDLDFGKTPVYEIALNFSNFCFLTYAYNYVFMIQTQVVWVRQIATKADLVIWAIQDLLQDIHPATNEEEKVHYAELIKYRMREIVSQHHSMYTLMEAYAGVYKKLLMFEQKLCGPVVCLTAYCTAEKLDEGEFNAILLLLCGATVTLVFIPCYLCTFLGLKVKSISDACWNISFWNAGREIRPYLVLIMQRSLRPLPLQAPGFEEISIQTFSTKMTNAYSLFNMLRQTSI*

>CnigOR64.1

MKTFMSTAKALLYKKDFEWDKEITLQNFHPQLQIFLAINGIFFNNRESKIRFILPVLSTLITMVAVAFEMIFIWRGISTNDYGFATECFCYFFILGSVGIVYSSVLLNRLKVFKLLHNMNNDFIFICHLKAQYRDTFLTGQLLIWRLCWSWIIFISFVSLLYISNTLLYLLYQSTLATQDEHMIRPLIFPMWLPKDDPYRTPNYEVFLVLQAILIFVVLVTFGLYVYILFHLLLHYYNLMDVILIALEDLFKGLDESVVALPREDPRRQEVQDELNIRMAQIVRWHMSVFDSVNDISSVYGPTLVYQVMFSSIVICLMAYQVAEQLSEGKVDYLFGVLGIGACLQLWIPCYIGTLLRNKGFFVGDRCFYCGWHETPLSRLLRADLIIFIQRTQRPVAIKFTGLPHLQLETFSSIMSNAYSLFNMLRQYK*

>CnigOR64.2

MTLLNTLKYYINKEDFDWGRSDITLQLFHPQFELFFAINGIFFSNRESIVRFIWPVLSTLITLIATAFEMMFIWRGITIRDYTFATECFCYFFILGSVSIVYSSVLLNRMRIFELLNNMNNDFIFICGLGREYRKCFLDGQLLIWKLCWYWLMFASFVASMYIANTMCYLLWQSIFATIDEHTVRPLMFPIWLPKDDPHRTPNYEVFMTFEIIL IFIVLFTFGCEYYIFHTRKDTL

>CnigOR65

MTVLTIFRDIKSFVNKDGYDFERPDVTLQNFHPQLEIFFAIKGIFFNNHRSMKRFIWPALSAFMASAATGFELMFIWRAMTIKDYAMATESFVYLMILGSVYLVYYGVLSNRTTILTLLGKMSEDFRCICNLASYRKRFIDGQLLIWRLTMSWGIFVTVVAILYVLNTLLLLLYQYLFATLDEHYVRPLMFPVWLPHDDPYRTPNYEMIMIFQVALILVAMASFGLYVPLSLHLFMHYYKLLDMIMIAIDELFVGLDWSVVTLHVTDQRRVDVKTELRKRMGRIVRWHQSVFDSVEAITSIYGAMLVYQVMFSSVIICLLAYQIAIQMSEGKFNYLFVILFFGAVLQLWIPCCIGTLLQTKALALGERCFYSGWYDTPLSQLVRQDLLIFIMRTQVPVVIKFIGIPELELHTFSSIMSTAYSYFNMLRQYN*

>CnigOR66

MESREYRKNKTTELFRKLDKAIFICSSMNFWVDDNGIPELVVNSYKRISKVINVAIVLFMVAEIGSFFNQNNLTEKQKSDRCLMTFSHIILYSCTLSLWHHRETVTEMLFTLAVGLKKDFNDEATERLMLKRTRIYSSAFVLLCFNALVFYGLEGLASVLFSGGTFVTMITVWPDVHDESLLAGVGRVAIYILWWLWMLRVTTAYLLVLTVTISLSHQYINLQMYFKSIAGIFEEGISQSEKEEKFERALKLGIRLHATTIWCAHQVQRTFGNVFSGQIIVNIGVLVLLMSQFKNSDRAPSHLLAIAATFASMLFGTGVIMWSAGDITVEAENLPTAIFLSGWQNCTNRSSYRIRRLMLIAMAQSQKRVVIKSYGFLELSYQSYVSIVKTSYSIFSVMY*

>CnigOR67

MASLEQLPSSFIDTITTPLNLFRFIGLEFFDDAKARFENYFRFILFLLLSFTFLQGLIFFFVKINELDAGILEIANVIPCLFLVIQSLYKLSIKRKKYLIRNVVYEIAELWPGEVENQEKKEIMDFWVHRNKMICNALLKFTILGLLIFNGISLVTYFILRLLGKDPAYVFPFELHYPFEIDALWKYVGVYLMQTLATTTIGICSYQCCGILQFSLTVNVSMLFRVMQYDLVNINVNRRGLEADESLENLRNIVKRHQRILKLAEDLDEIFGAILFNVLVFSSLIICFFGFLSIVIKVKFQQFMYLTAALEVLFSVFFITLPGQILIDTSSGVADAAYQSLWYNSDQRFRKMIHIMIARSQKPCKLRAMGYTDINFETFYKICGSTWSYLSVVNQMYQNSL*

>CnigOR68

MEHLKDFASEFSKPFAICFDLLGKSNISIYNESKFRGKLRILALVVFYVTFYFSLVVSFKKVFTGELGFYELANLLPIFIVATQGAMKGIVIVSNLSKAKTVIDDLGTMWRSNGLTKTQLMKKGVMLKRLNFCNAVFYWMNITGTWQYILVPLFETVFRNFVLGQDKLLFPFICSLPYEATKNWMVYLVTYFWESYSMLHLIYMYLGVEFLMITLCSHLATEFELLREEMLHAKPILEDTAYTTYKDNIGTVISYSDEEQNDSIDYFEEDSTINIIRADEDGPRIQEVIRRHQKLIMLSQLLDDIFNRMIFFNLLFATITICFFGFVAKIARDPPEMANNFVGVVASMIPIFNLCYYAELLSGASAGVADSAYHNLWYNGDRQYQKIIIFIIVRSQQPCCLTSMRYAQVTLNTFTTVLSTTWSYFSLAISVYET*

>CnigOR72

MSEFTNFEPMFQETYKLILDRIKSNQIYIMDEGPWRERLCWIKLMVNILAAMSHTAGVIERIAQGADLVQLSTDLSAVLILWQASLLYIQLCLNRKLLKKFIIHMGTKWRPDDQLRPHMIAVKHEYVLTFFRRIKVFYKAVTTYVILYLLTPFTYIAVKRFILKDGVALVTPFYIKMPFKFDDNFLLYSLVTFADSKILLDVGYLVCFDLLFMNAAMNHLRLMFVMLQDDLRHVHEEDVKQAEKTLKKLIPLHQDLLKLMTELSNAFGAVFFIHLAFFSGTMCFFGFAARIHCSPESIKNLFAGNIILVCIYTCCYYGQNLTDASIDIAQAAYESQWHLKSQEYKKCILFIILRSQKAQYIKSTSFTDVSLETFTKILNVTWSFLSLITKVYEA*

>CnigORco

MMGKVKSQGLVSDLMPNIKLMQMAGHFLFNYTDETGGMSLLLRKVYASMHAFLIVVNFICMGINMAQYSDEVNELTANTITVLFFAHTIIKLAFFAINSKSFYRTLAVWNQSNSHPLFTESDARYHQLSLDKSRRLLYFICGTTCLSVVSWVTLTFFGESVRLIADKESNDTLTEPAPRLPLKAWYPFDAMSGSMYIMAFVYQIYWLLFSMLIANLLDVMFCSWLIFACEQLMHLKAIMKPLMELSAALDTYRPNTAELFRASSTEKSEKIPEPTDMDIRGIYSTQQDFGMMLRGAGGRLQNFNSPNPNPNGLTQKQEMLARSAIKYWVERHKHVVRLVASIGDTYGTALLFHMLVSTITLTLLAYQATKIDGLNVYAFSTVGYLSYTLGQVFHFCIFGNRLIEESSSVMEAAYSCQWYDGSEEAKTFVQIVCQQCQKAMSISGAKFFTVSLDLFASVLGAVVTYFMVLVQLK*

>CnigGR1

MGVMPIMRVPKDAQTTKRTTYNWISKATFWAYLVWSLESIIVVKVGKERYENFQKSNNKRFDEVIYNIIFLSILIPHFLLPIASWRHGPQVAIFKNMWTHYQLKYLKITGTPIVFPNLYSLTWGLCFFSWGLSFAVILSQHYLQDDFELWHSLAYYHIIAMLDGFCSLWYINCNAFGTASKGLAQNLHKALEADHPALMLAQYRHLWVDLSHMMQQLGRAYSNMYGIYCMVIFFTTTISLYGALSEILEHGLSYKEMGLFVIVGYCMTLLFIICNEAYHASRKVGHEFQVRLLNVNLGAIDHSTQREVEMFLVAIAKNPPIMNLDGFTNIN

>CnigGR4

MDQPKFITKQRKRLQDHQKNNFPHEVIHDDFLQVMAIIFRFSRWFGVAGSGNIMWKTYGLCILLMLGFIEGVAIWRVVRALAGLASDIEGHRSVTARLAGATFYASSITTLILSWKLSSSWETIASYWTSINRRVAINVPTDKKIKNRMITVTSVMVTCVVVEHAMSMMSQVGFDCPPSLILKRYTLMSHGFLLLRTDYSIWFAIPLLFMSKIATILWNYQDILIVLISMGLTSRYNTLNQYVAKFSTHIQDFPWNPHGECSKGYMWRRIREAYVKQAQLVRRLDESLGGLILLSNLVNFYFICLQLFLGITQGLSGDLIKRLYYVVSLVWVCVRVSCVVLAAADINVHSTKALRHLHASDRHYYNVEIVRLQSQLSKDYVALTGLGFFSLNRTILLQVHGRSNNHV*

>CnigGR6

MFKHKKSSKLFFVSETKLTNDKSEALRHYNVKLQPEIFMTPLLTGDYNGYQATFQEAMKLTIIIGQFFGLNPVSGVSETDPNKIKFQIQSCRFVYSLLSIIGQFTVVFFCFLKLFTDSKPNLSANSALVFYISNGITTILFLRVATRWPRLCQLISKTEMSDPSIDRKLIKKCRVSCVLVLTMAVMEHVLSDLSSIAGIIDCQKGENVYEAFVVASSPWVFQYTGYNPYVAIFTQIISIQFTFNWNFSDAFVICISFYLTSRLEQVNRRIESVFGKHAPSSFWRTLREDYSRITGLVRRVDDVIGSIIFISFANNLFFICIQLLHTLAEGIRRNPSCRLGDPDE

>CnigGR29

MGHTSELVLRFLRNYFPLKSILLVRAFFGHGYCLKCPKIYLKLQKVYCVVVVLAYVLGVTNFTINWRRWMVYEAVIATLTSCFIEGDYCGKFFSFVYRTDQSFSHYRLYLASCRIYVVFFVTVSIRLYMDFSNIGALLVVPMHYLILLFAYASIDFSHFLRIIVFDILYERAMHLRNHFESIFLRVYGDDSMVSEIRQGILLYKELIQSVTLLEKIQVSYVIALVTRFVVNIADIHDVFYMYINDQVIYHNIRIVGCECVYYAVFICIPAFLMELVHNEVDKITSILTMQYGISTDREVRVAISRGLRYMQLRPL

>CnigGR30

MGDTFDLVTNFLHHYLPLNTIFCIRAIFGHFFNFSCNKLYLTIQTIYCSVLSFVIPLLLYYFGKLNNTVQIIPVSEYFIGIWVTLVVENDCYKKFESAIQHTDQCLGLYRLNLESFRLYAVFFSMVIFRTCITLVILQVYSDNSLSILVFHFMYISLDLGHLIRISVFDVLYERILQLRKYFERVFNGPPGDYTNMPSDLKTGLLAYKQLLDSVELLDNLQSTYLVFLVLSFFNCVCRFTKNTIDVFRPNLDWLSWISFLLDAIYAVVLIFTPPVFVELIHKEVDRISWILTVQYSTCNDINVRAAISKGITYLKLRPFEFRIWHTIPVDSSLPLSFMGLAFTYILLMLHVNKIL

>CnigGR60

MFDNAIDRDFQIMLLPLNVLEFVYCQPKFRITQTFIIPNGKRECLLNTLGVFLMILTNVVYVSLNSYVPGNNQISDIIRNFFCADAAFYVVYSLLLYAMNIINRDKNVQLIIKMQSAYRVLRNENGLKRFQKGNWIFSLGVFFMYFTYNLSYTIFKIYRVTHLMYGMVLFYFDVNIIVVIRIVKFLEYELLLWKKELNEFLITCSKTNHEQLVKYLKDVSYEEKLLLSAYSNIIGAFKLCCSVFGMSVSTLLIPIK

>CnigGR63

MRVSGVFRIISADRRNTEIQNTFKPIKILTRIVSLNGSGANTSWQLFWIVFKALASASVLGFLTFYCLYTKIRYHYKDVLLSIKLTDTVQMCYDYTIYLVDLFFVFKYGRETYAEYHKHIISIDQILLTTDYSAIKSRHIKFIAYFIVIWIITSVCDFAAWALSYGILLPTLYSTSYIYLLIKMFTTLDLMCHVIHVEYRLKCIVNQLQECYCDTKRFPGDFSDPIDNKLWFYCPSKHGNTDETNPDSTSAGSNPQAVKWLSRCYLLLCEQCVFINNMYGTRILLSSLSLLIDMIRFTNIAVRLVTGSQPTMYASGNYPAAANILRMVTCALVV

>CnigGR68.1

MSYFKKIRITKSYKINIWSAFKPLYYILTLLGLLPYSLKFPKGSGGATIIHKSIYFNSLCSVSMILILYTFFVLHTQQVFASTESNTMTEVLTTKFNYMLEMLTLLLFATVSYFCAYKNRFTYVKILNAIVSSSYKFVDHKVFIRRLQVQVKIVTLCLFLLLFLQITINFTRFDTVRKMMLVTSSFILPQMIQFTVIGFYYVLVLMVVGVLKNINEQMKNLCKYSRVNAEFIKVEKRITLNQIEVVYVNMLEMKRAINRAFQASILATAFQCFHSIVSESHILYHGLAVDPTLTTHDVCNCSIWIVYQLIKIYTISCSGNMLKEQEAKIGRSLHNILAGKEDARLYLEVQHFSSMILFQN

>CnigGR68.2

MIITVHTFLVFHLQELNASSKDNSMTEDKMTLMSYIINLVLQILFCTVSYFHVIRDKNVYIAMLNEMAVCWDRFAMGKRRLILGRLRVQINCVVLPTVLAMISVLTIATYIGYLGIWKMILITLTFLLPDLIQFTMIAFYLVMILMVVALLKNIEEEFKVISLVKNNVPNDLVEAHLVVNINEIREVYVKTLGIKRQINEAFQAPLLVAMVVCFHELVTMPHLIYHGLSFQANFTTHDAIECSVWVLNQLLKMYAIAKSGALLKSQVNEIGRTIHNIPIRGDRDLTMYLDVLHFSSLMTYQDTAITLYGYFPLDSTLVFNIVASAAMYLVILVQFDKPE

>CnigGR68.3

MSSTLTKYFSPFVNPEEELCLLQIFKPVYVLVSALGLFPQSIKFPDGIYKTNVEFKNSAINSACTFFMIVVIHGAFVLHLQELNISSNENSMTENKMTMINYTAALVLQVLFCTVSFFRVVYDKKLYITILNDMADCWEKLAMGKRRLILGRLRVQMNFVVLASVLMSFVLLFHCICIGYDLSIWKVILTCLTFTLPELIQFAMLTFYFVMVLMLVTLLKNIEEELVLFLHVAKNNRNNFVESDMRMNMGEIMTVYVKVLGLKRQMNASFQSSILVALVTCFHELVSLPHIMYHGLVFQSNFSTSNIIQCCSWALNQLLKIYILGRSGDLLTSQVNEIGRTIHNIPISGDLDWKTILEVQHFSSLMAYQDAKMTIYGYFPLNATLLFNMVASAAMYIVILVQFDEPE*

>CnigGR68.4

MAKANYVIELTTAILFCNAAYFCVYRKKDVYVLMFRDIARAWDDLHYVNRNAILGHLRVQINCILGILILILITLTATTFAGQSSLWKRILITFSFNIPEMIQFIVVAFYFVFILMIVSLLKNIESHCKMFKKVRRIINGSNVELVRVPVTLSQMQTVYMKTMSVKRQVNEVFQAPILFTLLQCFHTTVSEACDMCLGLLYKEDFSIHNIVECSFWVMLQMIMVYVLARSGSLLKAEANKIGRVIHDIPISEEEDIYLFVEIQNFSTLMSFHDTEISIYGFFLLEAPLMFNIVAAAAMYLVILVQFAE

>CnigIR1

MWRLLFLVASAACLPTATDWPNMAVEYFRHKGVKYVTHLSCKDAAGIKAAWRLLMNEGIRATVGLIDHGPVNITRLLYQYEASVGILVDGDCLNTRDILNNASDLMMFDHMHSWLVMNDNCSNTGFVSDTFQDLKLSVDADVVVASYCGDTYQLTDVFNFGRVQGNALETRGLGAWTSKKGLKILLQGFKYYNRWDFHNLTLRAVSVIRNGSKEFHEGMLYEPGFTVGVAAMTKISSQLLNLVKEIHNFRFNYTIVGRWIGTPERNSTKAMSNMLLWRDQDISSTSTRMLSIWLDWMDPFFPAVTELQTKFYYTISDKGIGDYENEFLTPMSPGVWWC

>CnigIR3

MCVLTCGDRTWNKKIAKHASKVSIAVSHVGIDESLLDLGSVGRCLNPGYADVGVLIDTKCPLYEDVLMYASEKLLFDSNHKWLIIDIDTWISNKSTVSKVEINENLSWLMDTFGKLNLSVNADVTLALQKGSENTIYEVYNFGKLRGGNVVAKKLGNWRNRADVINHLNAYKYYRRWDFENSTINFVAVMSTPPKVFDVNMLIGDTPAPGVAVVTITGTRVLVEIAQLHNIRYNYTIVDRWIGKFERNATPAVATLVYFNEQDVTPILRVTSEVFERVDMVSRPVTSIETRYYYRIPTTGPGKFENQFLRPLTNGVWVCVMAVIFLCALLLFLTARAERRPAAIQYAIFTVAATFCQQFFEDGGVNDPGRDSSARQLTVLVTGTSCVLIYNYYTSSVVSWLLNGPPPSINSLQELLESPLSLIYQDIGYTRSWLQNPTYYYNKKNAEVEDKLRRFKVSKKKKGEPLLVPLEKGIEMVKAGGYAYHTEVYNANMLISRKFNQEELCELGSLQSMEKTNLYIAIPKNSPYKEFWNWNILRMFETGVVSHLQRQANSPQISCEGSSPRALALGGAAPAFLLLAFGYLLATIIVLIERLVARKRITHRH

>CnigIR7d

MYQCPFIVSTFEQHPFMYLSNNSAPAGKDGDLLNLVVDILNASLVIKTPKDGSDWGKLVNNNWSGSLGDVFNGVADASMCSAPLSPTKYANFQISFTYSSMDIVWAAGLPALKPGWEKLLYPFQMTVRVIIFFLFIGIIFMNTFTTTRFWRKATRAFRLSPPKSNLLFYSWIIFMGLPVVRMPSKPSLLIVIGLWIWVSFIIRTVYQAELVTIMKQRIYEEPFETFQDALATKKHFGGLSMYKEYYSDDKYIYENYIDKNLTEARHTVDKISNGSLDFVIAMKKESVFFRLMEFKGTRQLQIIPKKIANSPTVIFFKKFSFLANPVSRILSVSMEGGFSQRMYSRFFARAVSLFHQSKKQGPAALQLEHFKGSFIILFGGWILSAIFFAVEVICGKIVNANVYS

>CnigIR8a

MESEADVDRTLYELLGESNVRVWVHAGLTRDSARALKTMRPEPSFFVLVGSGAFVTDTYKRAVKEKLVRRDYRWNLVLTDYSTLDLQPVKPAMVLQVDPAECCKVMGQKDGCSCSQDFERKQPILFALLQLLAETYSKLDDDEFTARVDCDNLVAENGTRSKVYHQLAEELGASNESLFYWDADRSGIFLRSRFILSTLKPDTGLQHAAIWSADEEYKLLPGVTLEPLRQFFRIGTAPAVPWTMPKLDPSTGEPMFNEDGEPMYEGYCIDLIQKLSESMDFDYEIITPRTGSFGRRLANGTWDGVVGDLMRAETDIAVSALTMTAEREEVIDFVAPYFEQSGILIVIRKPTRKTSLFKFMTVLRTEVWLSIVAALVLTGFMIWLLDKYSPYSARNNPDAYPYPCREFTLKESFWFALTSFTPQGGGEAPKALSGRTLVAAYWLFVVLMLATFTANLAAFLTVERMQTPVSSLEQLARQSRINYTVVEGSTIHQYFINMKFAEDTLYRVWKEITLNATSDQSQYRVWDYPIREQYGHILLAINASMPVPDAKTGFRQVDEHTDADFAFIHDSAEIKYEVTLNCNLTEVGEVFAEQPYAIAVQQGSRLQEELSRALLDLQKERLLEQLAAKYWNETARQQCPDADESEGITLESLGGVFIATLFGLGLAMITLAWEVFYYKRKEKNKVRQVEEENKPKKAFEKDLEKKIAGGVARLRKRDKKDKKGQVTIGDTFKPVSEKDGVSYISVYPKTEYKP

>CnigIR21a

MWLLRTVFCNYILFHYIESQEIEYYPSQASSFAEKLVSELKSEPYKYKHDLFKREAQWRKFNNNDNSELTKNKTQKRSVDPVFHGHPKTREELWNERIINESLTFDQTPSLISLIHNITLTYLNDCIPIILYDSQVKSQESNLFQNLLKDFPIAYIHGYINESNELVEPKLVRATKECIHFIAFLTDVTKSAKILGNQAESKVVIVARSSQWAVQEFLAGPQSRMFINLIVIGQSFKNGDDDALEAPYILYTHKLYTDGLGASQPIVLTSWSHGKFSRQVNLFPRKMTEGYAGHRFVVAAANQPPYVFRTIKTDADGGNPRVVWDGIEIRLLTLLSQRNNFSIEIKEPQELHLGPGEAVLKEITTGRADIGVAGIYLTSDRTRDTDMSFSHSSDCAVFVTLMSTALPRYRAILGPFHWTVWLALTLTYLFGIFPLAFSDKHTLRHLLHNSGEVENMFWYVFGTFTNCFTFVGKNSWSKTTKITTRLLIGWYWLFTIIITSCYTGSIIAFVTLPVFPETIDTIEQLLDGFYRVGTLDRGGWEKWFLNSSDPKTNKLLKKLQLVEDLPSGIRNTTKTFFLLPFAFLGSRAEMEYIIQSNFTKTKKNKKAQLHISNECFVPFGVSLSFPNNSLYSSKLSGDIARLLQSGLIDKLENEVKWEMQRTPSGNFLSAGSGTLKLGPITEKGLTLADTQGMFLLLAAGFLLAAAALISEWMGGCSRKCRPQKKEDLPSSGHSREHLIPSPKSDVESEIKVISDSAESRFRLHPRPNSEDSRDSLEGAIINVTKESIIIHDNFHASTDCWDSRRSSSVDIDKEVQEIFDKDEKRRRIKSGTVPLTDNQREATASKGAFGDHLSDH

>CnigIR25a

MSSLTILLLFLFVPDSFSQTTQNINVLLINEENNALAEKSFEVAKEYVRRNPTLGLAVDPVIVVGNRTDAKAFLENVCRKYNDMLSAKKTPHVVLDFTMTGVGSETIKSFTAALGLPTISGSFGQAGDLRQWRNLDANQTKFLLQVMPPADILPESIRAIVTKQDITNAAIIFDEFFVMDHKYKSLLQNIPTRHVITPVKSFNRDEIKTQLRSLRELDIVNFFVVGSLRTIKNVLDAADENQYFGRKTAWFALTLDKGDISCGCKDATIVYMKPTPDAKSRDRLGKIKTTYSMNGEPEITSAFYFDLSLRTFLTVKSLLDSGKWPNDMRYISCDDYDGKNTPNRTLDLKTAFHEIKETPTYAPFFIPEDDPMNGRSYMEFNTDLSAVTVKDGASIGSRNLGSWKAGLSNPLSLTDPQNMSDYSAQLVYRVVTVEQKPFIIRDDDAPKGFKGYCIDLIEEIRQIVKFDYEITLVPDGNFGTMDENGNWNGIIKELIEKRADIGLTSLSVMAERENVVDFTVPYYDLVGITILMKLPRTPTSLFKFLTVLEDDVWLSILAAYFFTSFLMWVFDKWSPYSYQNNREKYKDDEEKREFNLKECLWFCMTSLTPQGGGEAPKNLSGRLLAATWWLFGFIIIASYTANLAAFLTVSRLDTPIESLDDLSKQYKIQYAPLNGSAAMTYFERMAHIEVRFYEIWKEMSLNDSLSDVERAKLAVWDYPVSDKYSKMWQAMKEAGLPNSVEEAVQRVRDSESSSEGFAWLGDATDVRYYVLTSCDLQMVGDEFSRKPYAIAVQQGSPLKDQFNNAILQLLNKRKLEKLKENWWNNNPEAMKCEKQEDQSDGISIQNIGGVFIVIFMGIGLACITLGVEYWWYKWRKRPVIGDVTQVEPSKTTRNNTDNSTTKIGEGFTFRSRNMGLSNFRSKF

>CnigIR41a.1

MIIPSILLPIEILLNILINEQLQESFCLTFVTETKLTVKIPKNVSSMIIQPNNSVLVEQILDTSEKGCSDYIIQMHEPENFMIAFEKVNHLGDIRRSDKKLIFLPFQDDLDNSSILTDILSLVETGFVANILLIVPSLQSSGDCMVYDMITHTFVGADEEVQMPLYLDRWDSCTGHFERGASLFPHDMSNLYGKTVKVAAFTYKPYVLLDLDPSLNPLGRDGMEMRIIDEFCRWVNCTVEIIRDDAHEWGEIYENNTGVGVLGNVVEDRADIGITALYSWYEEYRVLDFSAPIIRTAITCVAPAPRVLTSWDLPLVPFTWTMWICLLFAFFYASFALSLAQRSTDNIFLDTFGMMITQTREDATSWRIRSITGWMLVTGLIIDNAYGGGLASSFTVPKYEPSIDTVEDLVDRGMEWGATHDAWIFSIILSEEPLIKSLLRQFKTYPADILKQKSFSRSMAFSIEHLPAGYFAIGEYITKEAAMDLEIMLDKVYYEQCVVMLRKSSPYTAKLSELVGRLHQSGLMLSWETQVALKYLDFKVQLEVRLSRARKDLEEIEPLSLKQLLGIYIFYFGGLALALLVFFGELLFKCSKPSIVL

>CnigIR41a.2

MLIQSNILFPIEILLNTIINNYLRTSFCLTFVTETELLINLPLNMTSIRIKPNNSELVQQILEASEKACTDYIIQMDEPQNFMVAFDRVNHIGDVRRSDKKLIFIPLQDEFYNASVLTDLLSLKETGYVPNILLIAPAAQSSADCKIYDMITHTFVGADEQIRDPLYLDHWDSCTELFEKEANLFPHNMSNLQGKTVKVGAFTYKPYVLLDLDPSLAPLGRDGIDIRFIEEFCRWINCTVKIVRPDDGQEWGEIYDNNTGIGLVGNLVEDRTEIGITSLYSWYEEYRALDFSAPIIRTAVTCIAPAPRILSSWDLPLVPFSWLMWMCLIATFFFASFALFVAQRSTDDIFFATFGNMIGQSPGDSSSWRIRSISGWMLLIGLVIDNAYSGGLASSFTVPKYEASVDTIQDLVDRRMEWGAPVDAWLYSMILSEEPLIKSAISQFKVYPPEVLTKKSFTRSMAFSIERLPAGSFAIGEYITKEGAKNLELMVEDMYYEQCVVMTRKSSPYTAKLTELVGRLQQSGLLLCWETQIALKYLDFKVQLEVRLSRTKKDIDGVEPLNVKQLLGIYLLYFGGLSISIVVFIAELLIKRSKAVVLI

>CnigIR60a

MLKIIFLLSVGVKAKVNPHGPTVVSDYSSCVIDIVDKNFDQSGLLYFVDTFNVSTPVVGIRHGIIKSLHKKMKYSVKVSAPTKRDKGICIKNDNRAIERNVNSLMDQFQAFETLADYVIAIIEEYKDFTYIASRLIRARSWNPRALFILVYFSISGSNDQNIRHAEDMLFCLFKVNVINAVVIIPEGNNVRKANIYSWRPYEPPKYCGYYNESIRNRLIVENVCEQGKVKYAKKIFEPKIPSDMMGCSLKVLALERQPFVSHNPLDPNIERLLIDQVAQRYNLTLQYEVLNEFRGEKSFHGDWDGAIKELTYKKGDLLLGGIFPDDEVHQDFECSSTYLADSYTWVVPRALPRSAWLSLFIIFQKTVWLTVITCFVLIALSWMVLAKLSKDASYRTNLDHYFINTWLSNLGFCGFSRPMTNSLRIFFVFINLYFILFLTAYQTKLIDVLTNPSFEYQISTVEELVESGLQFGGSEELHDLFENSTDDIDNYFLDGWIDIADIKNALRDVAIHRNFSLMCSRLELAHVSAIIPELSDQYGNYMYYAFPTNVFTVPLETVSMKGFPFMKGFSSTLTDFRQYGVNKAVLGYFAGYLLRQRAELLDKLESEYSKRDALSIQTLQGGYFALILGTLCGTFVFIVEIILNTKIVKNIKFHKKFLYMVFVFYLPKSNY

>CnigIR75p.2

MKILFLYVIVLFLRFGKAFDDNDISMLVSFVTLDDRSTAVLTPYVCWSAYELTSLAKSLHDNGISMAASMQPRRPEIFLQNLVIVADLRCRGTDEFLIKASDEGLFKSPYRWLLISQHQTELNVLDQLAMLVDSDVVIAQRRGGDYQYVEVYKIIENSQLIYNTRALWRPIDKKPNNTTVITYYNKSKAIATKYGIVEDYRKSKILSIRRMDIKKHTLTMVNVITDSNDTRKHMDDRLNLHQDSITKMSYMVVKICFEMMNSTEKLLFSNTWGYVDKNGSWNGIIERLIKKEGDIGTLTIFTQERMKIIDYIAMVGTTAVRFVFREPPLAYVSNIFALPFTGAVWLAVFVCVLACALFLYITSKWEATMGMHPMQLDGSWADVLILIIGAVLQQGCTLEPRRAAGRLVTLLLFVALTILYAAYSANIVVLLRAPSSSIRSLQDILNSPIKLGASDFSYNRYFFKKLNEPLRKEIYNKKIAPKGKKENFYTMKEGIEKIRKGLFAFHMELNPGYRLIQETYQEDEKCDLVEIDYINEIDPWVPSQKRSPYKDLFKINFIKIRESGIQHCIHQRLHVGKPKCLD

>CnigIR75q.1

MERFVTIIFAICICSSFALSISKENDIHVIVDVIQSFNKPSDVIANLCWANNIKNKLAVSIAEASNPRSIKYVSDLHDNNFVSPEKVIFLIDVSCKETKEFLNEAASRKYFERPHRWFVINKPAAEFSIPIEIDEMNMLPDSEVFVMQLINDSYYVNLIYKIKPNREWILENYGNWSAERGLTVSRRAAEVALALRRRDLARATIVTSMVITDNDSLADMELLRYKQIDSIAKSGYHQLAPLYEFMNARREFILTEQWGYRVNGTWHGMVGHLADGKAELAGAVLFITQERMPIIEYLSHPTSSWVKFVFREPPLSYQNNLYLLPFRASVWCCIGAFVLVLIVAMYVNAYWEAKKLSNEKMEDDTMVLSPTIGDVTVFVMSAISQQGSNVELKGMLGRFVIFTLFLLFLFLYTAYSASIVVLLQSSSNQIRTLTDLLNSKLELGVEDTPYNRYWFMNEKEPIRRAIYEKKIAPSGSKPKFFDLTEGVLQLQKKPFAFNCNVGVAYKLIERYFFEHEKCGLQEISYIQDDNPWQAARRGSPYREIFKIGLLRNAEFGLNERTNRIMFSKKPVCSVRGGSFVSVSLVDCYPILLLLLYGMILGVVFLFAEILHHRKMLNT

>CnigIR75q.2

MKVLRLVVFLFIIRVGCHAEEEASKPAMVADVVRAMQRPSAVIAMLCWASNLKLQLYSALEGENVTQITMMQFLKAGTVPERHAQDQHIVFLADLDCPDIVSYFRTSSLNKHFRSPFRWILIDSGNNDTKESYIPNAVANFDILVDSEVILAHHMGNGSYRLHLIYRIGNNTIWKKESYGTWDDRRRLQKQVMEGEIILRRLDLESYELAICYVLTDNDSINHLYDNVNDHIDTITKVNFPTTNHLFDFLNASRKYVFANTWGYRLNGTWNGMTGYLVREEVEIGGSPMFFTSERISIVDYISSPTPTRSKFVFRQPKLSYENNLFLLSFRASVWYSSIALLLLLVIVLFIVTIWEWKKTRGHEDKKVEADSGILRASVVDVVLLIFGAACQQGSTVELKGSLGRIVMLILFLALMFLYTSYSANIVALLQSSSSQIKTLEDLLHSRIKFGVHDTIFNKYYFSTATEPVRKAIYETKVAPLGSTPRFMPMEEGVKKMQKGLFAFHMETGVGYKFVGKYFQESEKCGLKEIQYLQVIDPWLAVRKNTPYKEMFKLGTKRIQEHGLQSRENRLLYEKRPKCSGQGGSFVSVSMVDCYPALLVLFYGAVFSVGLLFIEILTKRRNDILRKISRAKTLSVDVGDY

>CnigIR76b

MTGLELIVSTICNATFCEVVYDNPITETSLTKHKKELLEIAEDLNGKHLKIGTYDNYPLSWVHTEDDGKLTGRGVAFIVLEILRERFNFTFDIVTPAKNYEIGVEGKLDDSLIGLVNNSQVDMAAAFLPIVYKYQQYVEFSSVLDNGVWMMMLQRPKESAAGSGLLAPFEIQVWYLILAAVLSYGPCITLLTYLRSKLVEDGEKNISLSPSFWFVYGALLKQGTTLAPEANTTRILFTTWWLFIILLSAFYTANLTAFLTLSKFTLDVEYPEDLYKKNYRWVASEGSTVQYLVNDVDDELYFLSKMVTNGRAQFRSVHADRQFLPFVAGGAVLVKEQTAIDHLMFEDYLKKTKDKVPEAKRCTYVVAPNPFMEQLRGFAFPRNSKLKLLFDPVLTYLLQSGIVTYLEHRDLPSTKICPLDLQSKDRKLRNSDLSMTYMLMAVGLATAIAVFVGEMIIRYYVRIKIRKNRGERRKLKTPKTSKHKHFKIPDDSHPPPYDSLFGNNSRYKRNGHSTTKIINGREYWVVGTVSGDTRLIPVRTPSAFLYQRDK

>CnigIR87a

MHSTLLLSYLCFIHFVSVKSENPMLMTSGDSGQIEETAECVLKLSAKYFVEKKALSGSIVIININSYASTTQRLLLRTIHSGIKYSVMVKDSFYKHANASHFPEKAKNYMLILEDKSELVRNILQLNKLPTWNPLAKAIIFYKLLSDEDGETVAKIFINELREYKLLKSIVFIYSSEDAEVISYTWAPYSDTNCGGECDSVYILDICKNRIVRQKNPQREMFPLDMKKCPLVTQAIISEPYVMPPVRRLTNTPYPDAYDFQKGGEINLVKLISEFTNMSLIVRISDVPENWGVIYPNGTATGAYGILRNDSVDLVIGDIEVTRTIRKWFHPTVSYTQDEMTWCVPKSAKASTWNNLVIIFQWSTWVATLLSIVIMGIIFHYIYYRENNRKVTKLPTNSLLYTFSMILGWGASFKPKTATFRILIFAWLCFGMIMSISYESFLRTFLMHPRYEKQISSETDLIQSGIPLGGRAIYRSYFETNNASSFYLYRKYISTSFSEGIKRAALERNFAIVASRCQAEYQDQKLGKGEQLIYCFKEGSNLYKYGVVLLTRRWFPMLERFNNIIRSVSENGLIDKWNQELFIHTVSVDGTSKVVPLGIRHLMGAFIFIGIMYAASILVFVVELLLNISKKRRGNNPIAMPVYRVTVSKGGNKSKLEVSDYETGSDLFS

>CnigIR93a

MRIWVVLICIVSVRGEDFPSLITANASIAVVLDRQYLGEQYQPLLDALKDYIKELARVELKHGGVVVHYYSWSTISLKKGFIAVFSIASCEDTWSLFSRTEEEELLLFALTEVDCPRLPPDSAITVTYTDPGQELPQLMLDLRTTRAFNWKSAVILHDDTLNRDMVSRVVQSLTSQIDDEDVPTISVTVFKMKHEVNEYLRRKEMHRVLSKLPVKHIGENFIAIVTSDVMSTMAETARDLLMANTQAQWLYVISDTSIRNSNLSSLVNELYEGENIAYIYNITDDKDCKNGLMCYSEEMMNAFISALDSAVQEEFDVAAQVSDEEWEAIRPTKIQRRDTLLKYMQQHIAVNSACGNCSTWQAMAADTWGSTYHSYGQAENTLVPDNDTSGAIQKIELLQVAYWRPSDGLRFTDYLFPHIVHGFRGKVLPIITYNNPPWTILKANESGSISSYSGLIFDIVDQLAKNKNFTMKLIFPGDLKDILSNKTMTDDMYSQSAKLTMMAVARKQAAFAAAAFTVLSDRNPGINYTNPVSTQSYTFLIARPRELSRAMLFLLPFTTDTWLCLGFAVILMGPTLYVIHRLSPYYEAMGVTRQGGLATIHNCLWYIYGALLQQGGMYLPRADSGRLVVGTWWLVVLVVVTTYSGNLVAFLTFPKQEVPVTTVAELLENRALYTWSISRGSYLEFELKNSDEPKYVSLLKGAELTPDSSGLEGNLASGSPLLSRVRNDRHVIIDWKLRLSYLMRAEHLATDKCDFALSAEEFLDEQVAMIVPAGSPYLPVFNKEINRMQKAGLITKWLSAYLPKRDRCWKSSSVMQEVDNHTVNLSDMQGSFFVLFLGFFSASSVLLMEWFYHRRKSRKEDDAIKPYVE

>Cnig-iGluR3

MRRNANVNASRMLDCNPKGDKIMPYEHGDKISRMIKKTEIDGLTGVVRFNEEGHRKNFSLQVMEMTVEGEMIKIATWYDNKGFVPVVPKLPGPSIPGVYNRNKTYIVSTIEEPPYIMRQNSDYAEFPPNDPYKGFCVDLARMLSDKLEIKYEIRVVKDGKYGNENPKIIGGWDGMIGEILRKEVDMAIAPLTVTVERETVVDFSKPFLSFDIKPSLKNIADEPGAIFSFLNPLSTEVWLCLMFSVLAVTVVLFIVSRFSPYEWRVVSYTDTQSSEHAEVATTKTTVVNEFSFWNSMWFSVGSFMQQGSDITPRSVSGRIVGTVWWFFTFIVISSYTASLASYLTLQRIAEP

>Cnig-iGluR4

MQQPNKFPVGMLGIHFDTSSSSLIAEIATAVKVFAYGVESYILAPENARHPLGTRLSCSGAGASEARWSTGERFYQHLRNVSVESEASRPSIEFTPDGELRAAELKIMNLRPAMGEQLVWEEIGTWNSYPKERLDIKDIVWPGGLHTPPQGVPEKFHMRITFLEEPPYINLAPPDPISGRCILDRGVICRIAPEAVVAGLEAGTAHRNSSLYQCCSGFCIDLLQQLAEQLGFTYELSRVEDGRWGTLQHGKWNGLIADLVNKKTDMVLTSLIINSDREAVVDFSVPFMETGVAIVVAKRTGIISPTAFLEPFDTASWMLVGAVAIQAATFSIFFFEWLSPSGFDCSTGQDSNRAPQNRFSLCRTYWIVWAVLFQASVHVDSPRGFTARFMTNMWAMFAVVFLAIYTANLAAFMITREEFHELSGLDDPRISRPLTIRPPLKFGTVPWSHTDATLAKYFQEPHAYMSQFNRSTVGAGVQGVLTGELDAFIYDGTVLDYLVSQDEDCRLLTVGAWYAMSGYGLAFARNSKYLSMFNKRLLDLRSNGDLERLRRYWMTGTCKPNKQEHKSSDPLALEQFLSAFLLL

>Cnig-iGluR8

MKIVEMKGLTGVIKFDHQGFRSDFTLDIIELTRDGLQKAGIWNSSEGVNYTRSYGENQKQIVEILQNKTLVVTTILSAPYCMRKEASEKLTGNAQFEGYAIDLIHEISKILGFNYTFKLAPDGRYGSFNRETKEWDGMIRELLEQRADVAIADLTITYDREQVVDFTMPFMNLGISVLYRKPIKQPPNLFSFLSPLSLDVWIYMATAYLGVSVLLFILARFSPYEWDSPRNCLDEPQVLENQFTLLNSLWFTIGSLMQQGSDIAPKAVSTRMVAGMWWFFTLIMISSYTANLAAFLTVERMDSPIESAEDLAKQTKIKYGALKGGSTAAFFRDSNFSTYQRMWSFMESARPSVFATSNKEGEERVMRGKGAYAYLMESTTIEYVVER

>Cnig-iGluR9

MDILTDKWYGGLPCFKLSQDYGIQPKPLGVAAVAGVFLLLLVGMIVGFLILILEHLFYKYTLPVLRHQPKDAVWRSRNVMFFSQKLYRFINCVELVSPHHAARELVNTIRQGHFTSLFQKSVKRKEHEQRRRRRSKAQFFEMIQEIRRVQQGRDKSLDSIKEHVAIETSEVSEELTESKFLSPSPEVPSRSPRPGRSPRQLRSPRGRRKRCSLAGLNVRRFSTDSVLGSDSGSIYERTSLNIGRRLSRDVSCLTNSPPDINTRLRTPSPMVRRAEGSSTRSYQDVSERSEKSERYLSSDGPRSRASVEILVSEEQDVPPAPPYPRVSPTGGRSELSQLSEEELIRLWRSSEREVREALLAALQERRANLDPKQDPG
